# Comparison between simulated scenarios and Swedish COVID-19 cases throughout the pandemic

**DOI:** 10.1038/s41598-025-08682-z

**Published:** 2025-07-02

**Authors:** Hatef Darabi, Ilias Galanis, Federico Benzi, Gerard Farré Puiggalí, Philip Gerlee, Torbjörn Lundh, Lisa Brouwers

**Affiliations:** 1 The Public Health Agency of Sweden, Solna, 171 82 Sweden; 2 Mathematical Sciences, Chalmers University of Technology and University of Gothenburg, Gothenburg, 412 96 Sweden

**Keywords:** COVID-19, Scenario analysis, Simulation similarity, Time series comparison, Epidemiology, Statistics

## Abstract

This study assesses the accuracy of COVID-19 scenarios for new infections produced by the Swedish Public Health Agency (PHAS) from December 1, 2020, to March 20, 2023. We introduce a Similarity Error ($\:SEr$), which evaluates the dissimilarity between simulated and observed case time series using the following attributes: area under the curves, peak timings, and growth/decline rates before and after peaks. Rather than using an arbitrary cut-off, we used a threshold determined through Receiver Operating Characteristic (ROC) analysis, with performance evaluated using the Area Under the Curve (AUC), based on true positives identified by visual inspection for categorization. To further evaluate $\:SEr$’s effectiveness, we conducted a sensitivity analysis across the full range of possible threshold values within the unit interval. Applying $\:SEr$ with an optimal threshold determined through ROC-analysis 7 rounds out of 11 rounds were classified as having one or more similar scenarios, including the 6 rounds identified by visual inspection. Our findings indicate that, despite the challenges of a rapidly evolving epidemic, PHAS delivered simulations that reflected real-world trends in most of the rounds.

**Supplementary Information:**

The online version contains supplementary material available at 10.1038/s41598-025-08682-z.

## Introduction

The COVID-19 pandemic profoundly impacted societies across the globe^1,2^. The rapid and widespread transmission of the virus underscored the urgent need for effective public health resource management and the ability to anticipate and respond to emerging challenges. Understanding the development of the spread became crucial for governments and health agencies to efficiently allocate resources, implement timely interventions, and mitigate the public health impact. Numerous dedicated modelling teams from diverse disciplines were established to provide insights for policymaking^3–7^. Consequently, the prediction of COVID-19 outcomes has been extensively examined utilizing a range of methodologies, including time series modelling techniques, machine learning algorithms, statistical modelling, and compartmental models, either individually or in combination thereof^8–28^.

The Swedish Public Health Agency (PHAS) was tasked early in the pandemic to simulate scenarios for how COVID-19 spread might develop in the future, resulting in overall seventeen simulation efforts, amongst these, thirteen focused on new infections covering the period from December 1, 2020, to March 20, 2023. Utilizing epidemiological modelling of disease progression over an extended period for reflecting true case counts is inherently challenging^29^. Nevertheless, such models are essential for illustrating potential trends, including peaks and lows in case numbers based on key assumptions and uncertainties^30^. In doing so, they help anticipate and prepare for a range of possible futures and ultimately assisting in proactive decision-making and effective intervention planning for disease control.

Our objective is to conduct a retrospective accuracy evaluation of epidemiological models that have contributed to policy-making in Sweden, by comparing simulated cases to observed cases, across different PHAS scenarios for new infections over time, in order to determine which scenarios are similar and which are not^3,4,31^. Since PHAS simulations provide only point estimates without confidence intervals, error measures like the Weighted Interval Score (WIS) are not directly applicable^3,31–33^. To address this limitation, we proceeded by relying on traditional error measures like Dynamic Time Warping (DTW), Euclidean distance, and Mean Absolute Percentage Error (MAPE) for assessing accuracy. However, these may overlook important epidemiological characteristics such as area under the curve (representing total disease burden over time), peak timings and growth/decline rates (representing outbreak dynamics) and thus reduce their effectiveness in scenario comparison, therefore we introduce a new error measure based on a set of specific attributes designed to capture the epidemiological similarity between simulated and observed case time series.

## Methods

### Data

**Fig. 1:**
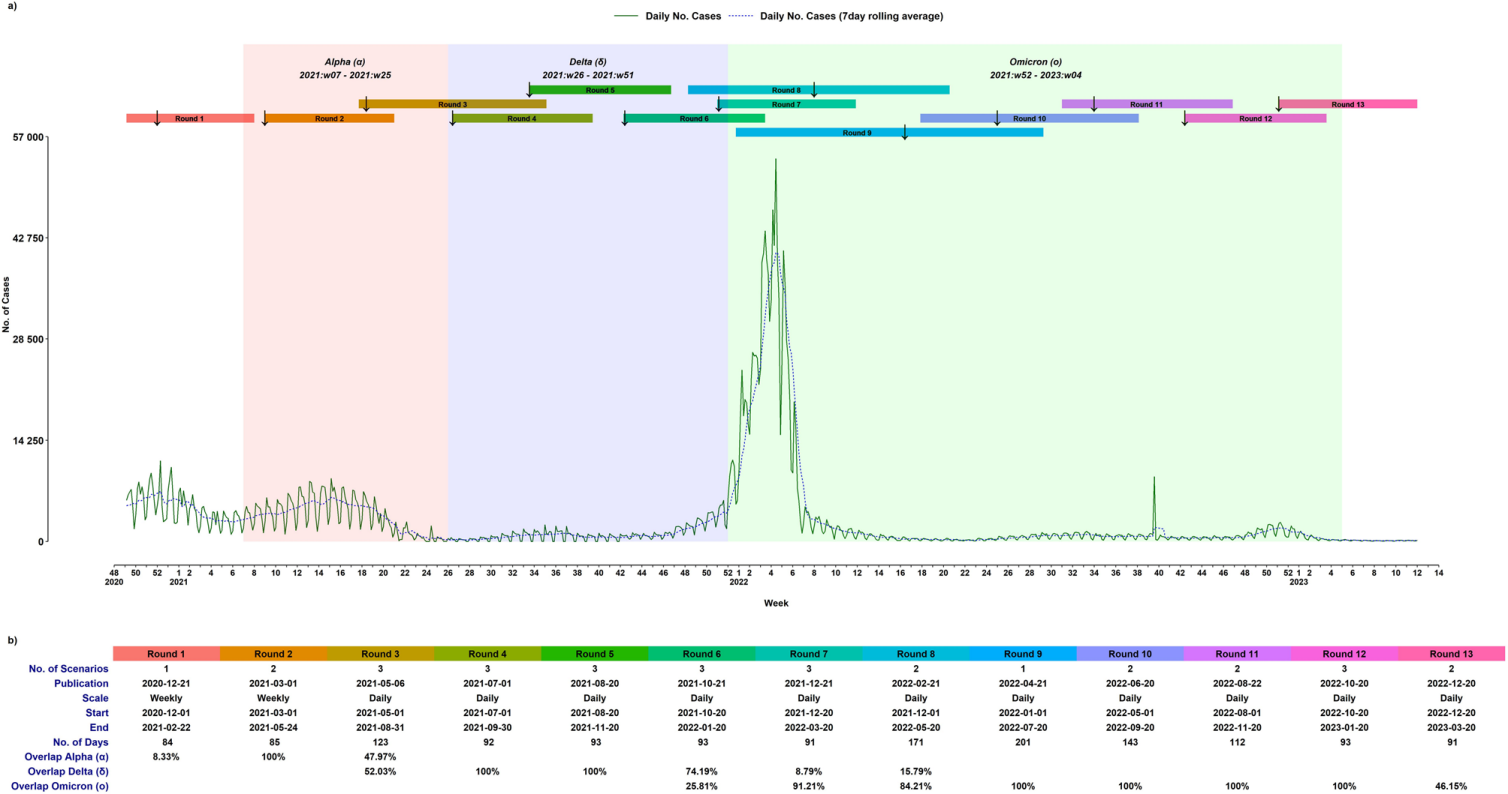
PHAS released thirteen rounds of simulations of new infections, from December 1, 2020, to March 20, 2023, with each round featuring one to three scenarios. (**a**) Each round is represented by a unique colour. The figure displays the raw daily national case counts (in green) and the smoothed 7-day rolling averages (SDCC in blue). Intervals for simulation rounds are highlighted as bars, publication dates are marked with downward arrows, and shaded areas indicate periods dominated by different variants. (**b**) The table provides details for each round, including the number of scenarios, publication date, reporting scale, period covered, duration in days, and the extent of overlap between the simulation period and the dominance of various variants.

**Fig. 2:**
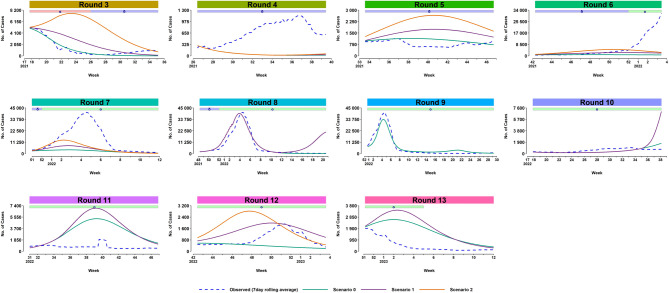
The smoothed 7-day rolling averages daily national (Riket) case count (dashed blue line) across different simulation rounds, along with the simulated case numbers for each specific scenario.

The PHAS assignment led to the creation of seventeen distinct simulation efforts aimed at continuously updating scenarios for the spread of the virus that causes COVID-19. These consist of thirteen primary and two interim simulation rounds exploring various scenarios for new infections, and additional two targeted simulations designed to support decision-making on vaccination strategies. Current analysis utilizes data from the thirteen primary simulation rounds in conjunction with updated daily COVID-19 case data retrieved from the PHAS website (www.folkhalsomyndigheten.se) as of August 18, 2023. In each simulation round scenarios were consistently labelled as Scenario 0, Scenario 1, and Scenario 2 associated with unique underlying assumptions and increased level of severity in disease spread. In text for simplicity, we will index each round (Rn) and scenario (Sn) by respective number (n) e.g., R3-S0 for Round 3 Scenario 0 or simply R3 if we refer to all simulation scenarios of round 3. Except for the first two rounds, all remaining eleven rounds provided daily number of simulated cases, and the total number of scenarios across all considered simulation rounds sum to twenty-seven, as not all rounds included three scenarios. The core simulations were conducted at the national level, while regional projections were derived by dividing the national simulations according to each region’s population relative to the total Swedish population. The baseline curve, the Smoothed Daily Case Count (SDCC), is derived from the daily-recorded count of observed cases, specifically presented in its smoothed 7-day rolling average form to eliminate the effect of periodic volatility and outliers^34^. Figure 1 displays the unprocessed daily national case count curve along with its smoothed 7-day rolling average (SDCC) in which observed sudden peak during week 39, 2022 is due to late reporting and retroactive registration for some regions. This figure highlights the intervals for the various simulation rounds, their publication dates, and periods dominated by different variants. Notably, not all simulations rounds covered the same number of scenarios, and some even overlapped in their timeframes. Certain rounds provided scenario estimates a longer period before their publication dates, these are confined to their respective training periods, in order to highlight if a recent peak before publication was modelled adequately by the specific scenarios or not. Figure 2 displays each rounds scenario estimates in addition to observed SDCC.

## Methods

### Dissimilarity measure

To effectively compare disease scenario projections of new infections with the SDCC curve of observed cases from an epidemiological perspective under the assumption of a single peak during the projection period, we assess several key attributes (Table 1), including A1-A2: the timing of the peak, A3: the Area Under the Curve (AUC), and A4-A5: the growth/decline rates of the scenario curves in relation to the SDCC curve. Evaluating the AUC, representing the total disease burden over time, provides critical insights into the scenarios overall performance and accuracy. Another crucial factor is the peak timing as it measures how accurately the scenario estimates the timing of disease surges. Furthermore, examining the growth/decline rate of the curve before and after the peak reveals additionally whether the scenario approximately captures the dynamics of disease spread, including phases of acceleration and deceleration.

**Table 1:** Considered attributes.

Attribute	Short Description	Long Description
A1	Peak timing Prior	Comparing if scenario curves peak timing is occurring at the same time or prior to the SDCC curves peak timing
A2	Peak timing Post	Comparing if scenario curves peak timing is occurring post the SDCC curves peak timing
A3	AUC	Comparing scenario curves AUC with the SDCC curves AUC
A4	Growth rate Prior	Comparing prior growth rate of the scenario curve (from its start to its peak) with SDCC curves prior growth rate
A5	Decline rate Post	Comparing post decline rate of the scenario curve (from its peak to its end value) with SDCC curves post decline rate

To assess the accuracy of these attributes, we quantify the error for each by calculating its respective Absolute Percentage Error (APE), defined as

1 $$\:APE(X,Y)\:=\left|\frac{X-Y}{X}\right|$$

where $\:X$ is the reference (true or actual) value and $\:Y$ is the comparator value. We modify the APE formulation to ensure that errors are well-defined and stable, even when the true denominator value is zero. The modified APE formulation denoted as $\:{APE}_{M}\left(X,Y\right)$, where the subscript M indicates the modification, is defined as

2 $$\:{APE}_{M}(X,Y)\:=\left|\frac{({X}^{\varvec{I}\left(X\ne\:0\right)}-\varvec{I}\left(X=0\right))-{(Y}^{\varvec{I}\left(Y\ne\:0\right)}-\varvec{I}\left(Y=0\right))}{{X}^{\varvec{I}\left(X\ne\:0\right)}}\right|$$

Where $\:\mathbf{I}(\bullet\:)$ denotes the indicator function. This transformation ensures that, when $\:X\ne\:0$ and $\:Y\ne\:0\:$then $\:{APE}_{M}\left(X,Y\right)\:$reduces to the standard APE. Adhering to the combinatorial convention that zero to the power of zero is one, if $\:X=0$ and $\:Y\ne\:0$, the entire magnitude of Y is set as an error which subsequently is capped at one (see below), and when $\:X\ne\:0$ and $\:Y=0$, the error is set to one. Finally, if $\:X=Y$, including when $\:X=0$ and $\:Y=0$, the $\:{APE}_{M}\left(X,Y\right)\:$is zero, as there is no difference between the X and Y values.

Among the attributes, A1 and A2 include an additional parameter, a window size $\:{\varDelta\:}^{prior}={\varDelta\:}^{post}\:=\varDelta\:\:=14$ days (chosen arbitrarily) centred around the peak location of the SDCC curve. For A1 and A2, we set the APE to zero if the peak of the scenario curve falls within the specified window, otherwise, the error is scaled relative to ∆ as follows

3 $$\begin{gathered} APE_{{A1}} = APE_{M} (\varDelta \:^{{prior}} ,t_{{SDCC}}^{p} - t_{{scenario}}^{p} )(1 - {\mathbf{I}}\left( {t_{{scenario}}^{p} \in \:\left[ {t_{{SDCC}}^{p} - \varDelta ^{{prior}} ,t_{{SDCC}}^{p} } \right]} \right)), \hfill \\ APE_{{A2}} = APE_{M} (\varDelta \:^{{post}} ,t_{{scenario}}^{p} - t_{{SDCC}}^{p} )(1 - {\mathbf{I}}\left( {t_{{scenario}}^{p} \in \:\:]t_{{SDCC}}^{p} ,t_{{SDCC}}^{p} + \Delta \:^{{post}} ]} \right)), \hfill \\ \end{gathered}$$

where $\:{t}_{SDCC}^{p}$ is the peak timing of the SDCC curve, and $\:{t}_{scenario}^{p}$ is the peak timing of the scenario curve.

For A3, we apply the trapezoidal rule to approximate the area under each curve (AUC), for which then APE is

4 $$\:{APE}_{A3}={APE}_{M}\left({AUC}_{SDCC},{AUC}_{Scenario}\right).\:$$

To calculate APE for A4 and A5, the respective linear growth/decline rates before and after the peaks are evaluated, if defined, to construct the APEs as

5 $$\begin{gathered} \:K_{{SDCC}}^{1} = \:\frac{{Y_{{SDCC}}^{p} - Y_{{SDCC}}^{s} }}{{t_{{SDCC}}^{p} - t^{s} }},\:K_{{SDCC}}^{2} = \:\frac{{Y_{{SDCC}}^{e} - Y_{{SDCC}}^{p} }}{{t^{e} - t_{{SDCC}}^{p} }},\\K_{{scenario}}^{1} = \frac{{Y_{{scenario}}^{p} - Y_{{scenario}}^{s} }}{{t_{{scenario}}^{p} - t^{s} }}, \:K_{{scenario}}^{2} = \:\frac{{Y_{{scenario}}^{e} - Y_{{scenario}}^{p} }}{{t^{e} - t_{{scenario}}^{p} }},\\APE_{{A4}} = APE_{M} \left( {K_{{SDCC}}^{1} ,K_{{scenario}}^{1} } \right), \hfill \\ APE_{{A5}} = APE_{M} \left( {K_{{SDCC}}^{2} ,K_{{scenario}}^{2} } \right), \hfill \\ \end{gathered}$$

where $\:\{{t}^{s},{Y}_{SDCC}^{s}\},$ {$\:{t}_{SDCC}^{p},{Y}_{SDCC}^{p}\}$, {$\:{t}^{e},{Y}_{SDCC}^{e}\}$ represent the start, peak, and end time points within the comparison window for the SDCC curve, along with their corresponding values. Similarly, $\:\{{t}^{s},{Y}_{scenario}^{s}\},$ {$\:{t}_{scenario}^{p},{Y}_{scenario}^{p}\}$, {$\:{t}^{e},{Y}_{scenario}^{e}\}$ denote the same points for the scenario curve.

Subsequently we cap each $\:{APE}_{{A}_{j}},\:j\in\:\{\text{1,2},\text{3,4},5\}$ at one to prevent extreme values. These are then combined using specific weights $\:{w}_{i}$ to form the asymmetric Similarity Error ($\:SEr$)

6 $$\:SEr=\frac{\sum\:_{i=1}^{5}{w}_{i}\mathbf{I}\left({APE}_{{A}_{i}}\:\text{i}\text{s}\:\text{d}\text{e}\text{f}\text{i}\text{n}\text{e}\text{d}\right)\text{m}\text{i}\text{n}(1,{APE}_{{A}_{i}})}{\sum\:_{i=1}^{5}{w}_{i}\mathbf{I}\left({APE}_{{A}_{i}}\:\text{i}\text{s}\:\text{d}\text{e}\text{f}\text{i}\text{n}\text{e}\text{d}\right)}.$$

that ranges between 0 and 1. $\:SEr\:$is never based on all the defined attributes, as A4 and A5 may not always be well-defined due to potential alignment issues (e.g., if peak timing coincides with either starting or end timing for each curve), and for each comparison either A1 (when $\:{t}_{scenario}^{p}\le\:{t}_{SDCC}^{p})$ or A2 (when $\:{t}_{scenario}^{p}>{t}_{SDCC}^{p}$) is present not both.

## Other measures

We also assess $\:SEr$ performance alongside other traditional key measures such as the Dynamic Time Warping algorithm (DTW), Euclidean distance and Mean Absolute Percentage Error (MAPE). The Euclidean distance calculates the square root of the sum of the squared distance between actual (X) and simulated values (Y) by

7 $$\:ED(X,Y)=\sqrt{\sum\:_{i=1}^{n}{({x}_{i}-{y}_{i})}^{2}}$$

for vectors $\:X=\{{x}_{1},\dots\:,{x}_{n}\}$ and $\:Y=\{{y}_{1},\dots\:,{y}_{n}\}$ while the asymmetric MAPE measures the average absolute percentage difference between actual and simulated values relative to the actual values by

8 $$\:MAPE\left(X,Y\right)=\frac{1}{n}\sum\:_{i=1}^{n}\left|\frac{{x}_{i}-{y}_{i}}{{x}_{i}}\right|.$$

DTW, more flexible, extends these approaches by finding an optimal alignment between series $\:X$ and $\:Y$, allowing for nonlinear comparisons by warping the time axis^35,36^. The minimal DTW distance and corresponding optimal warping path are computed using a dynamic programming algorithm. DTW computes a distance measure between $\:X$ and $\:Y$ by constructing a cost matrix $\:C\in\:{\mathbb{R}}^{nxn}$, where each element $\:C(i,j)$ represents the local cost of aligning elements $\:{x}_{i}$ and $\:{y}_{j}$ usually defined as $\:{C\left(i,j\right)=({x}_{i}-{y}_{j})}^{2}$^35,36^. Using $\:C$ an accumulated cost matrix $\:D\in\:{\mathbb{R}}^{nxn}$ is evaluated by

9 $$\:\:D\left(i,j\right)=C\left(i,j\right)+min\left\{\begin{array}{c}D\left(i-1,j-1\right)\\\:D\left(i-1,j\right)\\\:D\left(i,j-1\right)\end{array}\right.$$

with boundary conditions imposed on the first row and column. Once the accumulated cost matrix is computed, the overall DTW distance is given by $\:DTW\left(X,Y\right)=D\left(n,n\right)$. The optimal warping path is obtained by backtracking from $\:D\left(n,n\right)$ to $\:D\left(\text{1,1}\right)$ along the minimum-cost neighbours^35,36^.

## Optimal configuration and threshold

To select between a 3-attribute (A1–A3) and full 5-attribute (A1–A5) versions of $\:SEr$, we conduct a Receiver Operating Characteristic (ROC) analysis using three different weighting schemes where performance is evaluated using the AUC. Subsequently we choose the configuration that achieves the highest AUC^ROC^ and identify an optimal classification threshold, $\:\epsilon\:\in\:\left[\text{0,1}\right]$, which distinguishes “Similar” ($\:SEr\le\:\epsilon\:$) from “Not Similar” ($\:SEr>\epsilon\:$) sequences (Supplementary ROC analysis).

All data processing, calculations, and plotting in this paper are done using the statistical software R (version 4.4.3)^37^.

## Results

### Similarity assessment on National level

**Fig. 3:**
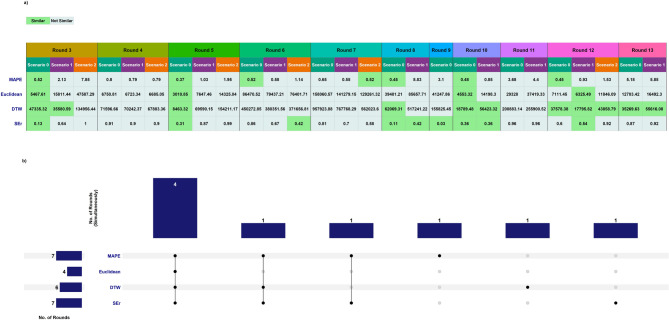
Similarity classification of the national (Riket) simulation rounds. (**a**) The four error measures classified based on thresholds determined through ROC analysis. The specific thresholds are as follows: $\:{\epsilon\:}_{SEr}=0.54$, $\:{\epsilon\:}_{DTW}=62069.31$, $\:{\epsilon\:}_{Euclidean}=6325.49$, $\:{\epsilon\:}_{MAPE}=0.52$. (**b**) Upset figure displaying how many distinct rounds each error measures or combination of error measures exhibited similarity simultaneously.

To avoid relying on arbitrary thresholds we visually assessed which scenario curves we deem to be similar (R3-S0, R5-S0, R8-S0, R9-S0, R10-S0, R12-S1) to the SDCC on a national level and followed up with ROC-analysis and different weighting schemes to select between a 3-attribute (A1–A3) and full 5-attribute (A1–A5) versions of $\:SEr$ by choosing the one that achieved highest AUC. The ROC analysis pointed towards the full 5-attribute $\:SEr$ (AUC^ROC^= 97%) with following weights $\:{w}_{1}=1,{w}_{2}=2,\:{w}_{3}=f({AUC}_{SDCC}$, $\:{AUC}_{Scenario}),{w}_{4}=2$ and $\:{w}_{5}=1$ estimating an optimal threshold of $\:{\epsilon\:}_{SEr}$ = 0.54 for classification (Supplementary ROC analysis). The function $\:f$ considers the scale of $\:{AUC}_{SDCC}$ and the over or underestimation of $\:{AUC}_{Scenario}$ by measuring the absolute difference to $\:{AUC}_{SDCC}$ and is defined as

10 $$\begin{gathered} \:f(AUC_{{SDCC}} ,\:AUC_{{Scenario}} ) = {\text{max}}\left( {1,\left\lfloor {log_{{10}} \left( {\left| {AUC_{{SDCC}} } \right|} \right)} \right\rfloor } \right) \hfill \\ *{\text{max}}\left( {1,\left\lfloor {log_{{10}} \left( {\left| {AUC_{{Scenario}} - AUC_{{SDCC}} } \right|} \right)} \right\rfloor } \right) \hfill \\ \end{gathered}$$

with $\left\lfloor x \right\rfloor = floor\left( x \right)$. Using the full 5-attribute $\:SEr$ and the estimated optimal threshold for classification, curves R6-S2, R8-S1, and R10-S1 were classified as similar, contradicting original classification, alongside the 6 curves that were visually identified as similar. Resulting in 9 out of 27 scenario comparisons (33%) were classified as similar, represented by 7 out of 11 simulation rounds (64%) with at least one scenario classified as similar (Fig. 3). In R4, R7, R11 and R13 no $\:SEr$-similar scenario was observed.

Repeating the ROC analysis with more traditional comparison error measures resulted in AUC^ROC^ values of 82% (DTW, $\:{\epsilon\:}_{DTW}=62069.31$), 79% (Euclidean, $\:{\epsilon\:}_{Euclidean}=6325.49$), and 77% (MAPE, $\:{\epsilon\:}_{MAPE}=0.52$) all of which are lower than $\:SEr$’s AUC^ROC^ (Supplementary ROC analysis). DTW classified 11 scenarios across 6 rounds (54%) as similar: R3-S0*, R3-S1, R5-S0*, R8-S0*, R10-S0*, R10-S1*, R12-S0, R12-S1, R12-S2, R13-S0, and R13-S1 (Fig. 3). Of these five were also $\:SEr$-similar (*-marked). The Euclidean distance classified 4 scenarios across 4 (36%) rounds: R3-S0*, R5-S0*, R10-S0*, and R12-S1*, all of which were $\:SEr$-similar as well. The MAPE classified 7 scenarios across 7 (64%) rounds: R3-S0*, R5-S0*, R6-S0, R7-S2, R8-S0*, R10-S0*, and R12-S0, of which four were also $\:SEr$ similar. Jointly R3-S0, R5-S0, and R10-S0 were classified as similar by all considered error measures. In R3, R5, R10, R12 either error measures identified at least one scenario to be classified as similar (Fig. 3).

Examining the $\:SEr$ decomposition (Table 2) reveals that scenarios classified as similar displayed consistent patterns of low feature-level errors that contributed to their reduced $\:SEr$ values. Specifically, the timing before peak (A1) frequently fell within the admissible window, resulting in an $\:{APE}_{A1}=0$, while other features also showed relatively low APEs. This alignment led to a lower weighted error sum in the $\:SEr$ numerator, bringing the overall value below the similarity threshold. In contrast, non-similar scenarios were characterized by high APEs across multiple features, often reaching 1, resulting in a substantially larger numerator and pushing the $\:SEr$ well above the threshold.

**Table 2:** Decomposition table of SEr for the National comparison.

*SEr*													
**Scenario**	$\:{\varvec{A}\varvec{P}\varvec{E}}_{\varvec{A}1}$	$\:{\varvec{A}\varvec{P}\varvec{E}}_{\varvec{A}2}$	$\:{\varvec{A}\varvec{P}\varvec{E}}_{\varvec{A}3}$	$\:{\varvec{A}\varvec{P}\varvec{E}}_{\varvec{A}4}$	$\:{\varvec{A}\varvec{P}\varvec{E}}_{\varvec{A}5}$	$\:{\varvec{w}}_{1}$	$\:{\varvec{w}}_{2}$	$\:{\varvec{w}}_{3}$	$\:{\varvec{w}}_{4}$	$\:{\varvec{w}}_{5}$	$\:\varvec{D}$	$\:\varvec{N}$	$\:\varvec{S}\varvec{E}\varvec{r}$
*SEr* similar scenarios													
R3-S0	0		0.132		0.2261	1		20		1	22	2.8661	0.13
R5-S0		0	0.2826	0.4659	1		2	16	2	1	21	6.4534	0.31
R6-S2	1		0.3696	0.8123		1		25	2		28	11.8646	0.42
R8-S0	0		0.1238	0.0491	0.0542	1		30	2	1	34	3.8664	0.11
R8-S1	0		0.4596	0.0491	0.5621	1		30	2	1	34	14.4483	0.42
R9-S0	0		0.0125	0.1509	0.1684	1		24	2	1	28	0.7702	0.03
R10-S0		1	0.2354	0.6914			2	16	2		20	7.1492	0.36
R10-S1		1	0.2	1			2	16	2		20	7.2	0.36
R12-S1	0		0.6459	0.0155	0.5237	1		16	2	1	20	10.8891	0.54
*SEr* non-similar scenarios													
R3-S1	0		0.6774		0.2206	1		25		1	27	17.1556	0.64
R3-S2		1	1		1		2	25		1	28	28	1
R4-S0	1		0.9012		0.8914	1		16		1	18	16.3106	0.91
R4-S1	1		0.8975		0.897	1		16		1	18	16.257	0.9
R4-S2	1		0.8921		0.9068	1		16		1	18	16.1804	0.9
R5-S1		1	0.8956	0.5132	1		2	16	2	1	21	18.356	0.87
R5-S2		0.9286	1	1	1		2	20	2	1	25	24.8572	0.99
R6-S0	1		0.8447	0.9918		1		25	2		28	24.1011	0.86
R6-S1	0.9286		0.6337	0.9318		1		25	2		28	18.6347	0.67
R7-S0	0		0.8236	0.9621	0.9328	1		36	2	1	40	32.5066	0.81
R7-S1	0		0.71	0.8003	0.8466	1		30	2	1	34	23.7472	0.7
R7-S2	0.0714		0.5998	0.5333	0.7426	1		30	2	1	34	19.8746	0.58
R11-S0	0		1	1	1	1		20	2	1	24	23	0.96
R11-S1	0		1	1	1	1		20	2	1	24	23	0.96
R12-S0	1		0.5493		0.9253	1		16		1	18	10.7141	0.6
R12-S2	0.7857		0.9635	1	0.2052	1		16	2	1	20	18.4069	0.92
R13-S0		0.3571	1	0.2155	0.616		2	20	2	1	25	21.7612	0.87
R13-S1		0.5714	1	0.4815	1		2	20	2	1	25	23.1058	0.92

D = Denominator, N = Numerator, SEr = N/D rounded to two decimal points

**Fig. 4:**
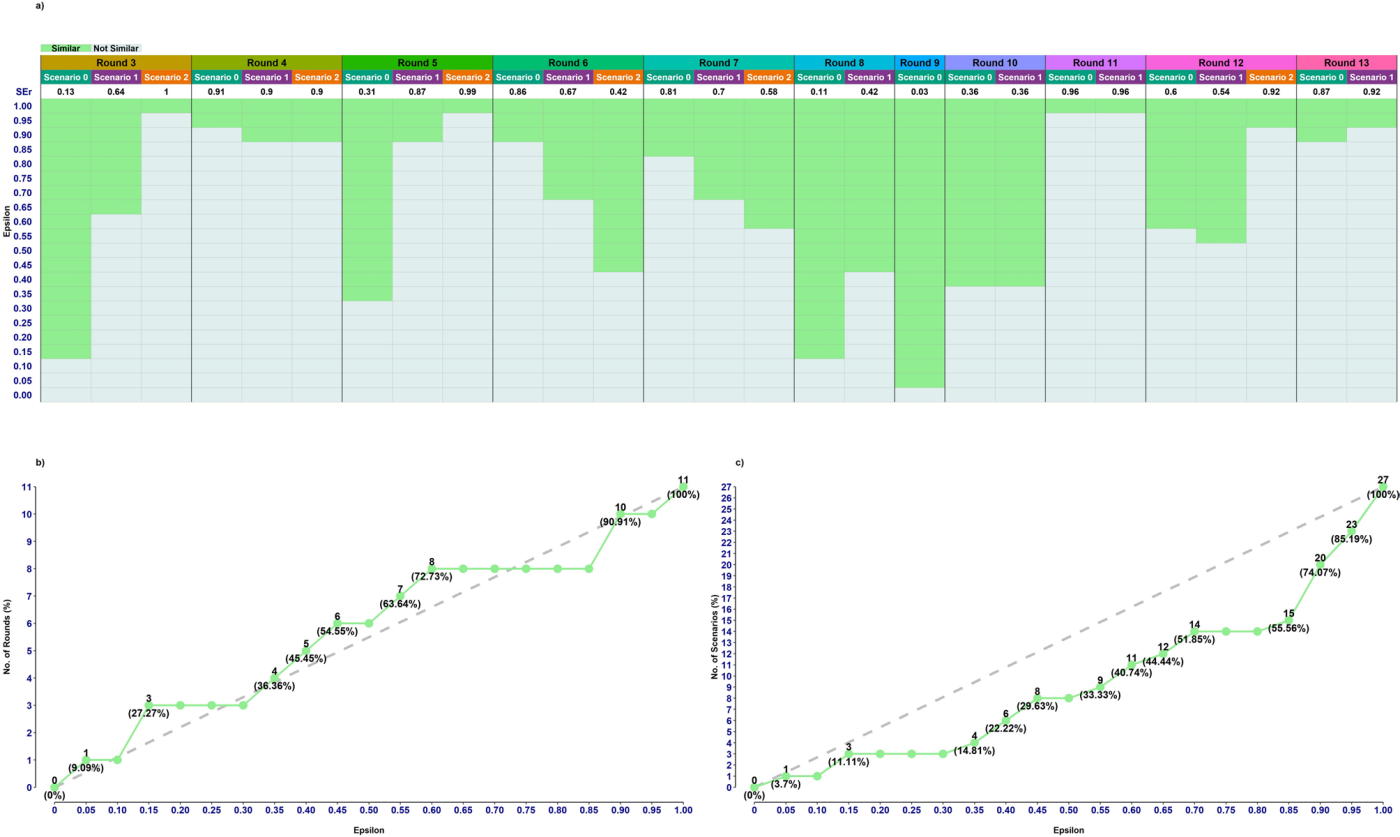
Classification performance of $\:SEr$. **a**) Over a range of $\:\epsilon\:$ values where each of the national (Riket) scenarios per round are classified and colour coded accordingly. **b**) Number of rounds and **c**) Number of scenarios classified as $\:SEr$ Similar over the range of $\:\epsilon\:$ values.

Complementing with a sensitivity analysis for $\:SEr$ based on a range of epsilon values from 0 to 1 in incremental steps of 0.05 manifest intriguing patterns (Fig. 4). The lower the threshold epsilon, the more scenarios are classified as non-similar by the $\:SEr$ metric. At low epsilon values ($\:0<\epsilon\:\le\:0.1$), only a negligible fraction of rounds (9%) are identified as $\:SEr$-Similar. For moderate epsilon values ($\:0.15\le\:\epsilon\:\le\:0.50$), the proportion of $\:SEr$-Similar rounds rise steadily, reaching 55% when $\:\epsilon\:$ goes towards the upper range. For higher epsilon values ($\:0.55\le\:\epsilon\:\le\:0.85$), $\:SEr$-Similar rounds reach 73%. At very high epsilon values ($\:\epsilon\:\ge\:0.90$), $\:SEr$-Similar rounds overwhelmingly take precedence. For the scenarios the increase in the number of $\:SEr$-similar is slower and at $\:\epsilon\:\:=\:0.55\:$about a third of the scenarios are classified as similar. This trend is broken at around $\:\epsilon\:\:=\:0.8$ after which the number of $\:SEr$-similar scenarios increase faster. The fact that the number of $\:SEr$-similar rounds follow a constant linear trend (dashed line in Fig. 4) suggests that the minimal $\:SEr$ within rounds are evenly distributed in the unit interval, whereas lower rate of increase among the scenarios (the curve falls below the dashed line) implies that $\:SEr$-values for the scenarios are unevenly distributed and biased towards higher $\:SEr$-values.

### Similarity assessment on regional level

**Fig. 5:**
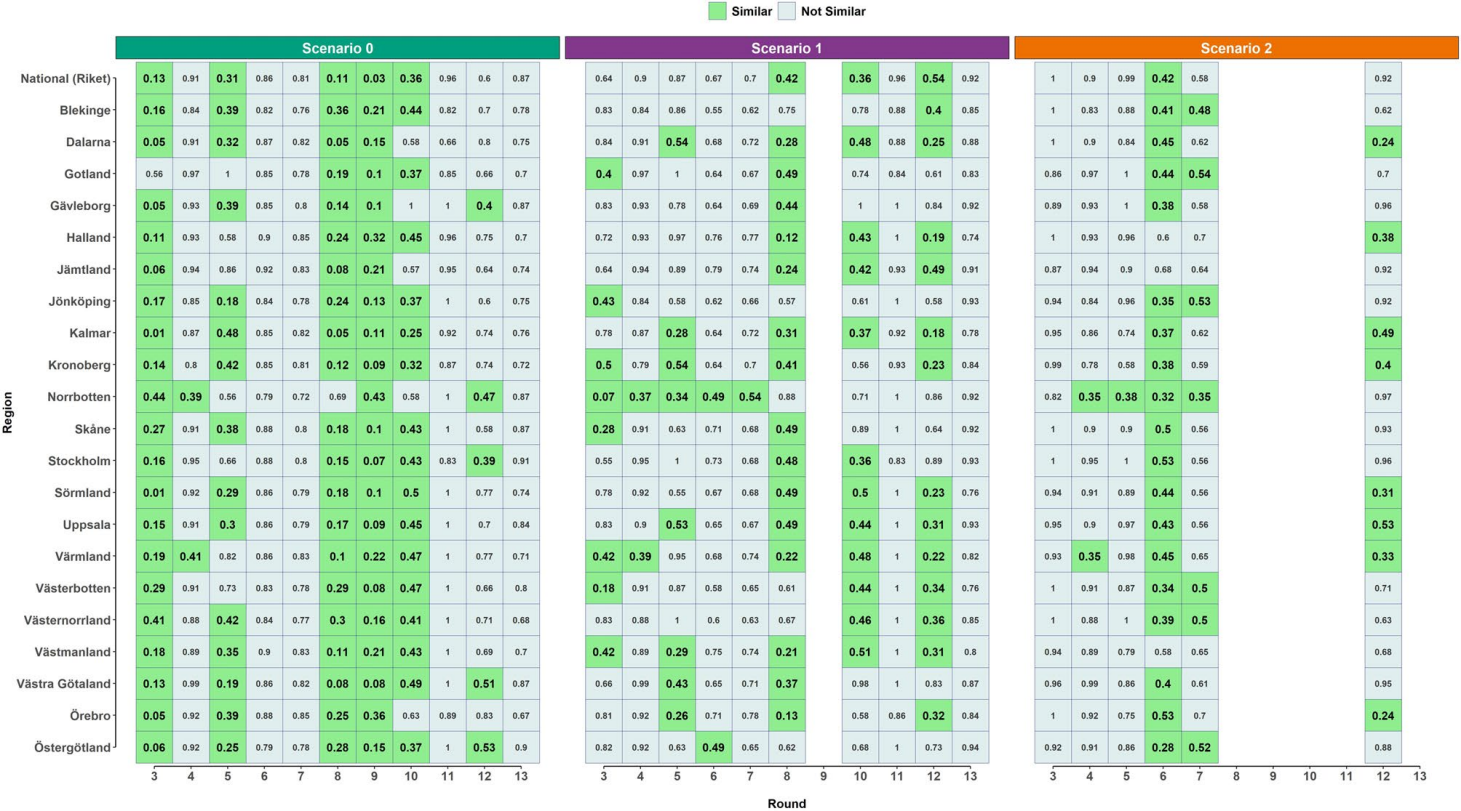
The $\:SEr$ categorization of all regions and scenarios for all simulations rounds and considered regions. Based on the configuration of $\:SEr$ determined by the national ROC analysis (Riket, representing the national level). Respective $\:SEr$-value is displayed in each tile and subsequently colour coded according to classification.

Assessments of the various regions are also presented in Fig. 5 under same configuration of $\:SEr$ as in the national comparison. Results show variability in the similarity percentages across different regions and simulation rounds (Supplementary Fig. 3). Regions *Halland* and *Jämtland* had the lowest number of $\:SEr$ similarity rounds, with 5 (46%) rounds, whereas regions *Blekinge*, *Västernorrland*, and *Östergötland* had the highest, with 8 (73%) rounds. The regions *Skåne*, *Stockholm*, and *Västra Götaland* are the most populous regions in Sweden. For *Skåne*, 8 scenarios across 6 (55%) rounds were $\:SEr$ similar (R3-S0, R3-S1, R5-S0, R6-S2, R8-S0, R8-S1, R9-S0, R10-S0). Similarly, for *Stockholm*, 8 scenarios across 6 (55%) rounds were $\:SEr$ similar (R3-S0, R6-S2, R8-S0, R8-S1, R9-S0, R10-S0, R10-S1, R12-S0). For *Västra Götaland*, 9 scenarios across 7 (64%) rounds were $\:SEr$ similar (R3-S0, R5-S0, R5-S1, R6-S2, R8-S0, R8-S1, R9-S0, R10-S0, R12-S0).

## Discussion

This study evaluates the accuracy of various COVID-19 simulation scenarios developed by the Public Health Agency of Sweden (PHAS) at both national and regional levels utilizing both traditional error measures as well as introducing a new asymmetric measure. We applied traditional error measures, including DTW, Euclidean distance, and MAPE, though each has limitations in capturing essential aspects of epidemic spread and are better suited for evaluating mathematical similarity or model fit. For instance, MAPE and Euclidean distance do not account for the cumulative scale of disease spread and primarily focus on pointwise differences and may miss structural differences, such as shifted peaks or diverging slopes, while DTW may fall short in representing differences in rates of increase or decrease and obscure meaningful epidemiological discrepancies. Moreover, traditional error measures often lack interpretability for epidemiological evaluation, reducing their effectiveness in comparing scenarios^35,36^.

Attempting to address these gaps, we developed a new, interpretable error measure $\:SEr$ designed to capture relevant epidemiological attributes, such as AUC, peak timing, and pre- and post-peak growth/decline rates. Using $\:SEr$ with threshold $\:{\epsilon\:}_{SEr}$ = 0.54, 7 (64%) out of 11 national comparison rounds had at least one scenario classified as similar to the SDCC, including five S0 (R3, R5, R8, R9, R10), three S1 (R8, R10, R12), and one S2 (R6). This includes all 6 scenarios that were visually identified as similar five S0 (R3, R5, R8, R9, R10), and one S1 (R12). $\:SEr$ decomposition (Table 2) shows that similar scenarios had low APEs, resulting in $\:SEr$ values below the similarity threshold. Notably, R3-S0, R8, R9-S0, and R12-S1 exhibited synchronized peaks relative to the SDCC, while R5-S0, though peaking after the SDCC, remained within the admissible range. In contrast, non-similar scenarios had high APEs, leading to elevated $\:SEr$ values, even in cases where peak timing appeared synchronized with the SDCC.

A key strength of $\:SEr$ lies in its multi-dimensional assessment, integrating diverse trajectory characteristics such as timing, magnitude, and shape condensed into a single, interpretable score. This comprehensive approach enables $\:SEr$ to detect discrepancies that traditional single-metric error measures—such as MAPE, Euclidean distance, or DTW—may overlook. Another important advantage of $\:SEr$ is its dynamic weight assignment, which allows modelers to emphasize specific attributes based on the objectives of the analysis. This flexibility makes $\:SEr$ highly adaptable: by adjusting the weights $\:{w}_{i}$, one can prioritize the accuracy of more critical features. For example, when optimizing a model for early pandemic detection, the $\:SEr$ framework allows for assigning greater weight to attribute A4, a distinct advantage over traditional metrics. Despite its strengths and valuable insights, $\:SEr$ has limitations. The sensitivity analysis demonstrates strong performance, particularly at extreme epsilon values, where the model aligns closely with expectations. While $\:SEr$ showcases robust classification abilities, fine-tuning is needed to better handle intermediate epsilon values. Its weighting flexibility allows for customization but introduces subjectivity. Additionally, capping extreme APE values improves stability but may mask some modelling issues. Future refinements could include adjusting attribute weights, different window sizes ($\:{\varDelta\:}^{prior}$and $\:{\varDelta\:}^{post})$, and exploring different thresholds ($\:\epsilon\:$) determination methods without the need of initial visual assessment. Furthermore, the linear approximations of the growth and decline phases implemented in $\:SEr$ are practical for ensuring comparability and provide a crude but interpretable estimate of factual trends. However, they may underrepresent the non-linear dynamics that often characterize critical phases of an outbreak, particularly during rapid acceleration or deceleration phases. Additionally, although AUC reflects the overall epidemic burden, it may obscure important variations in peak intensity—two scenarios might have comparable total cases yet differ substantially in their peak healthcare demands. Explicitly including peak magnitude would increase $\:SEr$’s sensitivity to these critical differences, thereby improving its value for public health decision-making and preparedness planning. Addressing multiple peaks and developing a symmetric version of $\:SEr$ would also enhance its robustness.

In conclusion, PHAS has provided long-term simulation rounds that aligned with observed trends according to the proposed $\:SEr$, facilitating proactive decision-making during the pandemic. However, effective disease modelling remains challenging^29,33^ due to evolving non-pharmaceutical interventions (NPIs), new virus variants, and reporting adjustments to name a few. As an example, focusing on R3 and R4 in Figs. 1 and 2, we note that R4 was published shortly after the period dominated by the Alpha variant. While the preparation and evaluation of R4 was carefully monitored, the rapid emergence and dominance of the Delta variant was impossible to predict given the knowledge then available. As a result, the assumptions that underpinned R4 did not fully align with the evolving reality, requiring a prompt reassessment, in particular peak timing and overall case burden was off for the scenarios of the round as evident visually and by the $\:SEr$ decomposition (Table 2). To swiftly respond to the changing epidemic trends, assumptions were readapted and R5 was released within just one month, in contrast to the two-month intervals between other simulation rounds. Scenarios generated during phases of relatively stable transmission patterns, without significant epidemiological shifts, were more likely to align with observed data. Consequently, effectively addressing the complexities of epidemic modelling necessitates continuous reassessment of foundational assumptions in each simulation round to capture the dynamic nature of the disease^3,31^. Continuous improvement of error measures, like our proposed $\:SEr$, which emphasizes epidemiological assessment by incorporating key features like timing, magnitude, and shape based on dynamic weight assignment can significantly enhance the evaluation of both retrospective and prospective models. By identifying trajectory components that are challenging to predict accurately via its decomposition, $\:SEr$ offers actionable insights. These insights can guide future modeling efforts by encouraging the inclusion of uncertainty bounds around critical features and the exploration of alternative weighting schemes to prioritize specific attributes according to the analysis objectives. This may aid the development of more diverse scenario assumptions, enabling a better representation of the full spectrum of plausible epidemic trajectories.

## Electronic supplementary material

Below is the link to the electronic supplementary material.

Supplementary Material 1

## Data Availability

The datasets analysed and source codes used to produce the findings of this study are available from the corresponding author upon reasonable request.
