# Supplementary material for: Comparison between simulated scenarios and Swedish COVID-19 cases throughout the pandemic
Source: Sci Rep. 2025 Jul 2;15:23653. doi: 10.1038/s41598-025-08682-z (PMC12222681; doi:10.1038/s41598-025-08682-z)

Similarity Error, Weights and Optimal Cut-off Threshold.

To apply SEr and assess the similarity between a candidate curve and a SDCC curve, we need to determine appropriate weights and a classification threshold (ε). We began by identifying a set of candidate curves that we visually assessed to be similar for the national comparison. These include R3-S0, R5-S0, R8-S0, R9-S0, R10-S0, and R12-S1. We examined a 3-attribute (A1-A3) and the full 5-attribute (A1-A5) version of SEr with three different weighting systems. We examined the following weighting schemes:

Supplementary Table 1: Weights Schemes for different attributes

Attribute	Uniform Weights	Alternative Weights 1	Alternative Weights 2
A1	1	1	1
A2	1	2	2
A3	1	1	$f(AUC_{SDCC}, AUC_{Scenario})$
A4	1	2	2
A5	1	1	1

The function f adjusts the weight of A3 by considering the scale of AUC_{SDCC} and the over or underestimation of $AUC_{Scenario}$ since as by design associated APE is capped and does not take values above one. The function f is defined as:

$$f(AUC_{SDCC}, AUC_{Scenario}) = \max(1, \lfloor log_{10}(|AUC_{SDCC}|)\rfloor) * \max(1, \lfloor log_{10}(|AUC_{Scenario} - AUC_{SDCC}|)\rfloor) \quad (1)$$

here $\lfloor x \rfloor = floor(x)$. Subsequently we constructed a Receiver Operating Characteristic (ROC) curve for each configuration by considering all simulation rounds and scenario curves. The optimal threshold is determined by first selecting the configuration of attributes and weight scheme that achieves the highest Area Under the ROC Curve (AUC^{ROC}). Next, the ideal true-positive and false-positive rates that minimize the distance between the ROC curve and the top-left corner are determined, subsequently the threshold that corresponds to these rates serves as the optimal threshold. Supplementary Table 2 shows AUC and associated 95% Confidence Interval (CI) for various metrics and identified optimal threshold and associated diagnostic metrics accompanying it. Supplementary Figure 2 displays the various ROC curves. In the alternative weight schemes the penalty for A2 (penalizing post-peak locations) and A4 (penalizing gradients prior to the peak) is doubled based on the rationale that effective disease management and resource allocation are more adversely affected by these discrepancies. For A2, if the simulated peak occurs after the actual peak, it can result in understaffing and inadequate resource deployment during the critical period when the disease is at its highest intensity. Timely intervention is crucial; therefore, post-peak inaccuracies have a significant negative impact on management strategies. For A4, if the simulated disease increase does not align

with the observed data's gradient, it indicates that the model is failing to accurately predict the rate of disease spread. This misalignment can lead to inappropriate responses and resource distribution, exacerbating the situation.

Supplementary Table 2: AUC^ROC and 95% confidence interval (CI) for various ROC curves and diagnostic measures associated with identified optimal threshold

Metric	Attributes	Weight	AUC^{ROC}	Lower CI	Upper CI	Threshold	TN[a]	TP[b]	FN[c]	FP[d]	Sensitivity	Specificity	NPV[e]	PPV[f]
SEr	A1-A3	Uniform Weights	0.9286	0.7991	1	0.32	20	5	1	1	0.8333	0.9524	0.9524	0.8333
		Alt. Weights 1	0.9048	0.7308	1	0.32	20	5	1	1	0.8333	0.9524	0.9524	0.8333
		Alt. Weights 2	0.9524	0.8663	1	0.32	20	5	1	1	0.8333	0.9524	0.9524	0.8333
	A1-A5	Uniform Weights	0.9325	0.8359	1	0.64	16	6	0	5	1	0.7619	1	0.5455
		Alt. Weights 1	0.9286	0.8222	1	0.37	19	5	1	2	0.8333	0.9048	0.95	0.7143
		Alt. Weights 2	0.9722	0.9172	1	0.54	18	6	0	3	1	0.8571	1	0.6667
DTW			0.8254	0.6246	1	62069.31	15	5	1	6	0.8333	0.7143	0.9375	0.4545
Euclidean			0.7937	0.5282	1	6325.49	21	4	2	0	0.6667	1	0.913	1
MAPE			0.7738	0.5219	1	0.52	18	4	2	3	0.6667	0.8571	0.9	0.5714

a: True Negative; b: True Positive; c: False Negative; d: False Positive

e: Negative Predictive Value; f: Positive Predictive Value

Supplementary Figure 1:

The smoothed 7-day rolling averages daily case count (dashed blue lines) across different simulation rounds, along with the simulated case numbers for each specific scenario at national level and for each region.

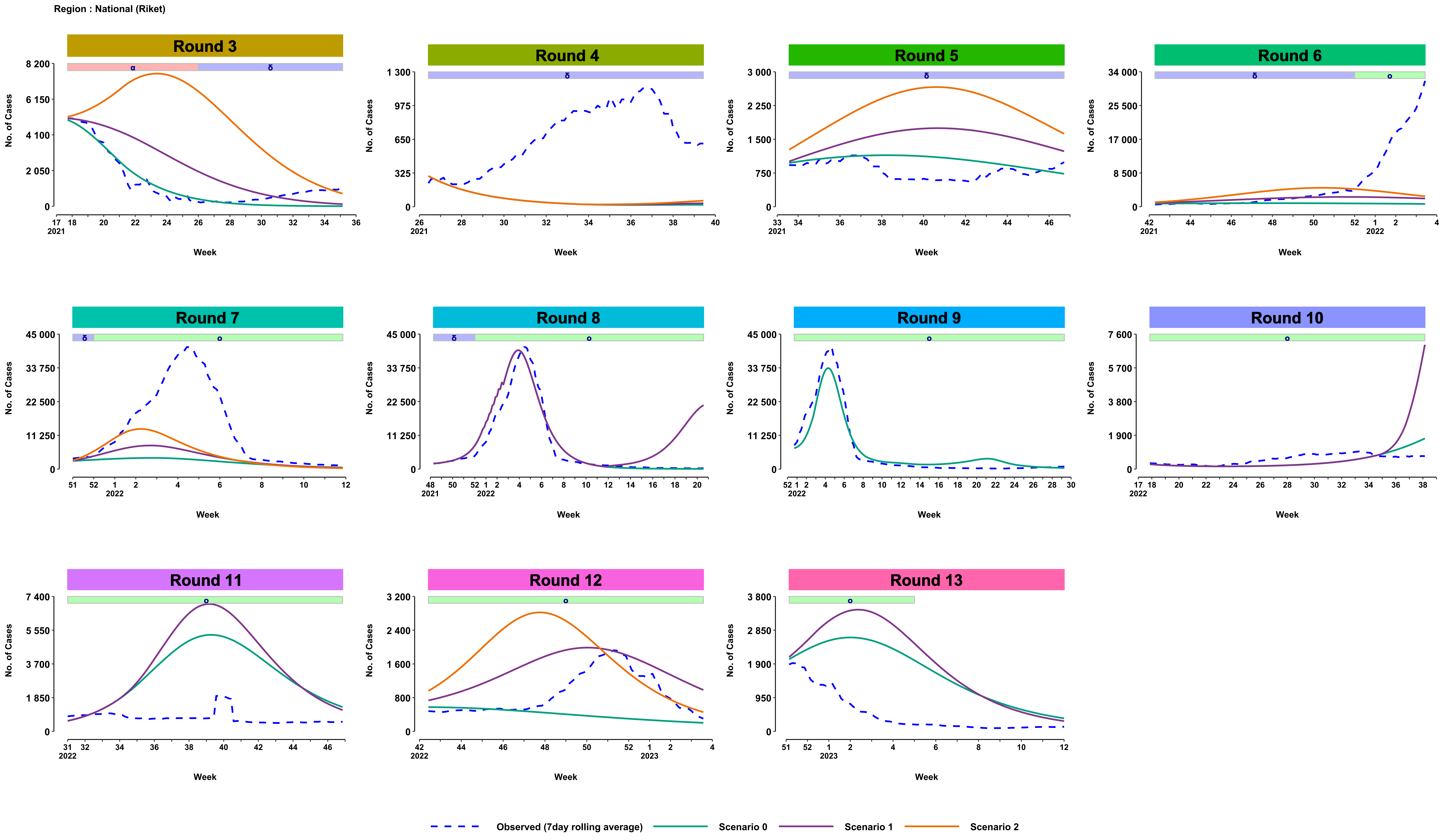
Region : National (Riket)
Round 3
Round 4
Round 5
Round 6
Round 7
Round 8
Round 9
Round 10
Round 11
Round 12
Round 13
α
δ
o
No. of Cases
Week
2021
2022
2023
Observed (7day rolling average)
Scenario 0
Scenario 1
Scenario 2

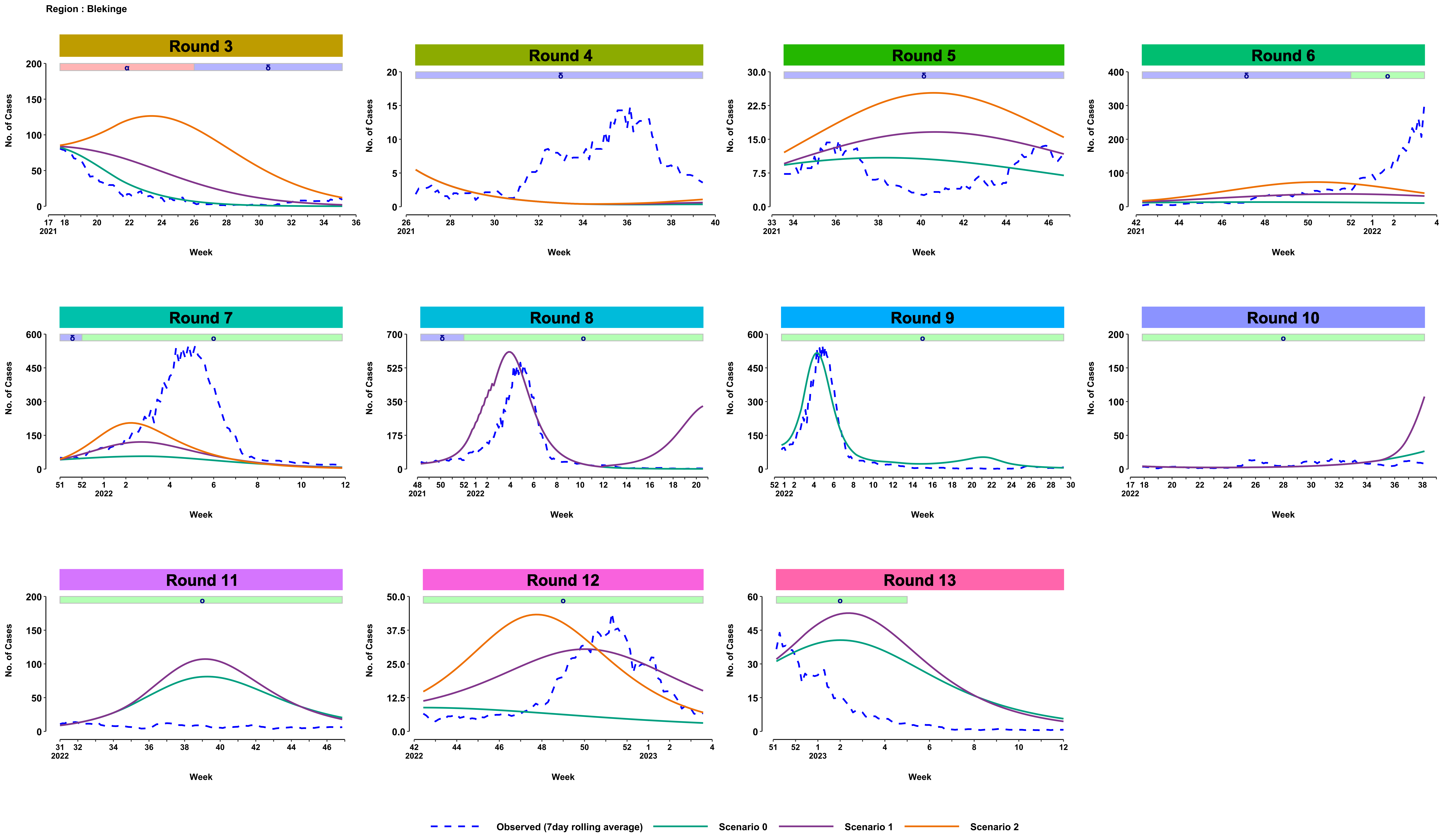

Region : Blekinge
Round 3
Round 4
Round 5
Round 6
Round 7
Round 8
Round 9
Round 10
Round 11
Round 12
Round 13
α
δ
o
No. of Cases
Week
2021
2022
2023
Observed (7day rolling average)
Scenario 0
Scenario 1
Scenario 2

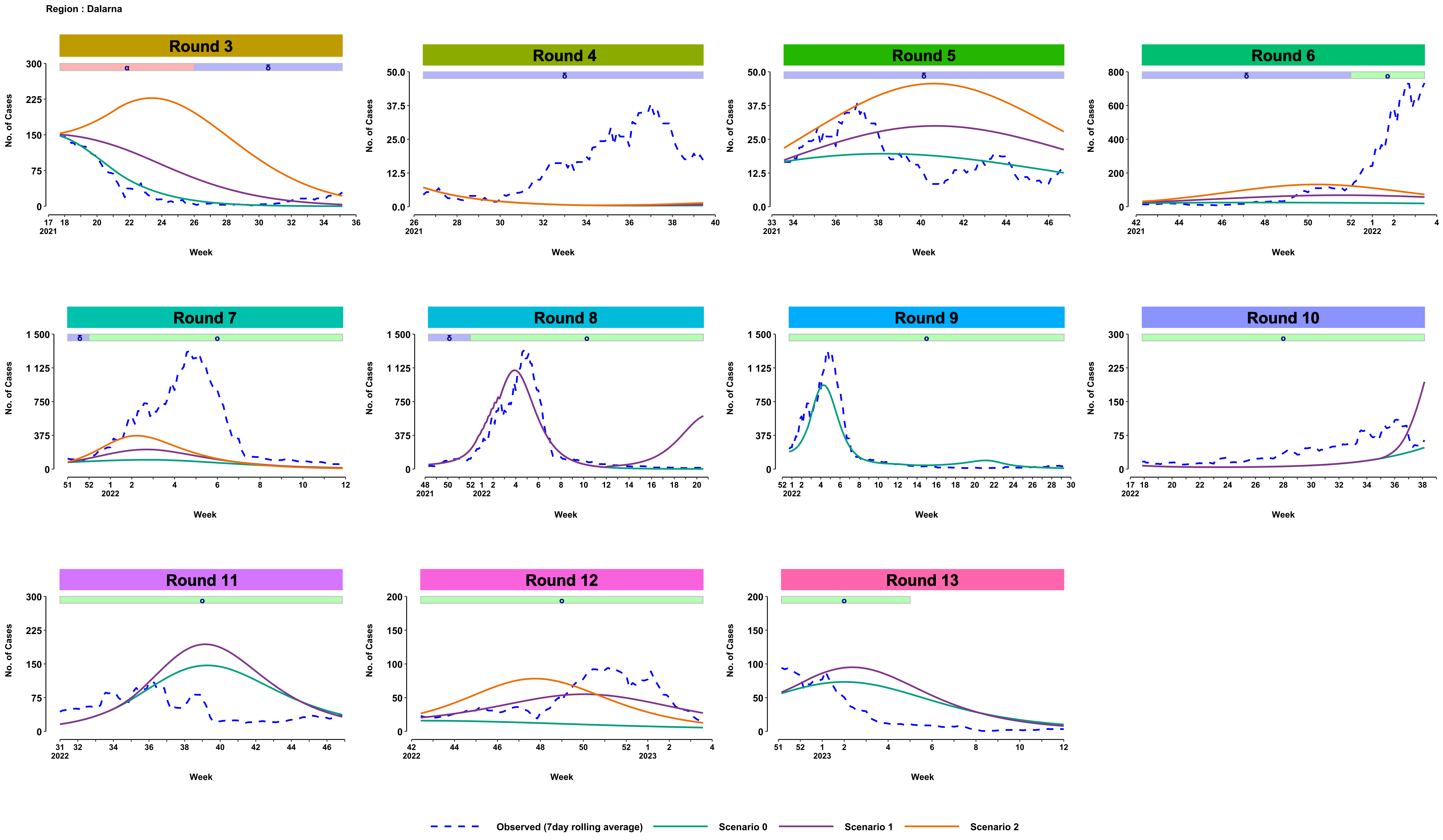
Region : Dalarna
Round 3
Round 4
Round 5
Round 6
Round 7
Round 8
Round 9
Round 10
Round 11
Round 12
Round 13
No. of Cases
Week
Observed (7day rolling average)
Scenario 0
Scenario 1
Scenario 2

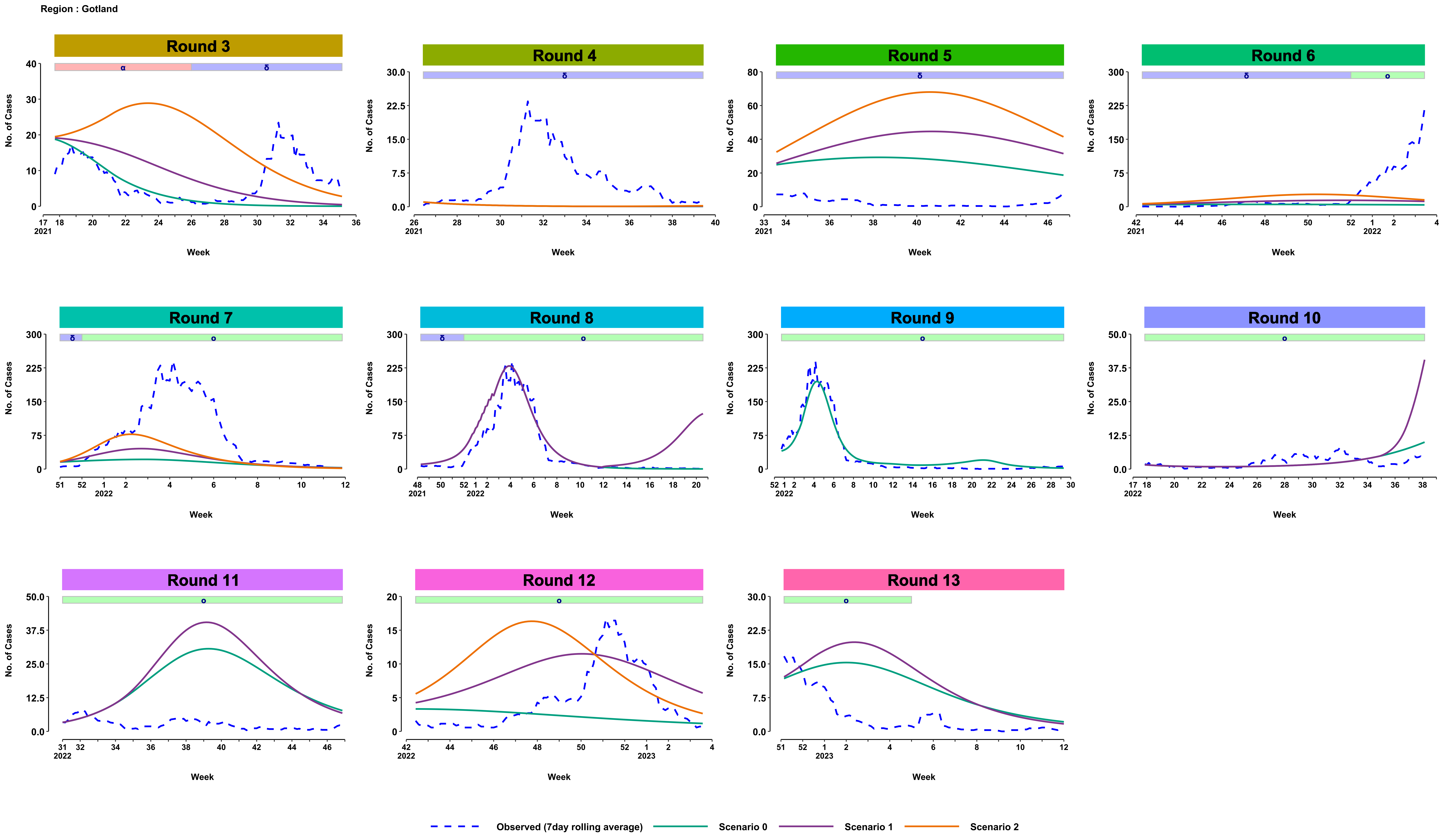
Region : Gotland
Round 3
Round 4
Round 5
Round 6
Round 7
Round 8
Round 9
Round 10
Round 11
Round 12
Round 13
No. of Cases
Week
Observed (7day rolling average)
Scenario 0
Scenario 1
Scenario 2

Region : Gävleborg

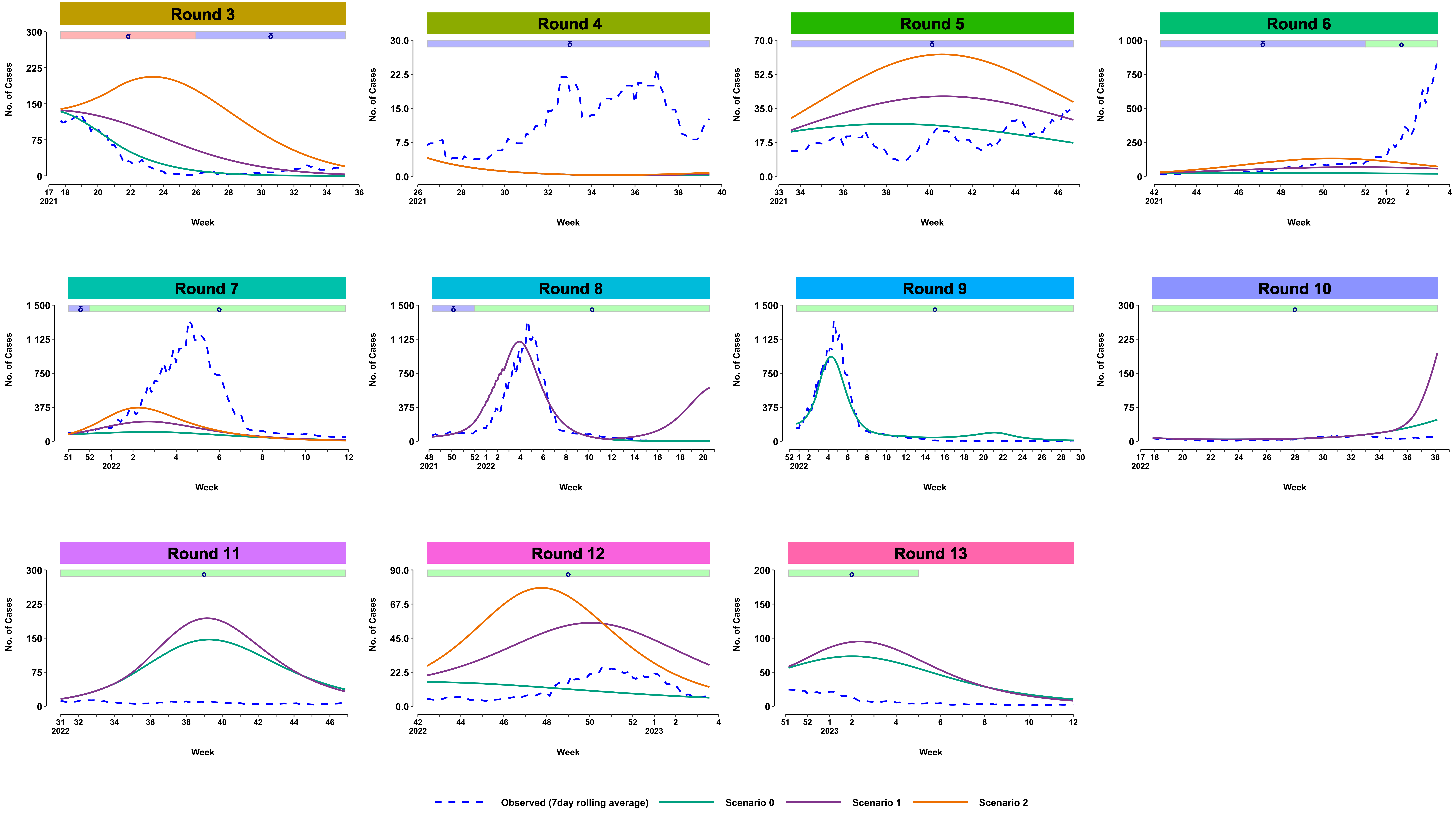

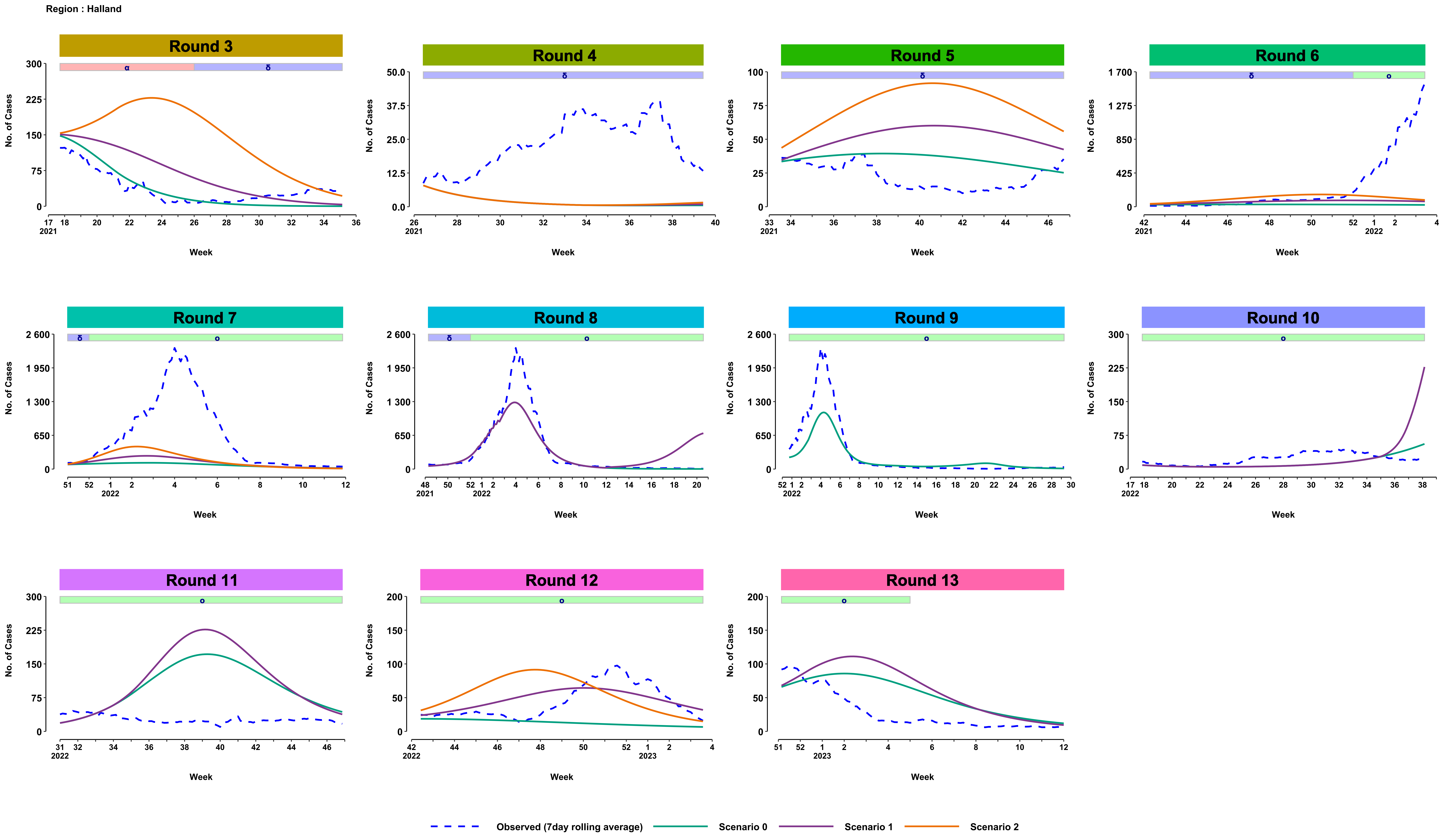
Region : Halland
Round 3
α
δ
No. of Cases
0
75
150
225
300
Week
17
2021
18
20
22
24
26
28
30
32
34
36
Round 4
δ
No. of Cases
0.0
12.5
25.0
37.5
50.0
Week
26
2021
28
30
32
34
36
38
40
Round 5
δ
No. of Cases
0
25
50
75
100
Week
33
2021
34
36
38
40
42
44
46
Round 6
δ
o
No. of Cases
0
425
850
1 275
1 700
Week
42
2021
44
46
48
50
52
1
2022
2
4
Round 7
δ
o
No. of Cases
0
650
1 300
1 950
2 600
Week
51
52
1
2022
2
4
6
8
10
12
Round 8
δ
o
No. of Cases
0
650
1 300
1 950
2 600
Week
48
2021
50
52
1
2022
2
4
6
8
10
12
14
16
18
20
Round 9
o
No. of Cases
0
650
1 300
1 950
2 600
Week
52
1
2022
2
4
6
8
10
12
14
16
18
20
22
24
26
28
30
Round 10
o
No. of Cases
0
75
150
225
300
Week
17
2022
18
20
22
24
26
28
30
32
34
36
38
Round 11
o
No. of Cases
0
75
150
225
300
Week
31
2022
32
34
36
38
40
42
44
46
Round 12
o
No. of Cases
0
50
100
150
200
Week
42
2022
44
46
48
50
52
1
2023
2
4
Round 13
o
No. of Cases
0
50
100
150
200
Week
51
52
1
2023
2
4
6
8
10
12
Observed (7day rolling average)
Scenario 0
Scenario 1
Scenario 2

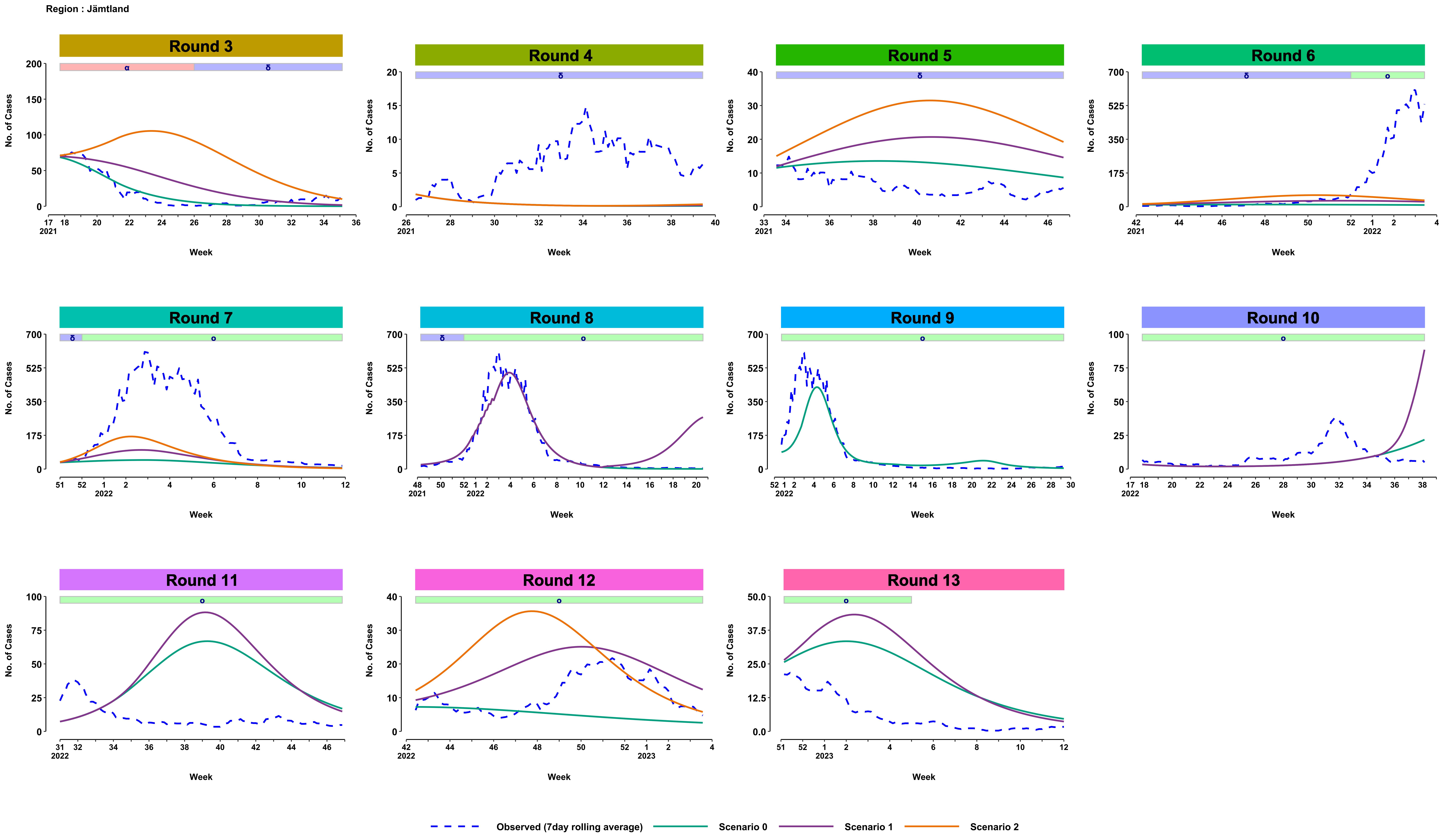
Region : Jämtland
Round 3
Round 4
Round 5
Round 6
Round 7
Round 8
Round 9
Round 10
Round 11
Round 12
Round 13
α
δ
o
No. of Cases
Week
2021
2022
2023
Observed (7day rolling average)
Scenario 0
Scenario 1
Scenario 2

Region : Jönköping

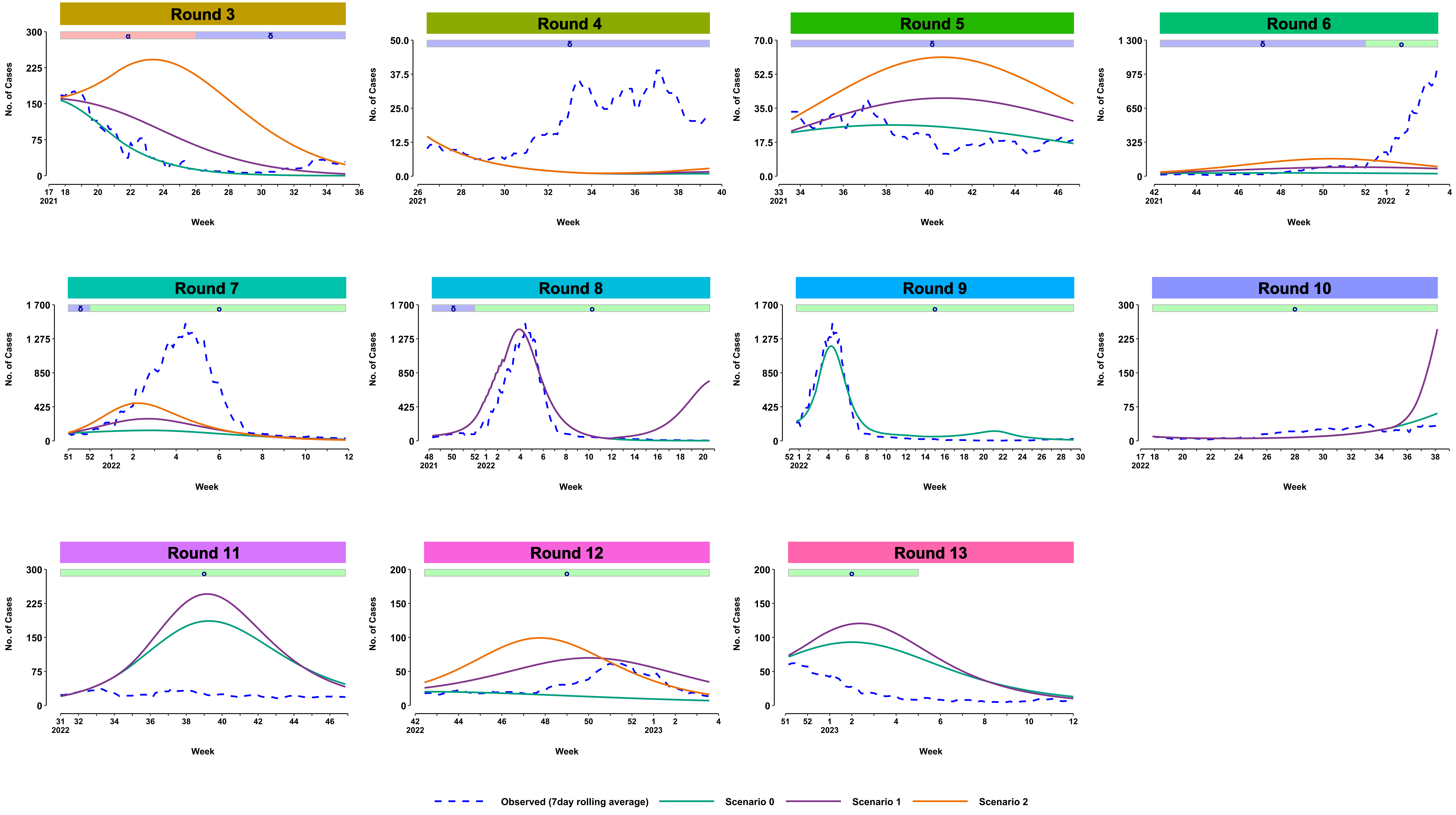

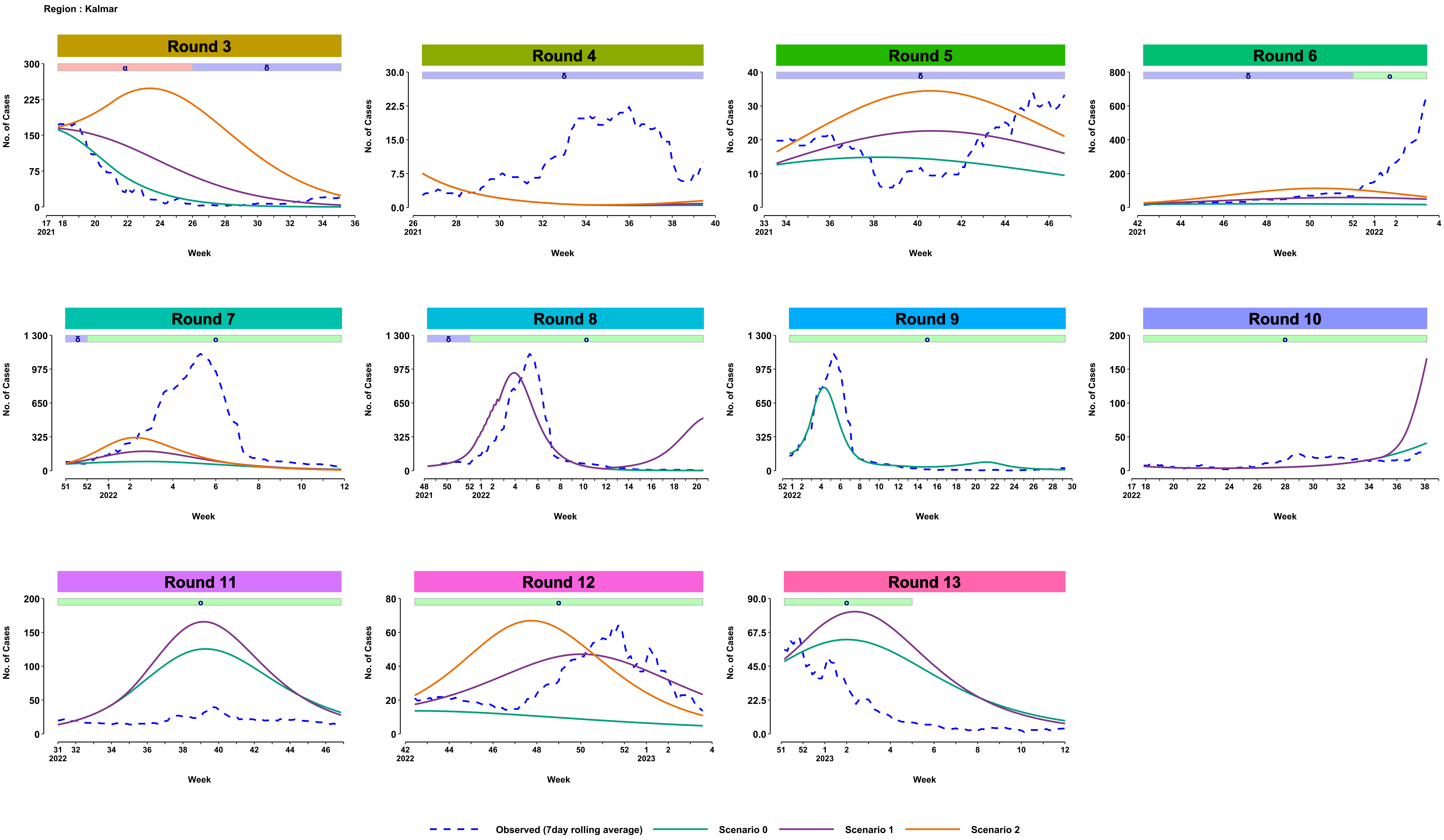
Region : Kalmar
Round 3
Round 4
Round 5
Round 6
Round 7
Round 8
Round 9
Round 10
Round 11
Round 12
Round 13
α
δ
o
No. of Cases
Week
2021
2022
2023
Observed (7day rolling average)
Scenario 0
Scenario 1
Scenario 2

Region : Kronoberg

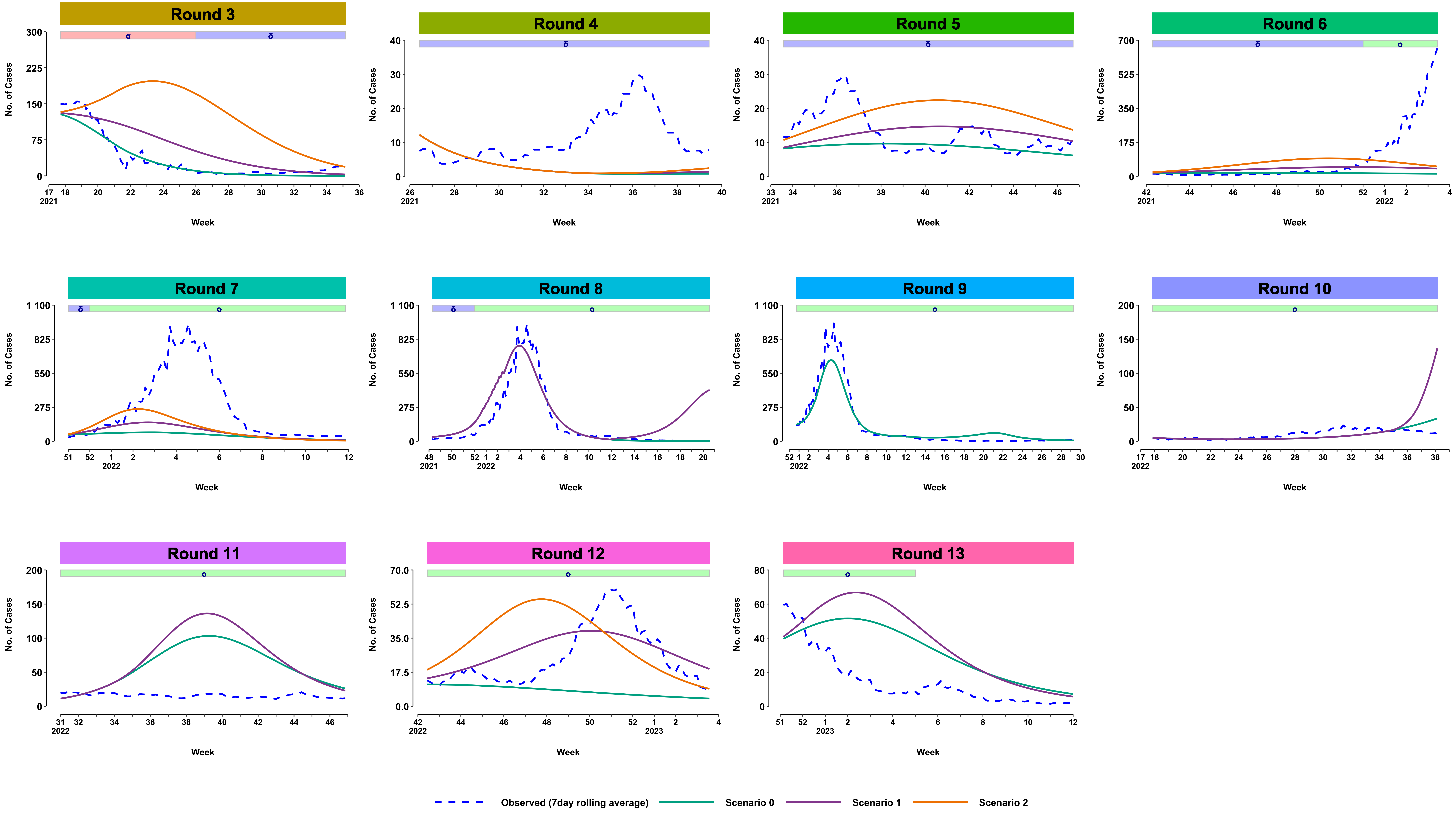

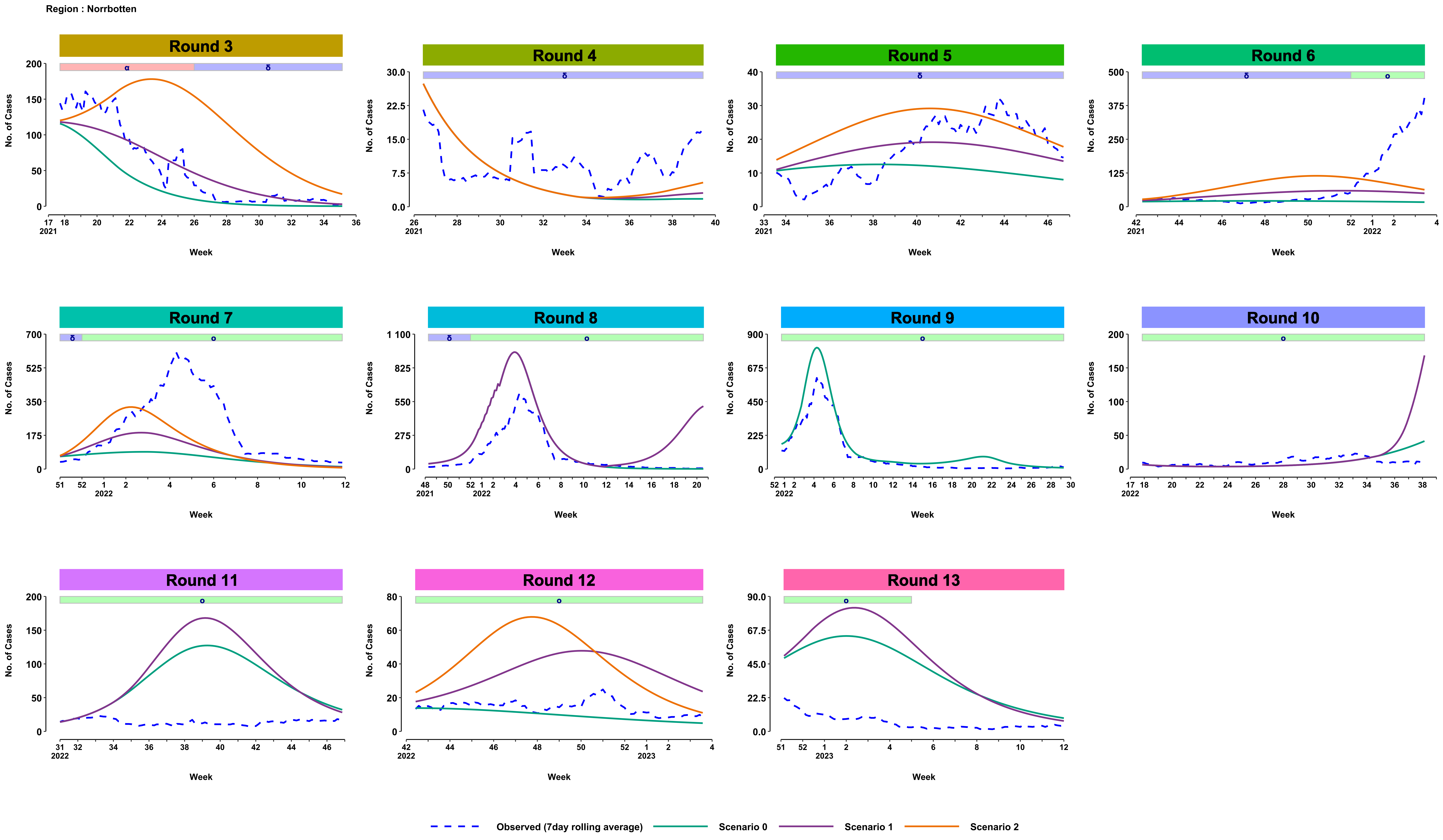
Region : Norrbotten
Round 3
Round 4
Round 5
Round 6
Round 7
Round 8
Round 9
Round 10
Round 11
Round 12
Round 13
α
δ
o
No. of Cases
Week
Observed (7day rolling average)
Scenario 0
Scenario 1
Scenario 2

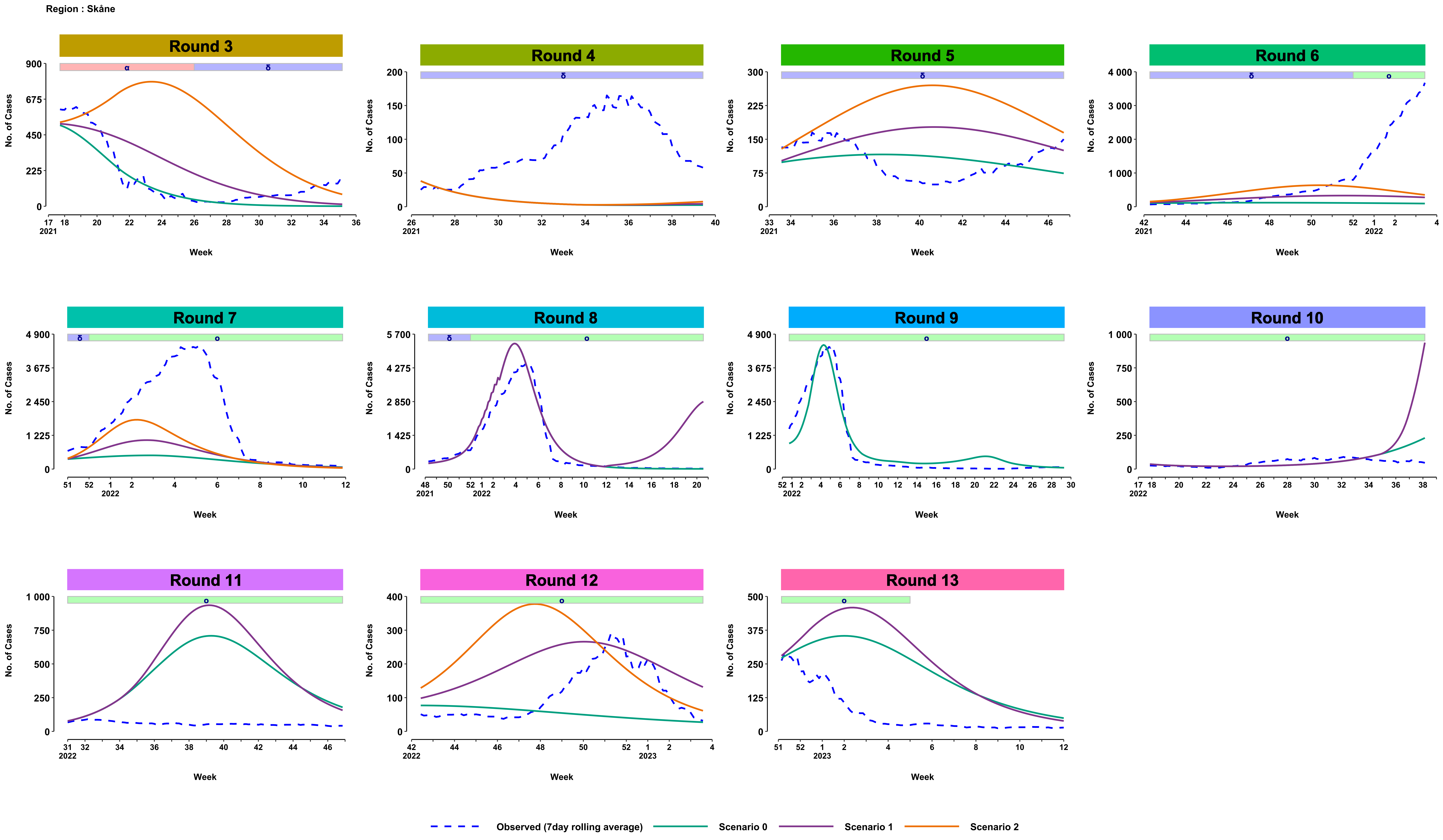
Region : Skåne
Round 3
Round 4
Round 5
Round 6
Round 7
Round 8
Round 9
Round 10
Round 11
Round 12
Round 13
α
δ
o
No. of Cases
Week
2021
2022
2023
Observed (7day rolling average)
Scenario 0
Scenario 1
Scenario 2

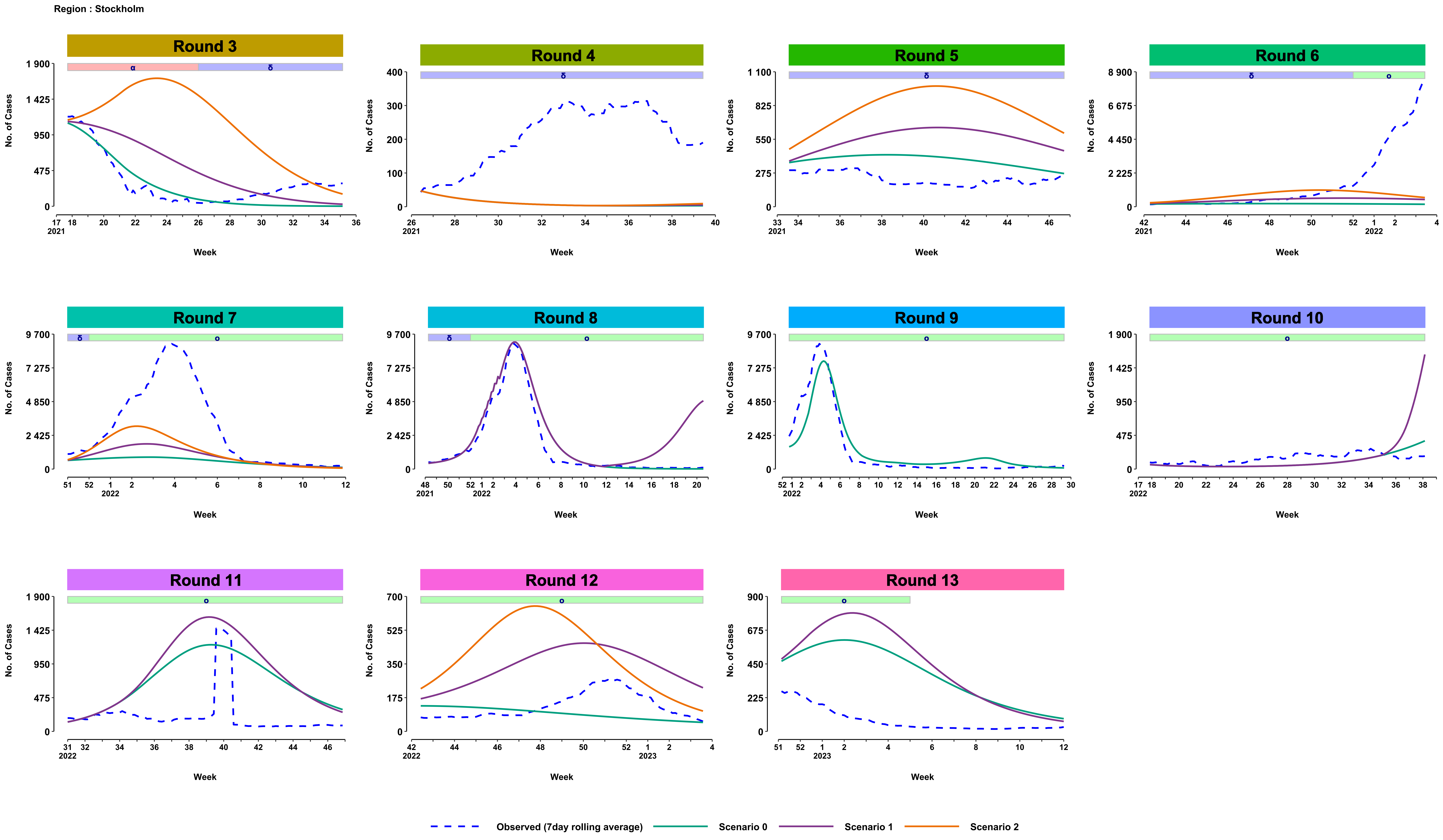

Region : Stockholm
Round 3
Round 4
Round 5
Round 6
Round 7
Round 8
Round 9
Round 10
Round 11
Round 12
Round 13
No. of Cases
Week
Observed (7day rolling average)
Scenario 0
Scenario 1
Scenario 2

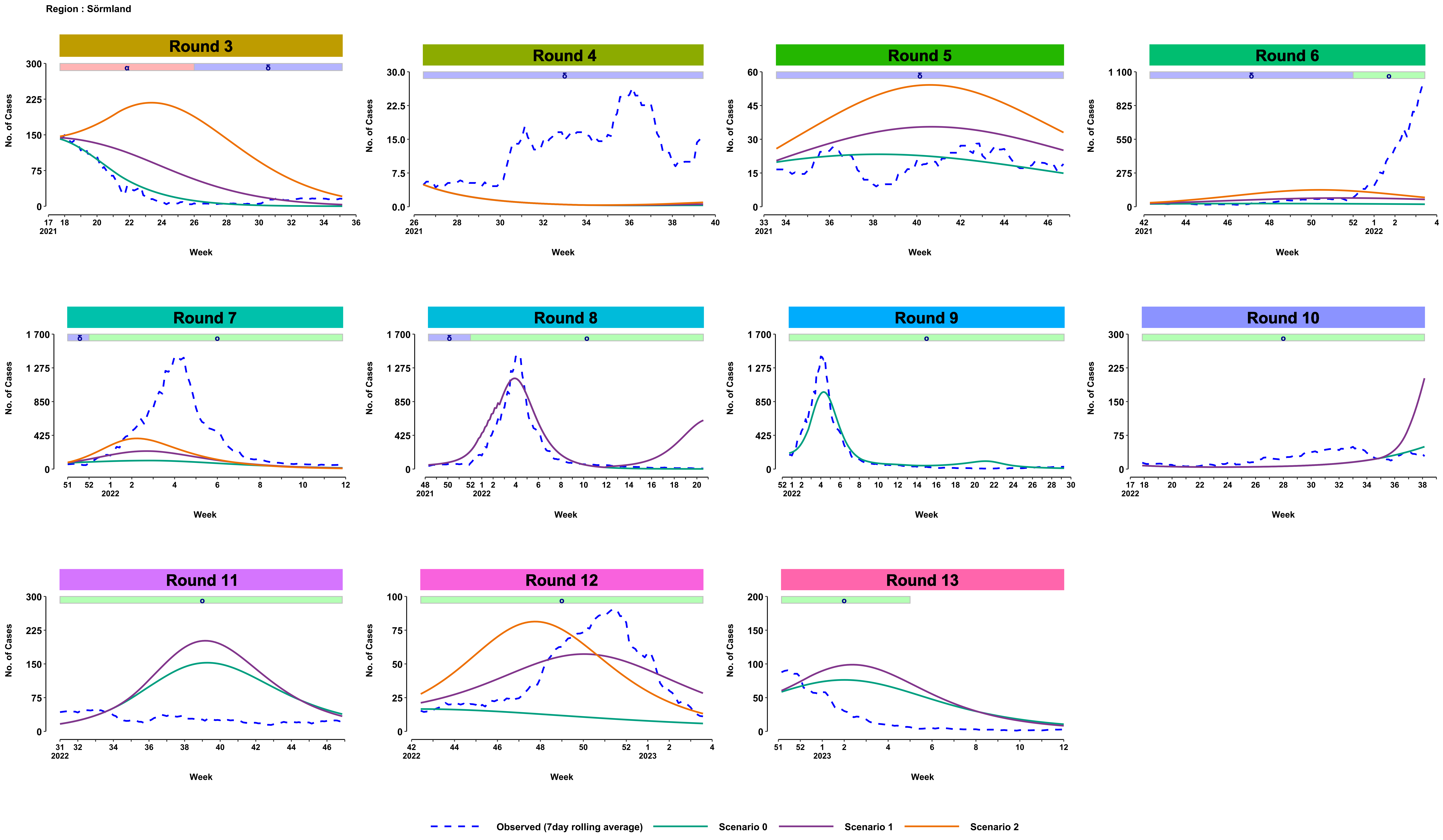
Region : Sörmland
Round 3
Round 4
Round 5
Round 6
Round 7
Round 8
Round 9
Round 10
Round 11
Round 12
Round 13
α
δ
o
No. of Cases
Week
2021
2022
2023
Observed (7day rolling average)
Scenario 0
Scenario 1
Scenario 2

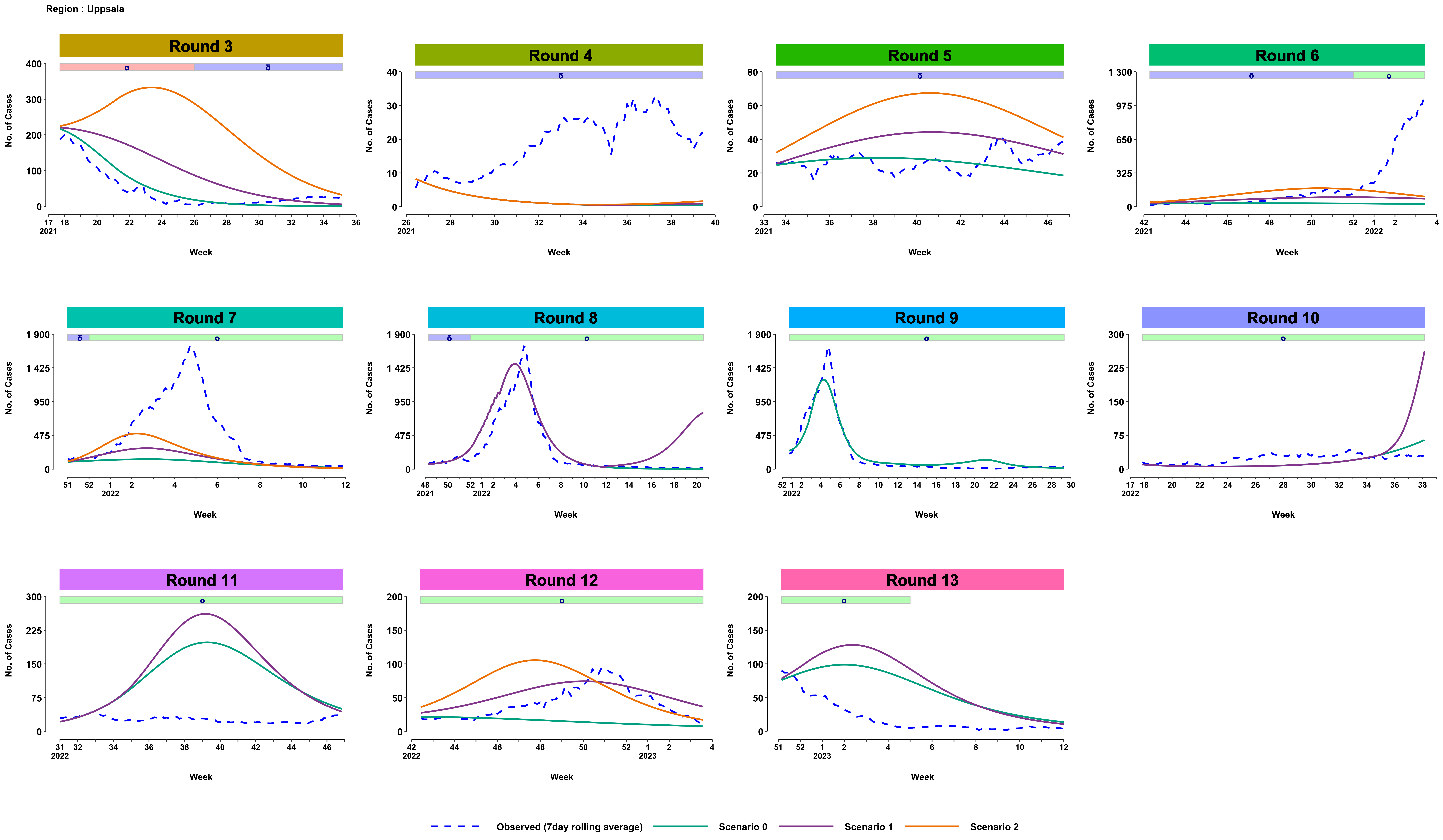

Region : Uppsala
Round 3
α
δ
No. of Cases
Week
Round 4
δ
Round 5
δ
Round 6
δ
o
Round 7
δ
o
Round 8
δ
o
Round 9
o
Round 10
o
Round 11
o
Round 12
o
Round 13
o
Observed (7day rolling average)
Scenario 0
Scenario 1
Scenario 2

Region : Värmland

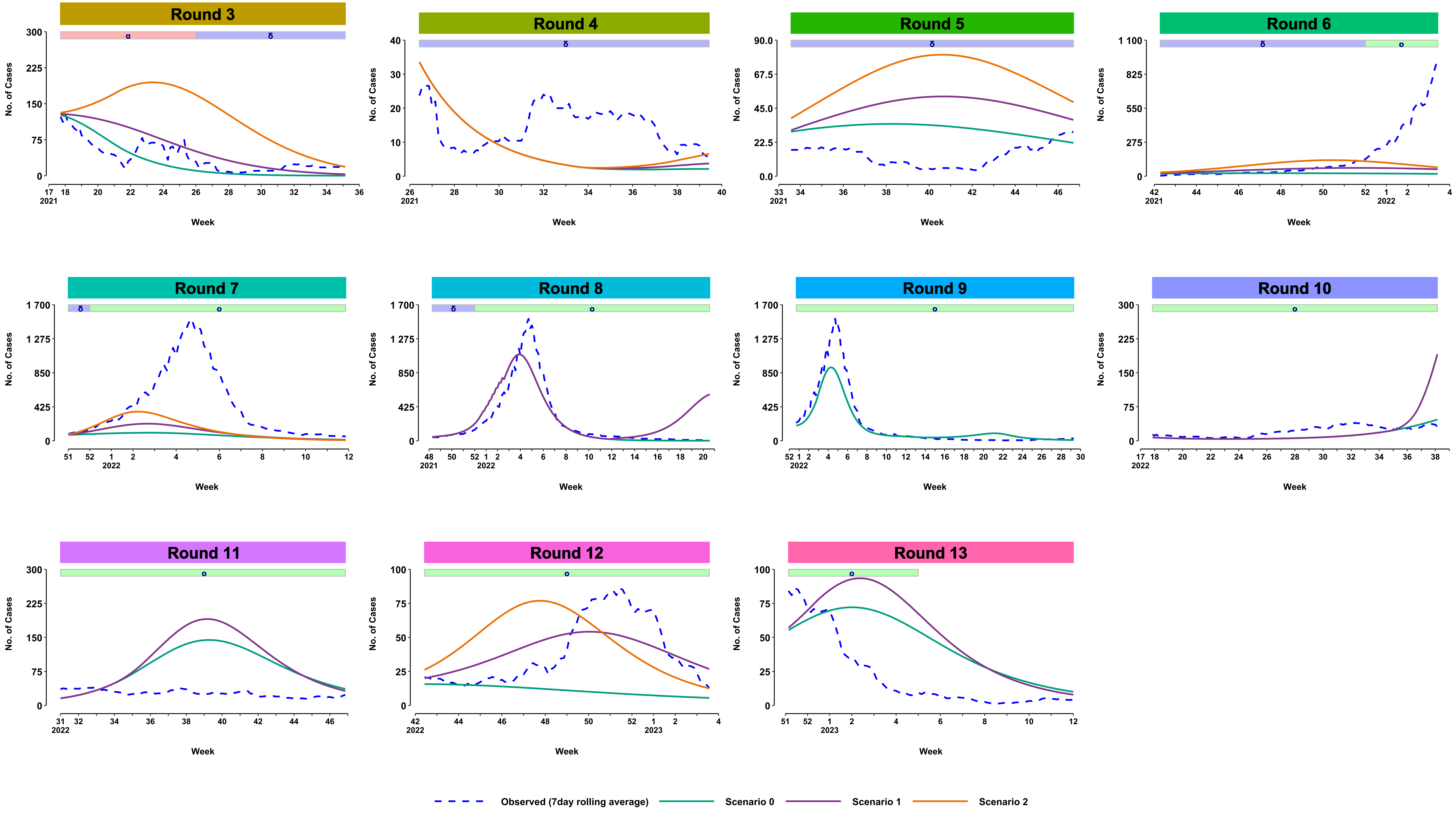

Region : Västerbotten

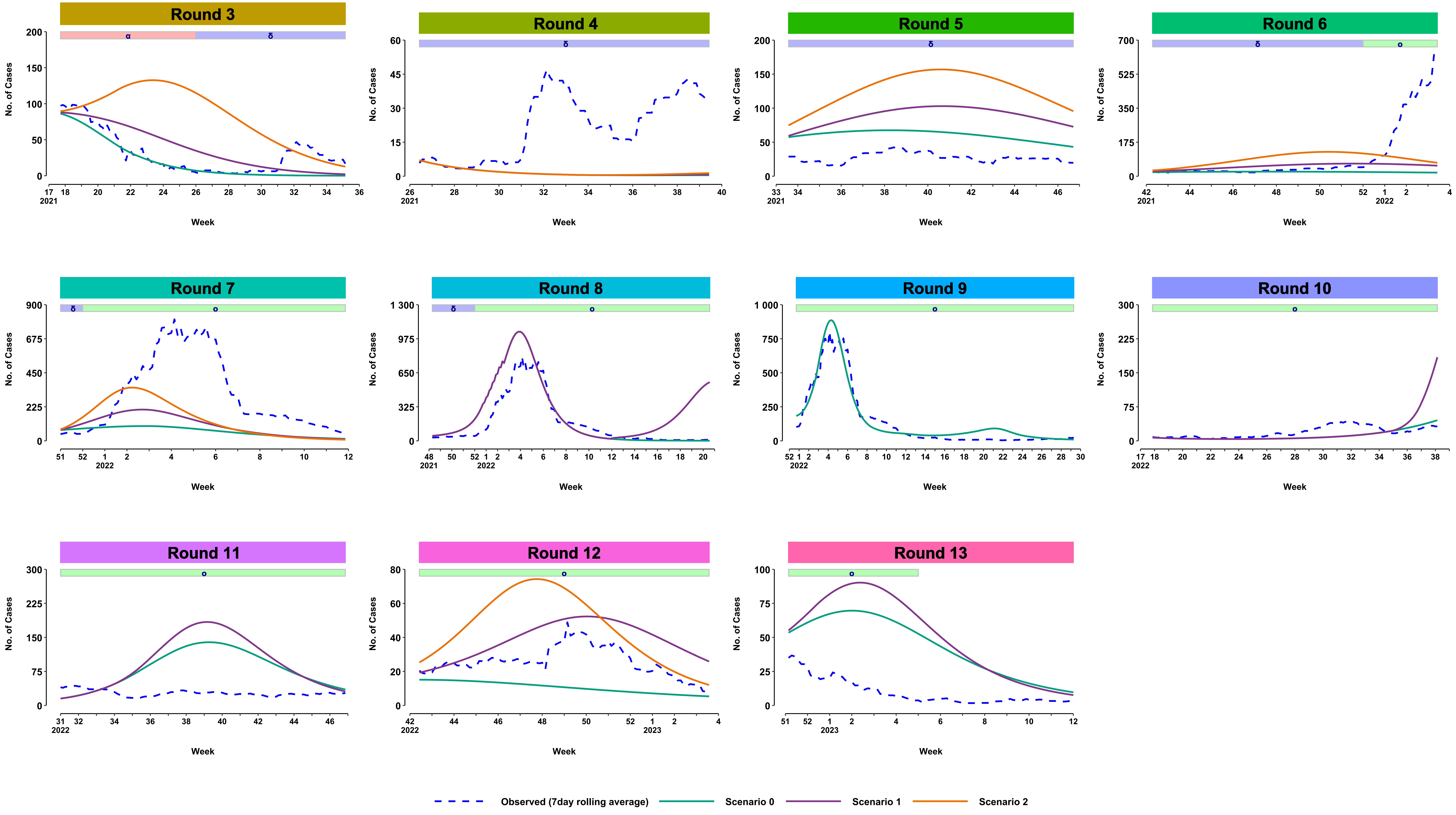

Region : Västernorrland

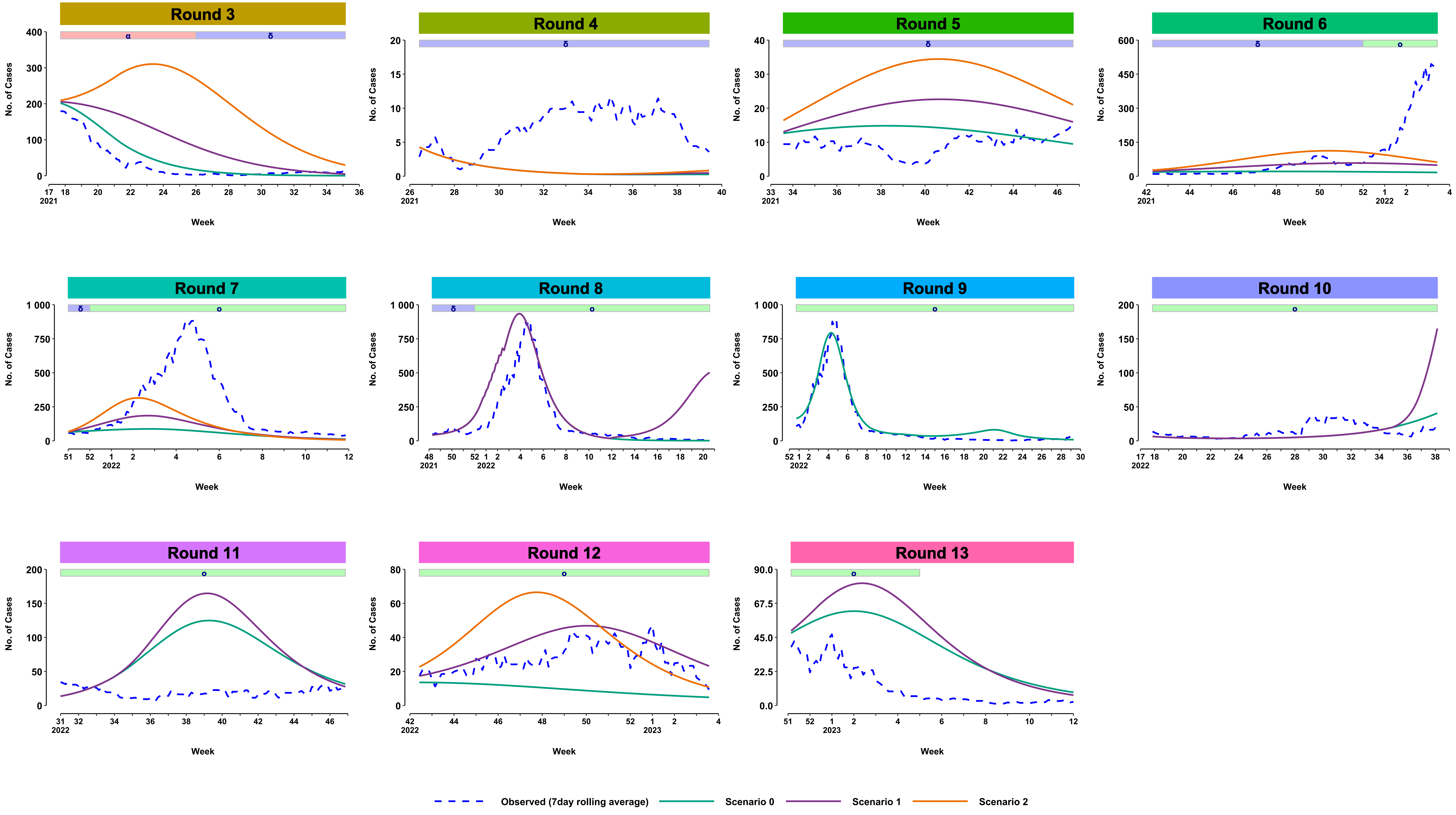

Region : Västmanland

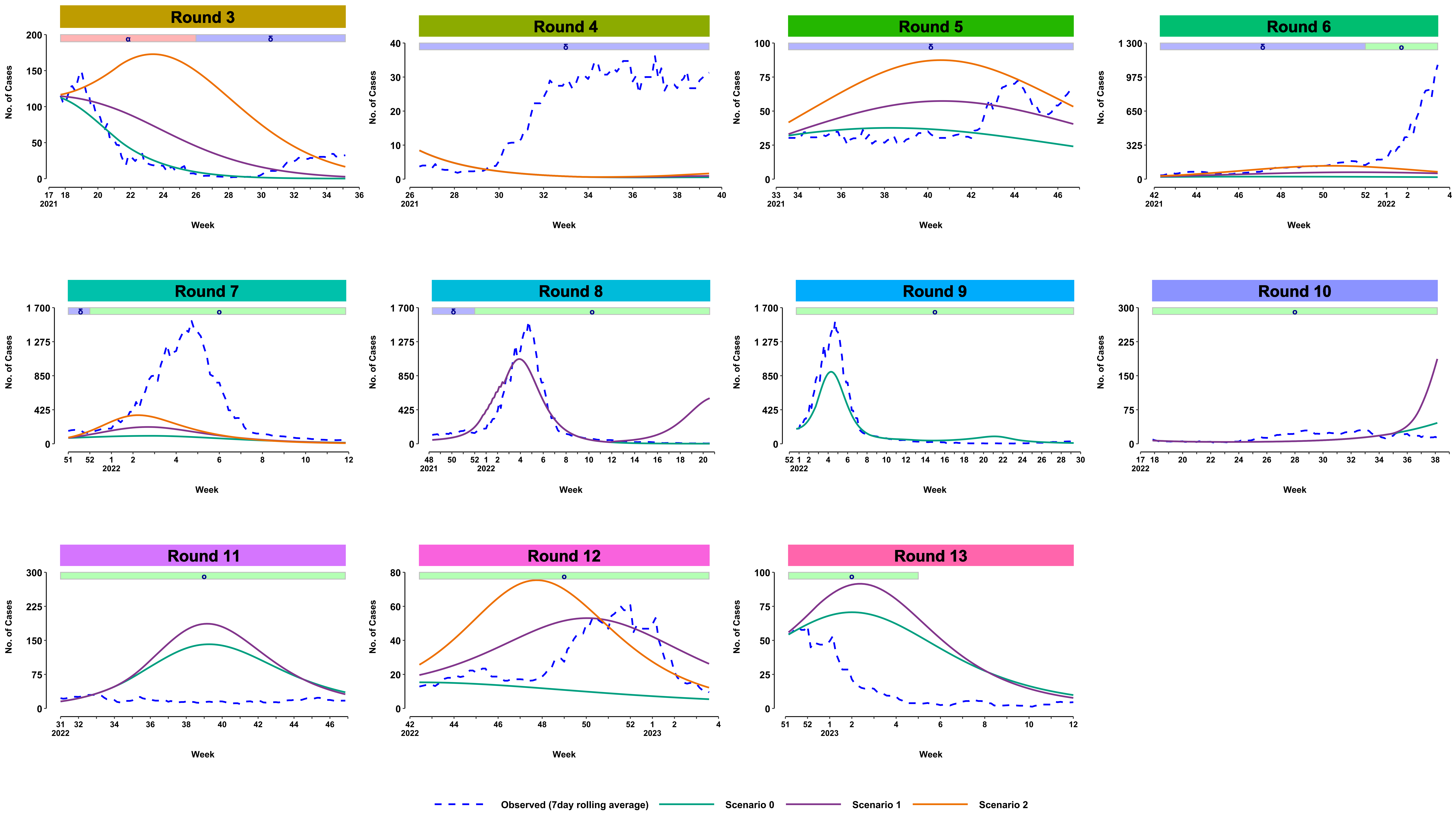

Region : Västra Götaland

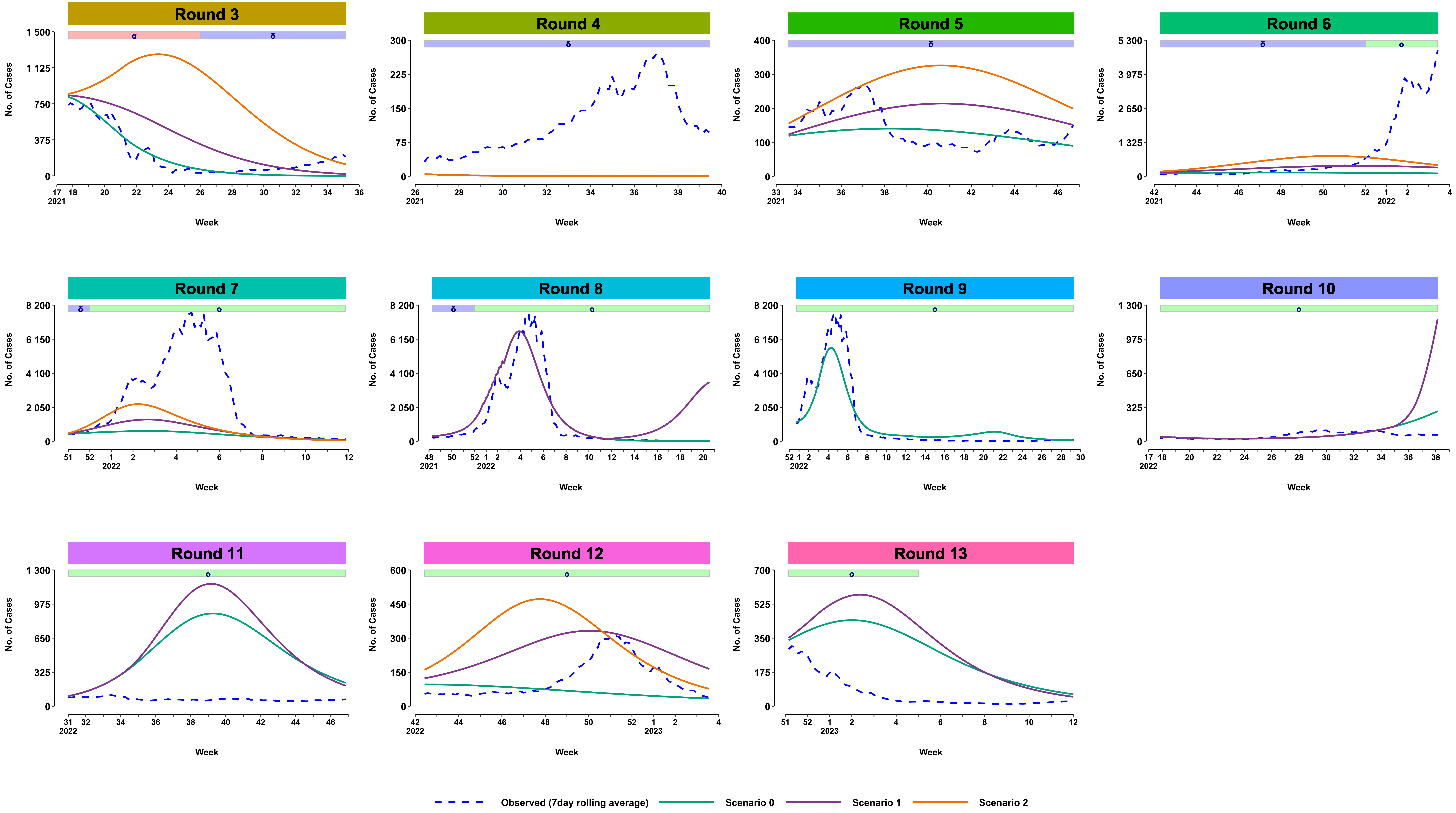

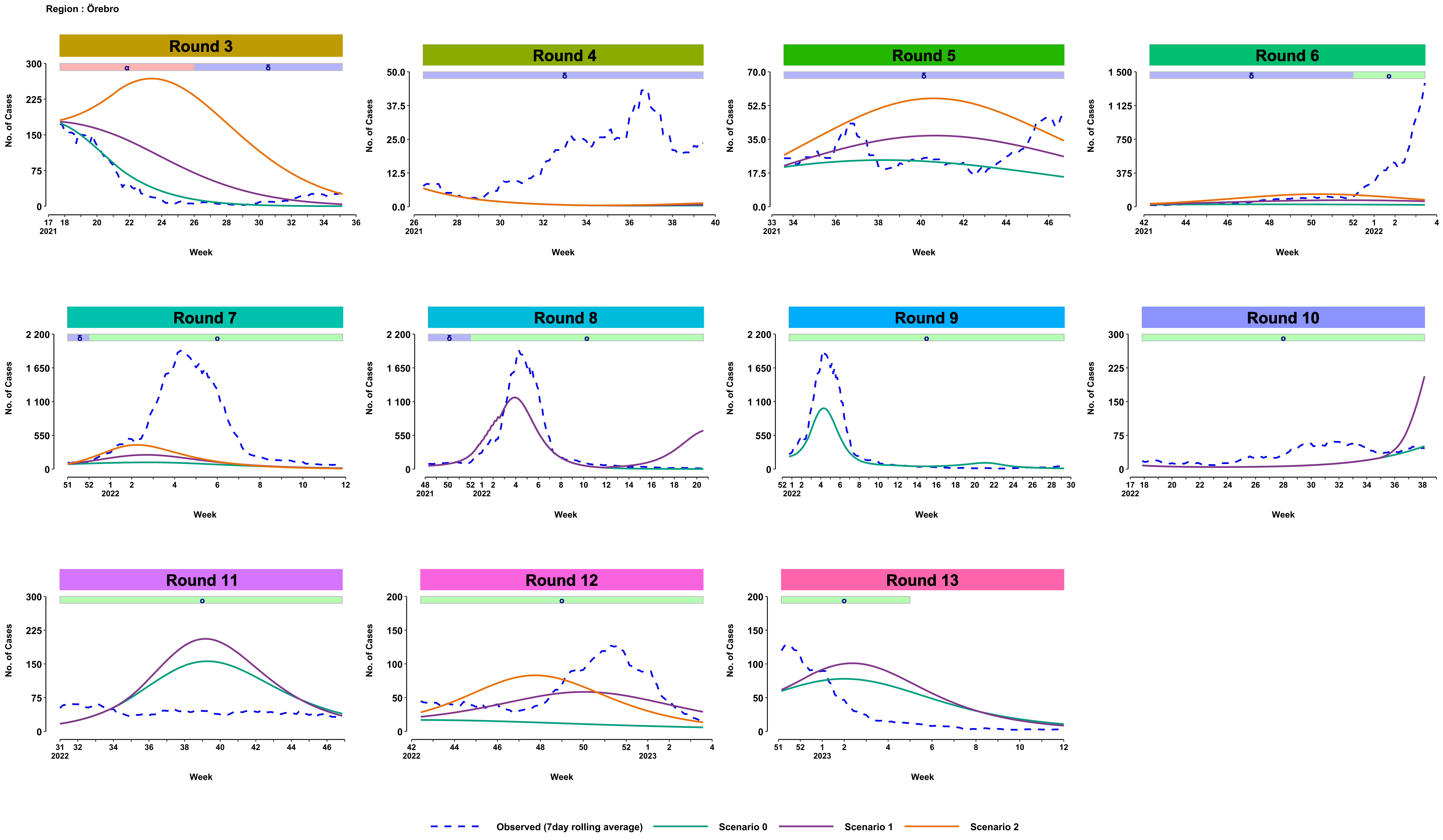
Region : Örebro
Round 3
α
δ
Round 4
δ
Round 5
δ
Round 6
δ
o
Round 7
δ
o
Round 8
δ
o
Round 9
o
Round 10
o
Round 11
o
Round 12
o
Round 13
o
No. of Cases
Week
2021
2022
2023
Observed (7day rolling average)
Scenario 0
Scenario 1
Scenario 2

Region : Östergötland

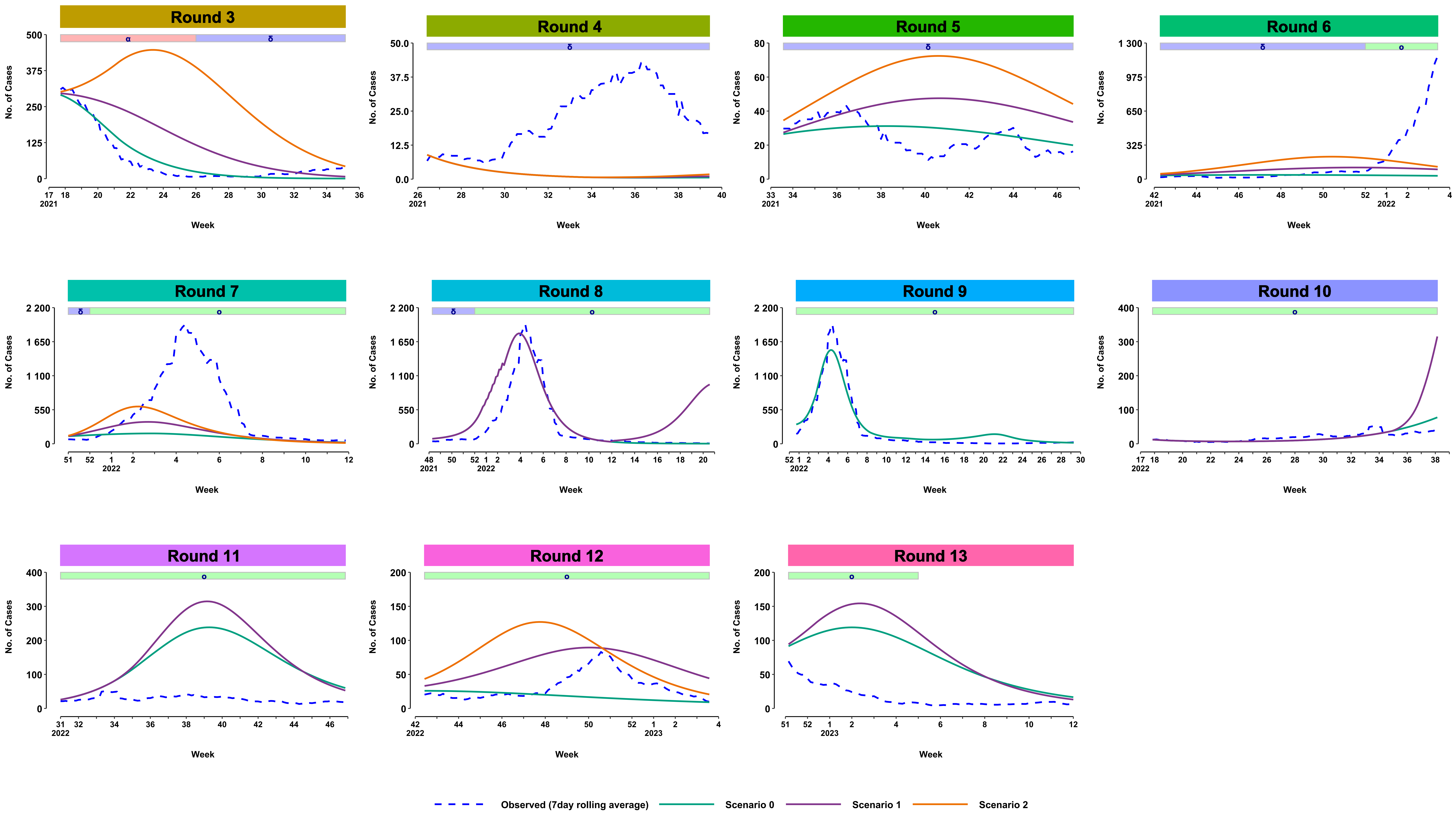

Supplementary Figure 2

Various ROC curves based on different weights systems for SEr with **a)** 3 attributes and **b)** 5 attributes, **c)** DTW, Euclidean distance and MAPE

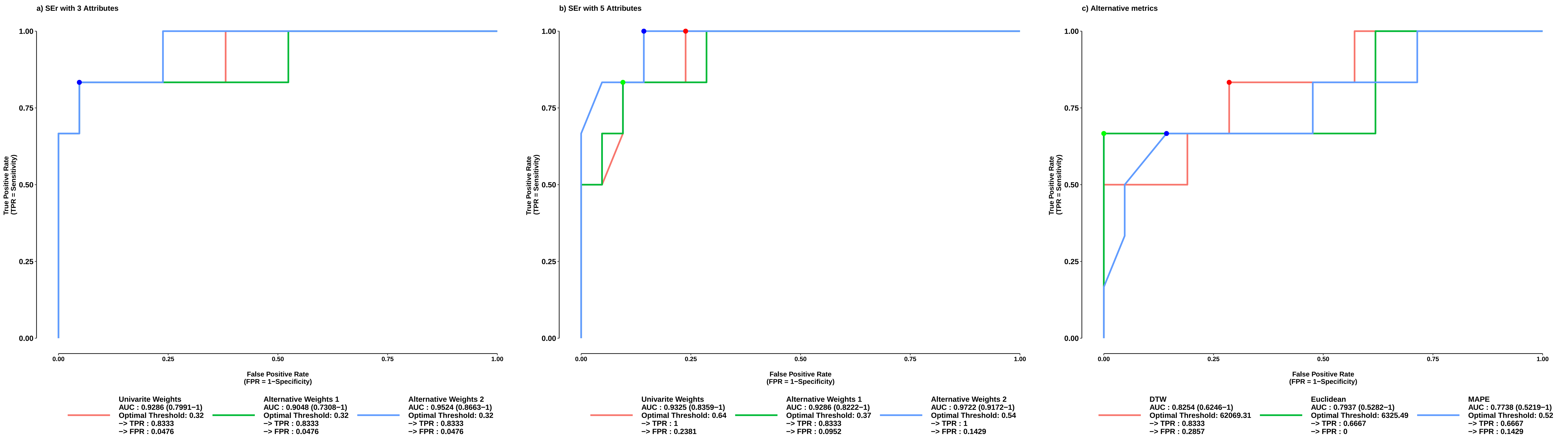

a) SEr with 3 Attributes
b) SEr with 5 Attributes
c) Alternative metrics
1.00
0.75
0.50
0.25
0.00
True Positive Rate
(TPR = Sensitivity)
0.00
0.25
0.50
0.75
1.00
False Positive Rate
(FPR = 1−Specificity)
Univarite Weights
AUC : 0.9286 (0.7991−1)
Optimal Threshold: 0.32
−> TPR : 0.8333
−> FPR : 0.0476
Alternative Weights 1
AUC : 0.9048 (0.7308−1)
Optimal Threshold: 0.32
−> TPR : 0.8333
−> FPR : 0.0476
Alternative Weights 2
AUC : 0.9524 (0.8663−1)
Optimal Threshold: 0.32
−> TPR : 0.8333
−> FPR : 0.0476
Univarite Weights
AUC : 0.9325 (0.8359−1)
Optimal Threshold: 0.64
−> TPR : 1
−> FPR : 0.2381
Alternative Weights 1
AUC : 0.9286 (0.8222−1)
Optimal Threshold: 0.37
−> TPR : 0.8333
−> FPR : 0.0952
Alternative Weights 2
AUC : 0.9722 (0.9172−1)
Optimal Threshold: 0.54
−> TPR : 1
−> FPR : 0.1429
DTW
AUC : 0.8254 (0.6246−1)
Optimal Threshold: 62069.31
−> TPR : 0.8333
−> FPR : 0.2857
Euclidean
AUC : 0.7937 (0.5282−1)
Optimal Threshold: 6325.49
−> TPR : 0.6667
−> FPR : 0
MAPE
AUC : 0.7738 (0.5219−1)
Optimal Threshold: 0.52
−> TPR : 0.6667
−> FPR : 0.1429

Supplementary Figure 3:

a) The four error measures evaluated for each regional simulation round are classified based on thresholds determined through the national ROC analysis. The specific thresholds are as follows: $\varepsilon_{SEr} = 0.52$, $\varepsilon_{DTW} = 62069{,}31$, $\varepsilon_{Euclidean} = 6325{,}49$, $\varepsilon_{MAPE} = 0.52$. **b)** Upset figure displaying how many distinct rounds each error measure or combination of error measures exhibited similarity simultaneously.

Region: Riket (National)

a)

Similar | Not Similar

	Round 3			Round 4			Round 5			Round 6		
	Scenario 0	Scenario 1	Scenario 2	Scenario 0	Scenario 1	Scenario 2	Scenario 0	Scenario 1	Scenario 2	Scenario 0	Scenario 1	Scenario 2
MAPE	0.52	2.13	7.85	0.8	0.79	0.79	0.37	1.03	1.95	0.52	0.58	1.14
Euclidean	5467.61	15811.44	47587.29	6750.81	6723.34	6685.05	3010.85	7647.46	14325.04	86470.52	79437.21	76401.71
DTW	47335.32	35580.59	134956.44	71596.66	70242.37	67883.36	8463.32	69590.15	154211.17	450272.85	380351.56	371656.81
SEr	0.13	0.64	1	0.91	0.9	0.9	0.31	0.87	0.99	0.86	0.67	0.42

	Round 7			Round 8		Round 9	Round 10		Round 11		Round 12			Round 13	
	Scenario 0	Scenario 1	Scenario 2	Scenario 0	Scenario 1	Scenario 0	Scenario 0	Scenario 1	Scenario 0	Scenario 1	Scenario 0	Scenario 1	Scenario 2	Scenario 0	Scenario 1
MAPE	0.65	0.55	0.52	0.45	8.83	3.1	0.48	0.85	3.68	4.4	0.45	0.93	1.53	5.18	5.85
Euclidean	158060.57	141279.15	129261.32	39481.21	85657.71	41247.86	4553.32	14198.3	29328	37419.33	7111.45	6325.49	11846.09	12703.42	16492.3
DTW	957923.88	767768.29	562023.6	62069.31	517241.22	155825.45	18789.48	56423.32	200883.14	255900.52	37578.38	17795.82	43858.79	35269.63	55616.08
SEr	0.81	0.7	0.58	0.11	0.42	0.03	0.36	0.36	0.96	0.96	0.6	0.54	0.92	0.87	0.92

b)

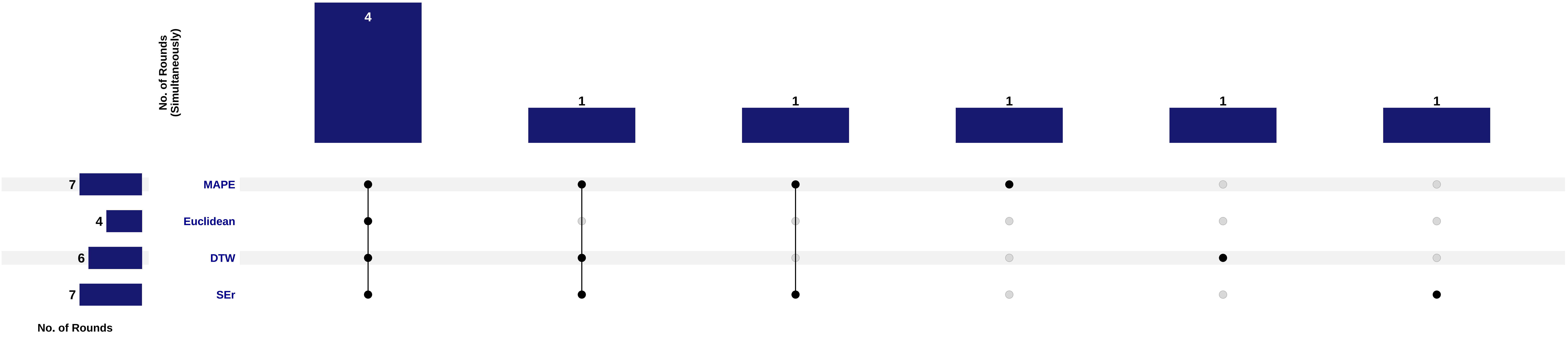

Region: Blekinge

a)

Similar	Not Similar

	Round 3			Round 4			Round 5		
	Scenario 0	Scenario 1	Scenario 2	Scenario 0	Scenario 1	Scenario 2	Scenario 0	Scenario 1	Scenario 2
MAPE	0.62	3.73	14.8	0.79	0.78	0.77	0.83	1.51	2.68
Euclidean	95.46	299.06	826.09	64.18	63.72	63.1	45.12	80.95	141.97
DTW	381.31	350.44	2633.71	516.3	511.92	474.08	278.22	440.56	1129.91
SEr	0.16	0.83	1	0.84	0.84	0.83	0.39	0.86	0.88

	Round 6			Round 7			Round 8		Round 9
	Scenario 0	Scenario 1	Scenario 2	Scenario 0	Scenario 1	Scenario 2	Scenario 0	Scenario 1	Scenario 0
MAPE	0.71	0.82	1.5	0.58	0.47	0.58	0.57	15.1	5.65
Euclidean	726.28	617.02	600.21	1892.48	1688.11	1609.26	1250.84	1716.57	599.13
DTW	4224.05	3163.5	3312.95	10577.21	8076.68	5641.07	1794.79	9000.08	2152.22
SEr	0.82	0.55	0.41	0.76	0.62	0.48	0.36	0.75	0.21

	Round 10		Round 11		Round 12			Round 13	
	Scenario 0	Scenario 1	Scenario 0	Scenario 1	Scenario 0	Scenario 1	Scenario 2	Scenario 0	Scenario 1
MAPE	0.64	1.08	5.89	7	0.64	1.15	2.04	8.59	9.02
Euclidean	71.52	222.34	488.29	612.99	153.08	99.54	189.54	187.68	243.67
DTW	530.01	1113.51	4856.09	5714.02	989.21	459.15	499.12	363.79	411.26
SEr	0.44	0.78	0.82	0.88	0.7	0.4	0.62	0.78	0.85

b)

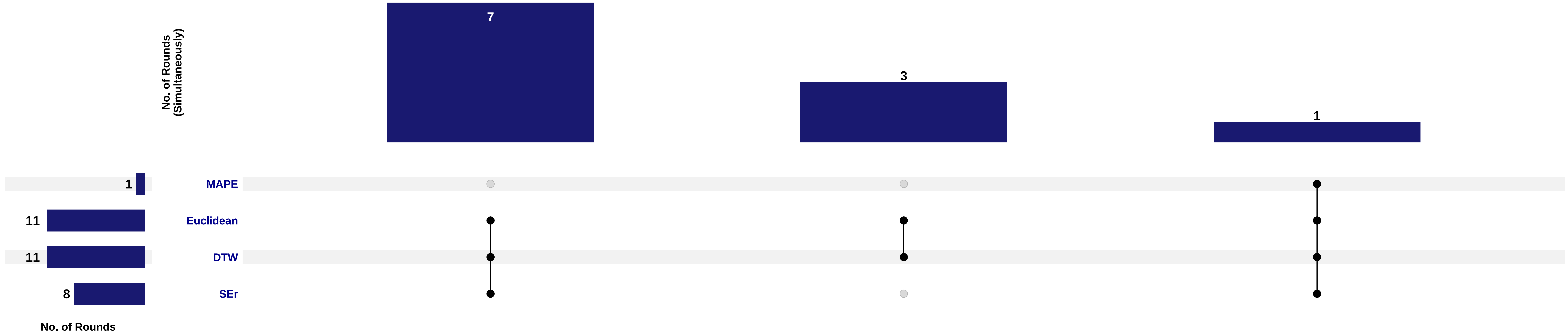

Region: Dalarna

a)

Similar	Not Similar

	Round 3			Round 4			Round 5			Round 6			Round 7		
	Scenario 0	Scenario 1	Scenario 2	Scenario 0	Scenario 1	Scenario 2	Scenario 0	Scenario 1	Scenario 2	Scenario 0	Scenario 1	Scenario 2	Scenario 0	Scenario 1	Scenario 2
MAPE	0.67	4.06	15.71	0.74	0.73	0.73	0.34	0.8	1.47	0.75	1.22	2.15	0.72	0.63	0.59
Euclidean	136.34	502.36	1463.86	174.07	173.28	172.17	68.99	115.36	214.18	2377.18	2182.96	2100.81	4830.33	4391.73	4094.26
DTW	796.36	711	4346.09	1379.68	1351.01	1302.07	448.12	608.78	1181.36	12648.31	11097.66	10596.54	30117.52	24462.91	18838.82
SEr	0.05	0.84	1	0.91	0.91	0.9	0.32	0.54	0.84	0.87	0.68	0.45	0.82	0.72	0.62

	Round 8		Round 9	Round 10		Round 11		Round 12			Round 13	
	Scenario 0	Scenario 1	Scenario 0	Scenario 0	Scenario 1	Scenario 0	Scenario 1	Scenario 0	Scenario 1	Scenario 2	Scenario 0	Scenario 1
MAPE	0.52	4.96	1.38	0.69	0.75	1.67	2.08	0.67	0.29	0.72	5.19	5.39
Euclidean	1659.16	2649.34	1716.67	407.48	460.81	660.44	875.81	435.28	192.37	319.15	294.14	379.64
DTW	4286.19	16166.86	6918.36	1835.46	1816.13	2348.62	3610.22	3183.93	1015.02	508.39	715.27	624.04
SEr	0.05	0.28	0.15	0.58	0.48	0.66	0.88	0.8	0.25	0.24	0.75	0.88

b)

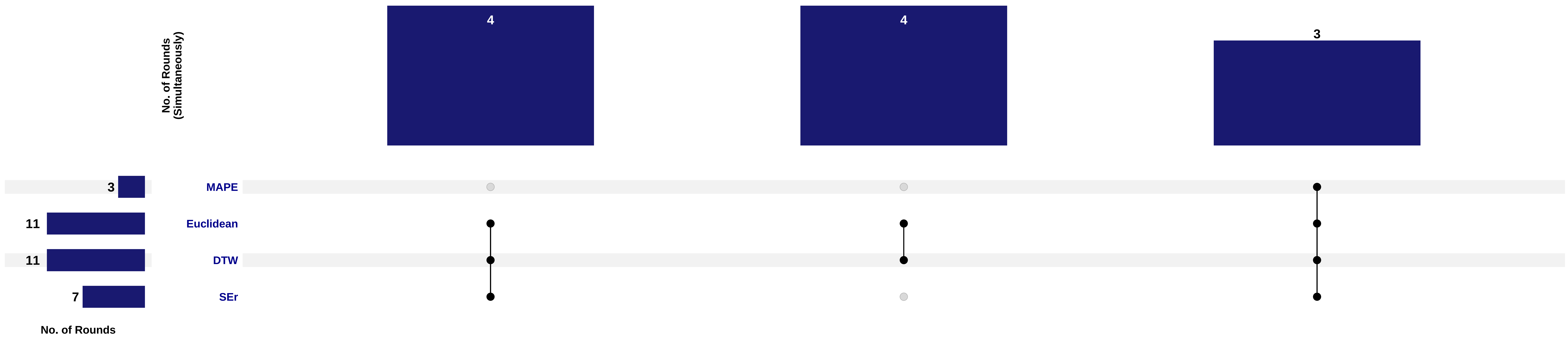

Region: Gotland

a)

Similar | Not Similar

	Round 3			Round 4			Round 5			Round 6			Round 7			Round 8		Round 9	Round 10		Round 11		Round 12			Round 13	
	Scenario 0	Scenario 1	Scenario 2	Scenario 0	Scenario 1	Scenario 2	Scenario 0	Scenario 1	Scenario 2	Scenario 0	Scenario 1	Scenario 2	Scenario 0	Scenario 1	Scenario 2	Scenario 0	Scenario 1	Scenario 0	Scenario 0	Scenario 1	Scenario 0	Scenario 1	Scenario 0	Scenario 1	Scenario 2	Scenario 0	Scenario 1
MAPE	0.71	2.44	7.67	0.89	0.89	0.88	33.61	54.51	81	3.55	5.49	8.32	0.72	0.68	0.71	0.81	13.49	7.2	0.95	1.39	10.74	12.2	1.34	3.23	5.02	Inf	Inf
Euclidean	81.83	96.21	185.35	80.59	80.57	80.54	232.53	356.58	528.99	467.47	434.3	428.47	850.16	755.91	692.73	288.17	527.67	211.83	31.85	84.81	189.11	235.4	52.71	49.69	81.51	74.4	95.92
DTW	573.81	568.14	1010.89	502.95	502.12	500.91	3251.34	4959.97	7176.74	2193.25	2097.74	2158.58	5116.66	4137.83	2916.61	912.71	3638.57	996.32	197	417.38	1704.48	1955.18	312.78	248.45	242.06	167.87	179.8
SEr	0.56	0.4	0.86	0.97	0.97	0.97	1	1	1	0.85	0.64	0.44	0.78	0.67	0.54	0.19	0.49	0.1	0.37	0.74	0.85	0.84	0.66	0.61	0.7	0.7	0.83

b)

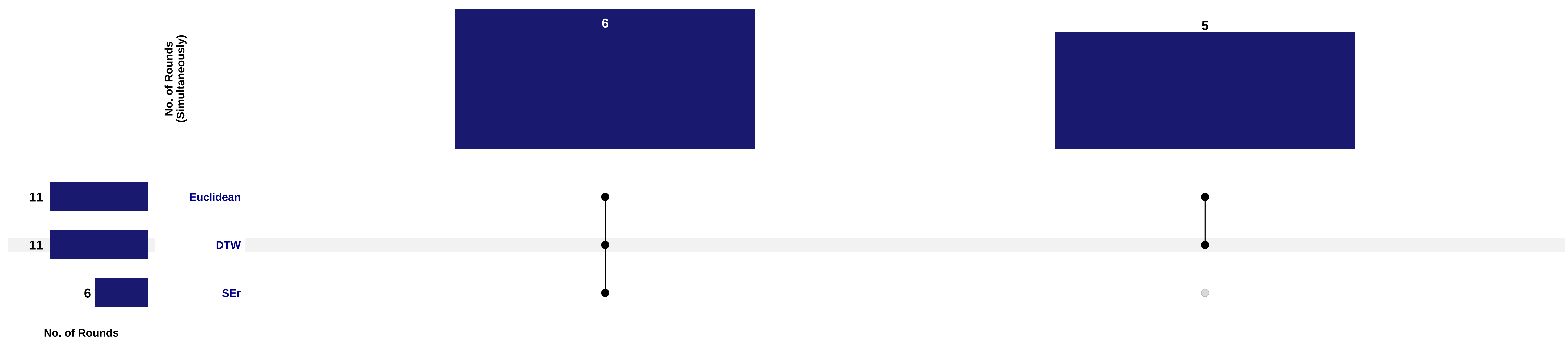

Region: Gävleborg

a)

Similar | Not Similar

	Round 3			Round 4			Round 5			Round 6		
	Scenario 0	Scenario 1	Scenario 2	Scenario 0	Scenario 1	Scenario 2	Scenario 0	Scenario 1	Scenario 2	Scenario 0	Scenario 1	Scenario 2
MAPE	0.98	4.55	15.98	0.84	0.84	0.84	0.56	1.09	2.02	0.57	0.49	0.92
Euclidean	137.15	465.37	1334.81	123.39	123.03	122.55	93.89	176.77	331.59	2004.01	1817.63	1752.95
DTW	1011.62	907.79	4641.7	1248.4	1231.06	1200.39	696.22	769.17	2427.63	10443.48	8226.16	8003.18
SEr	0.05	0.83	0.89	0.93	0.93	0.93	0.39	0.78	1	0.85	0.64	0.38

	Round 7			Round 8		Round 9	Round 10		Round 11		Round 12			Round 13	
	Scenario 0	Scenario 1	Scenario 2	Scenario 0	Scenario 1	Scenario 0	Scenario 0	Scenario 1	Scenario 0	Scenario 1	Scenario 0	Scenario 1	Scenario 2	Scenario 0	Scenario 1
MAPE	0.66	0.56	0.62	0.5	15.37	10.53	1.31	2.11	11.93	13.91	1.02	3.39	4.95	7.81	8.81
Euclidean	4353.93	3943.09	3708.71	1736.83	2744.6	1246.69	139.05	424.96	938.59	1161.96	91.8	284.89	411.4	403.69	508.93
DTW	25136.91	20374.82	15619	3587.97	16796.24	6351.55	965.72	2019.28	9802.32	11448.87	642.44	2477.73	3328.97	2607.47	3224.1
SEr	0.8	0.69	0.58	0.14	0.44	0.1	1	1	1	1	0.4	0.84	0.96	0.87	0.92

b)

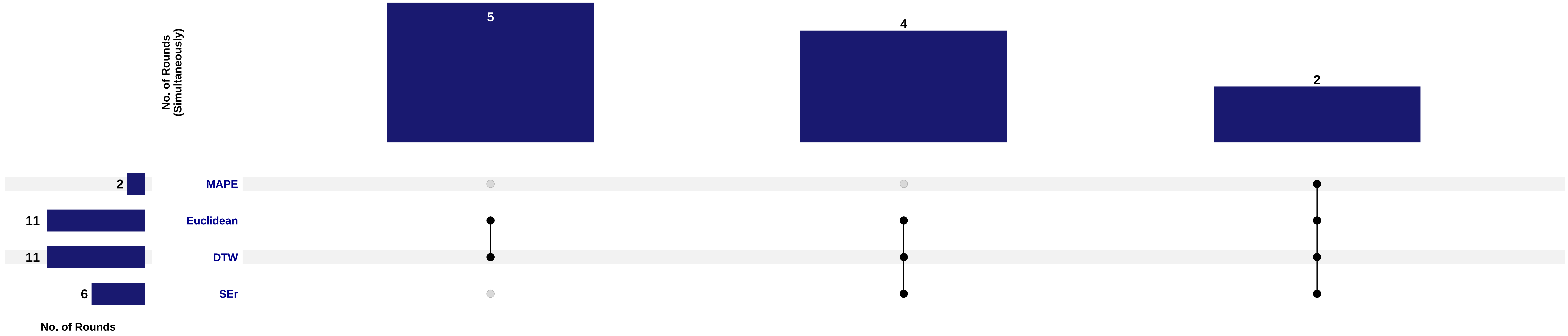

Region: Halland

a)

Similar	Not Similar

	Round 3			Round 4			Round 5			Round 6		
	Scenario 0	Scenario 1	Scenario 2	Scenario 0	Scenario 1	Scenario 2	Scenario 0	Scenario 1	Scenario 2	Scenario 0	Scenario 1	Scenario 2
MAPE	0.65	2.14	7.26	0.86	0.86	0.85	0.94	1.95	3.33	0.75	0.87	1.45
Euclidean	218.88	513.19	1436.23	227.74	227.09	226.19	160.53	325.12	553.87	4174.75	3950.23	3830.09
DTW	1945.93	1813.29	6180.88	2445.23	2438.95	2429.78	782.26	2773.34	5716.73	21138.18	18615.74	18565.81
SEr	0.11	0.72	1	0.93	0.93	0.93	0.58	0.97	0.96	0.9	0.76	0.6

	Round 7			Round 8		Round 9	Round 10		Round 11		Round 12			Round 13	
	Scenario 0	Scenario 1	Scenario 2	Scenario 0	Scenario 1	Scenario 0	Scenario 0	Scenario 1	Scenario 0	Scenario 1	Scenario 0	Scenario 1	Scenario 2	Scenario 0	Scenario 1
MAPE	0.71	0.64	0.6	0.43	7.55	2.66	0.58	0.99	3.47	4.3	0.57	0.53	1.18	1.96	2.28
Euclidean	8132.75	7579.63	7137.68	3082.36	3936.73	4179.31	217.3	492.16	966.72	1236.44	396.42	193.63	387.89	325.26	438.8
DTW	48546.07	42228.62	34451.48	12592.37	27340.08	17704.33	925.45	2192.05	8327.69	9761.96	2697.12	701.76	496.85	600.35	511.91
SEr	0.85	0.77	0.7	0.24	0.12	0.32	0.45	0.43	0.96	1	0.75	0.19	0.38	0.7	0.74

b)

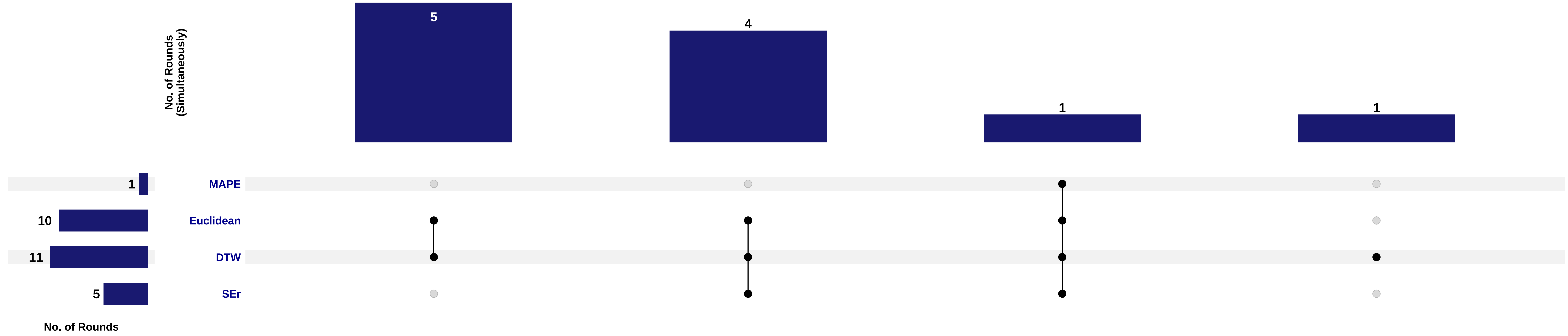

Region: Jämtland

a)

Similar | Not Similar

	Round 3			Round 4			Round 5			Round 6			Round 7			Round 8		Round 9	Round 10		Round 11		Round 12			Round 13	
	Scenario 0	Scenario 1	Scenario 2	Scenario 0	Scenario 1	Scenario 2	Scenario 0	Scenario 1	Scenario 2	Scenario 0	Scenario 1	Scenario 2	Scenario 0	Scenario 1	Scenario 2	Scenario 0	Scenario 1	Scenario 0	Scenario 0	Scenario 1	Scenario 0	Scenario 1	Scenario 0	Scenario 1	Scenario 2	Scenario 0	Scenario 1
MAPE	1.3	6.09	21.29	0.84	0.84	0.83	1.32	2.57	4.2	1	1.64	2.91	0.7	0.63	0.6	0.44	7.5	2.82	0.67	1.23	5.3	6.47	0.49	1.01	1.72	7.54	7.99
Euclidean	80.86	230.51	675.09	67.23	67.07	66.86	62.24	120.82	199.84	1857.6	1775.48	1734.62	2391.34	2149.95	1923.07	550.14	1102.4	1141.37	121.7	215.88	404.51	507.23	83.66	84.09	153.8	158.14	205.55
DTW	562.27	554.08	1751.25	580.36	572.54	559.15	315.21	1067.17	2201.77	8883.78	8183.91	8153.05	14919.91	12631.44	9827.23	2159.21	7878.02	3621.83	675.5	1224.09	2156.06	2711	500.67	283.68	676.78	581.54	852.5
SEr	0.06	0.64	0.87	0.94	0.94	0.94	0.86	0.89	0.9	0.92	0.79	0.68	0.83	0.74	0.64	0.08	0.24	0.21	0.57	0.42	0.95	0.93	0.64	0.49	0.92	0.74	0.91

b)

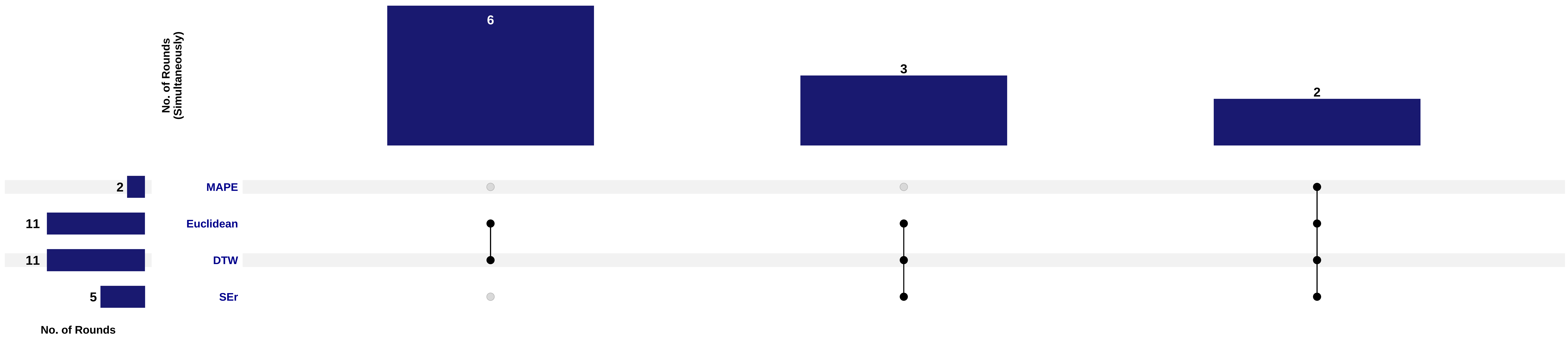

Region: Jönköping

a)

Similar	Not Similar

	Round 3			Round 4			Round 5			Round 6		
	Scenario 0	Scenario 1	Scenario 2	Scenario 0	Scenario 1	Scenario 2	Scenario 0	Scenario 1	Scenario 2	Scenario 0	Scenario 1	Scenario 2
MAPE	0.43	1.67	6.99	0.69	0.69	0.68	0.31	0.85	1.65	0.72	1.14	2.22
Euclidean	180.23	437.94	1454.79	196.94	195.46	193.39	64.88	160.05	306.54	2695.88	2483.77	2438.12
DTW	1549.08	1316.19	3558.84	1864.96	1807.08	1706.97	345.55	845.66	2455.86	13136.93	10985.64	11457.78
SEr	0.17	0.43	0.94	0.85	0.84	0.84	0.18	0.58	0.96	0.84	0.62	0.35

	Round 7			Round 8		Round 9	Round 10		Round 11		Round 12			Round 13	
	Scenario 0	Scenario 1	Scenario 2	Scenario 0	Scenario 1	Scenario 0	Scenario 0	Scenario 1	Scenario 0	Scenario 1	Scenario 0	Scenario 1	Scenario 2	Scenario 0	Scenario 1
MAPE	0.56	0.49	0.56	0.62	15.37	5.2	0.43	0.73	3.7	4.46	0.42	0.95	1.53	3.65	4.26
Euclidean	5130.46	4568.93	4203.18	1842.92	3264.4	1216.73	129.98	473.1	1055.87	1343.8	220.66	236.46	425.03	437.91	572.68
DTW	29425.05	23913.67	17385.87	3881.04	20480.36	6263.07	473.14	1793.63	10113.26	11824.5	1206.93	711.43	1765.69	1363.92	2123.06
SEr	0.78	0.66	0.53	0.24	0.57	0.13	0.37	0.61	1	1	0.6	0.58	0.92	0.75	0.93

b)

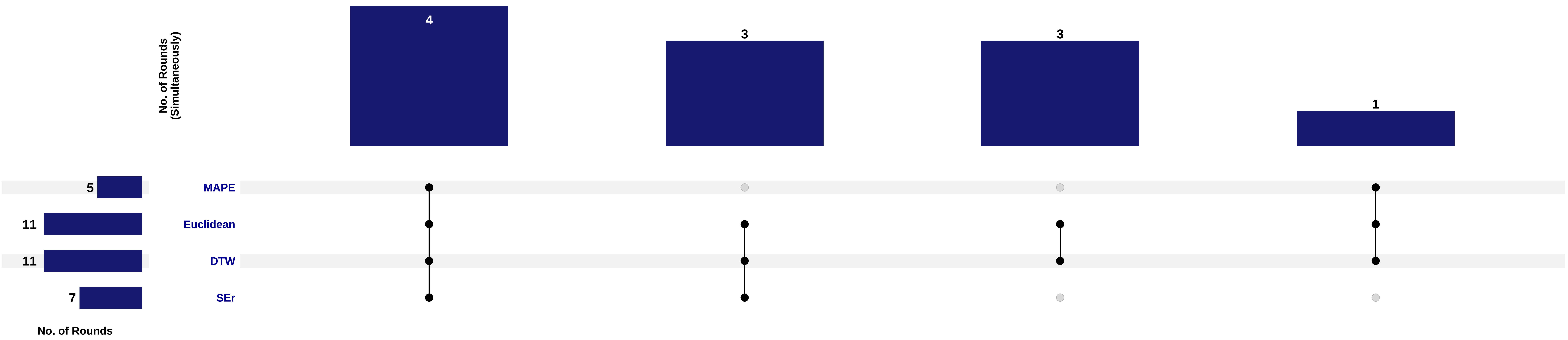

Region: Kalmar

a)

Similar | Not Similar

	Round 3			Round 4			Round 5			Round 6		
	Scenario 0	Scenario 1	Scenario 2	Scenario 0	Scenario 1	Scenario 2	Scenario 0	Scenario 1	Scenario 2	Scenario 0	Scenario 1	Scenario 2
MAPE	0.78	4.17	16.01	0.82	0.81	0.8	0.47	0.66	1.14	0.56	0.36	0.79
Euclidean	159.33	553.91	1604.74	114.47	113.87	113.06	98.64	92.45	148.9	1502.46	1337.09	1285.24
DTW	970.81	852.89	4066.59	929.88	922.42	911.09	824.31	643.59	472.9	8250.78	6204.4	6014.29
SEr	0.01	0.78	0.95	0.87	0.87	0.86	0.48	0.28	0.74	0.85	0.64	0.37

	Round 7			Round 8		Round 9	Round 10		Round 11		Round 12			Round 13	
	Scenario 0	Scenario 1	Scenario 2	Scenario 0	Scenario 1	Scenario 0	Scenario 0	Scenario 1	Scenario 0	Scenario 1	Scenario 0	Scenario 1	Scenario 2	Scenario 0	Scenario 1
MAPE	0.69	0.6	0.64	0.54	10.15	3.73	0.45	0.73	2.54	3.06	0.59	0.4	0.98	3.83	4.21
Euclidean	4199.78	3881.38	3724	2032.36	2716.94	1748.68	96.68	316.25	652.47	845.93	254.32	110.31	255.12	257.92	342.62
DTW	24807.37	20166.48	15893.82	2983.18	14063.99	5521.21	395.34	1271.95	4761.34	6021.81	1939.63	374.66	360.61	444.04	638.13
SEr	0.82	0.72	0.62	0.05	0.31	0.11	0.25	0.37	0.92	0.92	0.74	0.18	0.49	0.76	0.78

b)

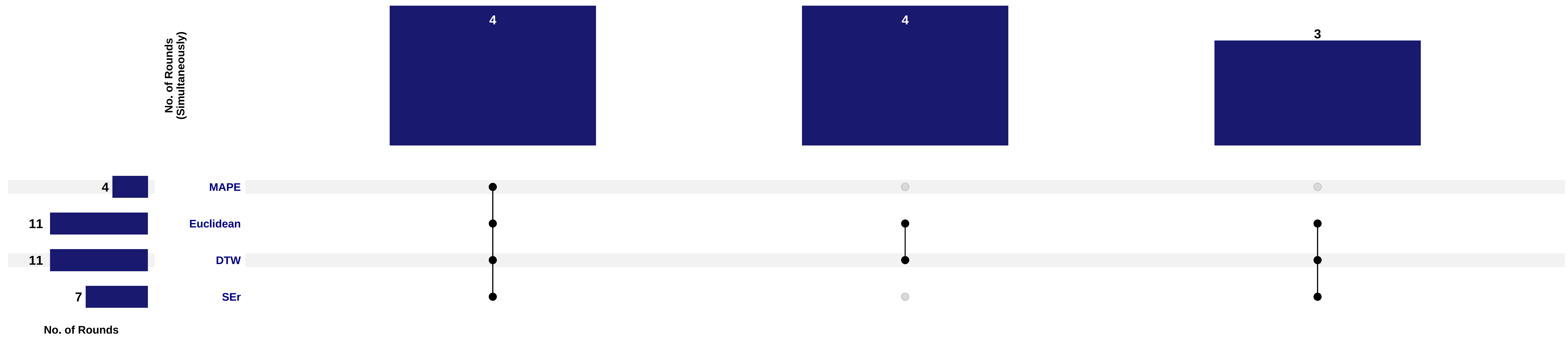

Region: Kronoberg

a)

Similar	Not Similar

	Round 3			Round 4			Round 5			Round 6			Round 7			Round 8		Round 9	Round 10		Round 11		Round 12			Round 13	
	Scenario 0	Scenario 1	Scenario 2	Scenario 0	Scenario 1	Scenario 2	Scenario 0	Scenario 1	Scenario 2	Scenario 0	Scenario 1	Scenario 2	Scenario 0	Scenario 1	Scenario 2	Scenario 0	Scenario 1	Scenario 0	Scenario 0	Scenario 1	Scenario 0	Scenario 1	Scenario 0	Scenario 1	Scenario 2	Scenario 0	Scenario 1
MAPE	0.48	2.11	8.88	0.76	0.75	0.74	0.33	0.49	0.93	0.71	1.38	2.71	0.68	0.61	0.64	0.66	12.31	3.98	0.45	0.85	3.14	3.86	0.56	0.5	1.02	2.68	2.96
Euclidean	166.31	391.68	1218.05	116.56	115.51	114.13	69	67.13	94.45	1567.6	1457.59	1440.77	3289.47	2987.54	2796	1092.22	1843.92	987.65	86.71	278.59	571.53	732.88	228.81	109.66	216.09	212.78	284.72
DTW	1176.45	1018.24	2508.4	878.88	867.96	851.65	479.68	389.39	400.42	7221.21	6527.83	6830.81	19489.47	15980.94	12544.13	3172.15	12100.32	4733.13	436.69	1172.41	4936.98	6043.16	1468.73	487.32	341.12	442.54	433.98
SEr	0.14	0.5	0.99	0.8	0.79	0.78	0.42	0.54	0.58	0.85	0.64	0.38	0.81	0.7	0.59	0.12	0.41	0.09	0.32	0.56	0.87	0.93	0.74	0.23	0.4	0.72	0.84

b)

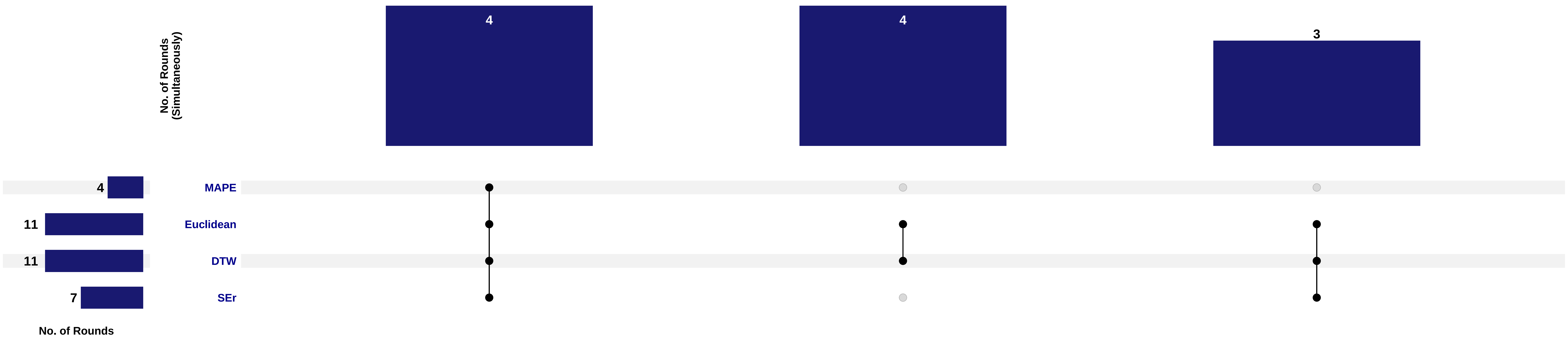

Region: Norrbotten

a)

Similar	Not Similar

	Round 3			Round 4			Round 5			Round 6			Round 7		
	Scenario 0	Scenario 1	Scenario 2	Scenario 0	Scenario 1	Scenario 2	Scenario 0	Scenario 1	Scenario 2	Scenario 0	Scenario 1	Scenario 2	Scenario 0	Scenario 1	Scenario 2
MAPE	0.64	0.76	4.62	0.71	0.69	0.65	0.68	0.69	1.04	0.44	0.88	1.99	0.63	0.56	0.66
Euclidean	397.03	227.82	860.28	71.99	69.21	65.26	102.38	70.6	95.82	1108.56	970.23	1003.14	2104.82	1749.27	1618.71
DTW	1284.53	1261.1	1548.62	434.3	387.48	318.24	942.85	407.9	344.07	5496.01	4579.24	4802.17	12733.62	8808.11	4818.37
SEr	0.44	0.07	0.82	0.39	0.37	0.35	0.56	0.34	0.38	0.79	0.49	0.32	0.72	0.54	0.35

	Round 8		Round 9	Round 10		Round 11		Round 12			Round 13	
	Scenario 0	Scenario 1	Scenario 0	Scenario 0	Scenario 1	Scenario 0	Scenario 1	Scenario 0	Scenario 1	Scenario 2	Scenario 0	Scenario 1
MAPE	0.89	10.15	2.98	0.67	1.3	5.4	6.61	0.31	1.62	1.94	7.89	9.09
Euclidean	2209.95	2868.44	1123.62	118.87	362.7	762.1	960.68	56.66	220.66	311.29	358.31	449.58
DTW	6460.57	17435.42	4243.28	824.98	1730.24	6650.21	8075.35	281.44	1578.46	2253.4	2147.11	2698.21
SEr	0.69	0.88	0.43	0.58	0.71	1	1	0.47	0.86	0.97	0.87	0.92

b)

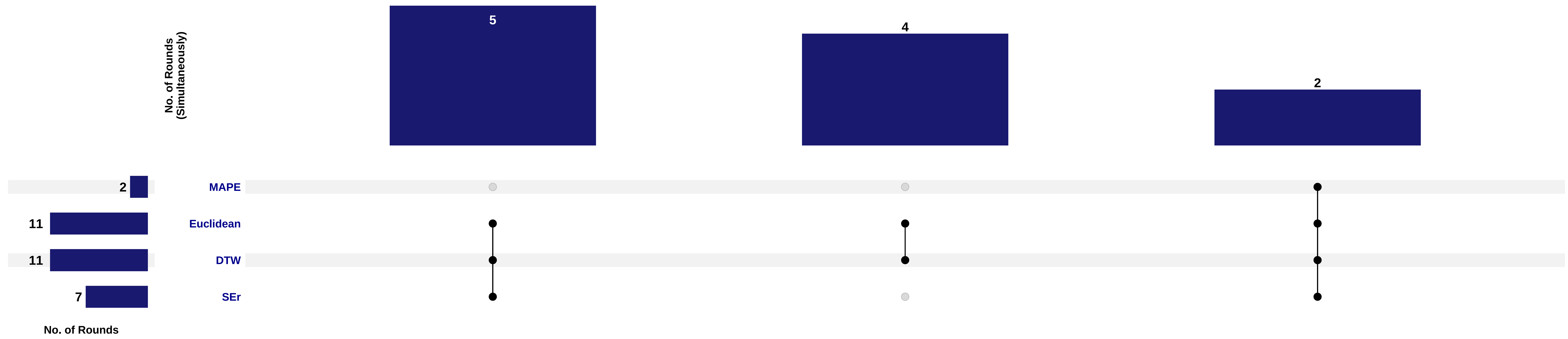

Region: Skåne

a)

Similar | Not Similar

	Round 3			Round 4			Round 5			Round 6			Round 7			Round 8		Round 9	Round 10		Round 11		Round 12			Round 13	
	Scenario 0	Scenario 1	Scenario 2	Scenario 0	Scenario 1	Scenario 2	Scenario 0	Scenario 1	Scenario 2	Scenario 0	Scenario 1	Scenario 2	Scenario 0	Scenario 1	Scenario 2	Scenario 0	Scenario 1	Scenario 0	Scenario 0	Scenario 1	Scenario 0	Scenario 1	Scenario 0	Scenario 1	Scenario 2	Scenario 0	Scenario 1
MAPE	0.52	1.7	6.38	0.8	0.8	0.79	0.45	0.91	1.69	0.6	0.64	1.05	0.6	0.51	0.46	0.45	14.73	6.01	0.68	1.34	6.71	8.04	0.59	1.48	2.43	5.34	6.09
Euclidean	949.65	1544.86	4724.7	906.7	903.47	899.07	402.61	736.73	1365.82	11842.22	10783.11	10184.96	19116.04	16772.41	14985.33	5744.27	11768.39	5303.93	618.46	2012.69	4354.37	5445.94	992.53	981.26	1740.81	1693.21	2197.84
DTW	7595.79	6270.63	8986.84	9201	9022.55	8716.42	3033.06	3636.51	10271.58	68213.55	57912.53	56648.57	127849.18	96965.47	65919.27	16026.45	79687.03	16505.11	4313.1	9349.48	43805.13	51778.12	5842.82	3629.05	5901.15	3750.02	6150.74
SEr	0.27	0.28	1	0.91	0.91	0.9	0.38	0.63	0.9	0.88	0.71	0.5	0.8	0.68	0.56	0.18	0.49	0.1	0.43	0.89	1	1	0.58	0.64	0.93	0.87	0.92

b)

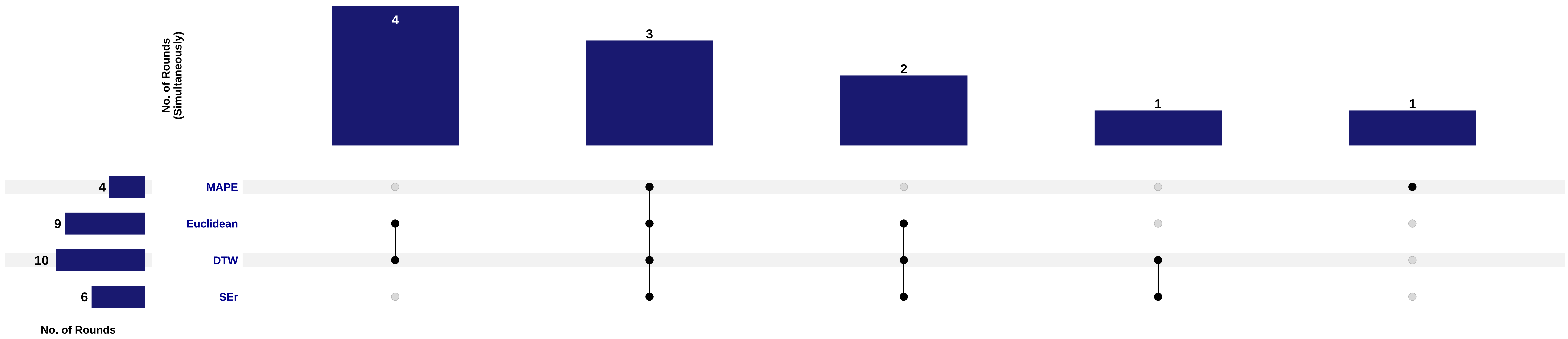

Region: Stockholm

a)

Similar | Not Similar

	Round 3			Round 4			Round 5			Round 6			Round 7		
	Scenario 0	Scenario 1	Scenario 2	Scenario 0	Scenario 1	Scenario 2	Scenario 0	Scenario 1	Scenario 2	Scenario 0	Scenario 1	Scenario 2	Scenario 0	Scenario 1	Scenario 2
MAPE	0.74	2.54	8.27	0.88	0.88	0.88	0.74	1.62	2.82	0.54	0.52	0.94	0.58	0.49	0.44
Euclidean	1853.97	3963.27	10817.38	2123.09	2119.05	2113.38	1573.91	3385.45	5877.54	23892.41	22183.75	21256.31	34377.16	30123.51	26457.47
DTW	15058.15	12378.19	27703.96	21959.95	21717.44	21300.23	12467.37	41985.26	74218.07	127755.25	110248.1	108472.51	216138.26	165670.64	119168.95
SEr	0.16	0.55	1	0.95	0.95	0.95	0.66	1	1	0.88	0.73	0.53	0.8	0.68	0.56

	Round 8		Round 9	Round 10		Round 11		Round 12			Round 13	
	Scenario 0	Scenario 1	Scenario 0	Scenario 0	Scenario 1	Scenario 0	Scenario 1	Scenario 0	Scenario 1	Scenario 2	Scenario 0	Scenario 1
MAPE	0.56	7.33	2.64	0.56	0.91	4.59	5.36	0.52	1.87	2.63	7.74	8.77
Euclidean	8045.11	19209.26	11576.76	1201.36	3307.79	6557.63	8177.96	880.01	2054.09	3129.2	3276.25	4155.3
DTW	15786.68	116942.54	35847.59	5748.27	14439.67	45958.38	36413.14	4914.63	14020.95	21495.13	17422.47	22616.24
SEr	0.15	0.48	0.07	0.43	0.36	0.83	0.83	0.39	0.89	0.96	0.91	0.93

b)

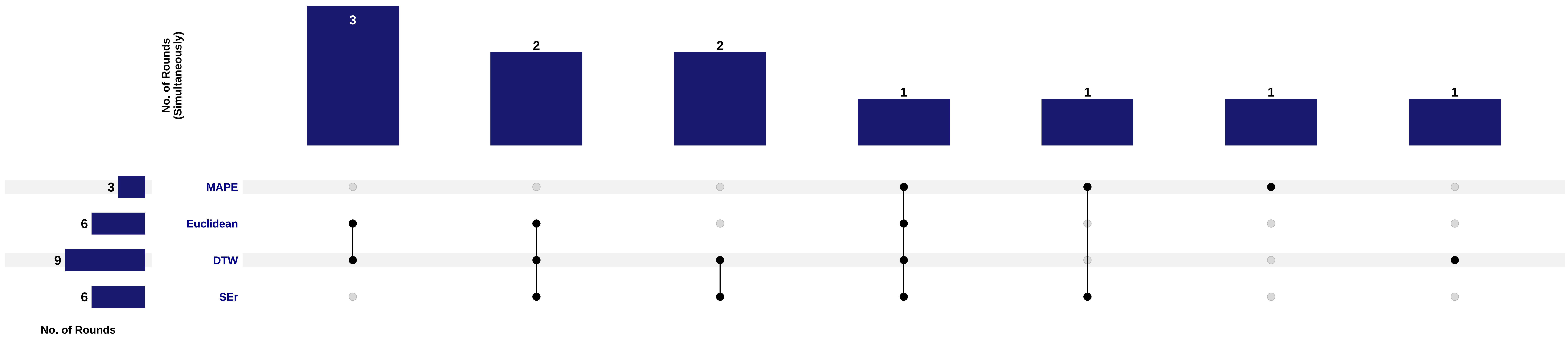

Region: Sörmland

a)

Similar	Not Similar

	Round 3			Round 4			Round 5			Round 6			Round 7			Round 8		Round 9	Round 10		Round 11		Round 12			Round 13	
	Scenario 0	Scenario 1	Scenario 2	Scenario 0	Scenario 1	Scenario 2	Scenario 0	Scenario 1	Scenario 2	Scenario 0	Scenario 1	Scenario 2	Scenario 0	Scenario 1	Scenario 2	Scenario 0	Scenario 1	Scenario 0	Scenario 0	Scenario 1	Scenario 0	Scenario 1	Scenario 0	Scenario 1	Scenario 2	Scenario 0	Scenario 1
MAPE	0.77	3.47	12.53	0.82	0.82	0.81	0.32	0.73	1.51	0.5	0.71	1.58	0.67	0.61	0.66	0.62	6.88	2.33	0.52	0.75	2.8	3.41	0.58	0.45	0.86	6.06	6.52
Euclidean	133.21	482.02	1400.33	130.84	130.41	129.81	58.55	125.3	258.5	2496.6	2324.24	2279.31	4557.37	4083.63	3743.34	1449.09	2610.24	1445.23	205.31	408.42	822.98	1059.56	388.41	158.02	296.35	345.95	447.46
DTW	951.26	749.55	3787.13	1354.82	1335.13	1301.53	349.8	739.77	2347.04	11468.91	9738.26	10325.21	25982.46	20763.23	15619.02	4257.2	17252.45	6154.58	700.5	1836.85	6470.03	7815.71	2527.58	803.3	482.87	920.36	656.27
SEr	0.01	0.78	0.94	0.92	0.92	0.91	0.29	0.55	0.89	0.86	0.67	0.44	0.79	0.68	0.56	0.18	0.49	0.1	0.5	0.5	1	1	0.77	0.23	0.31	0.74	0.76

b)

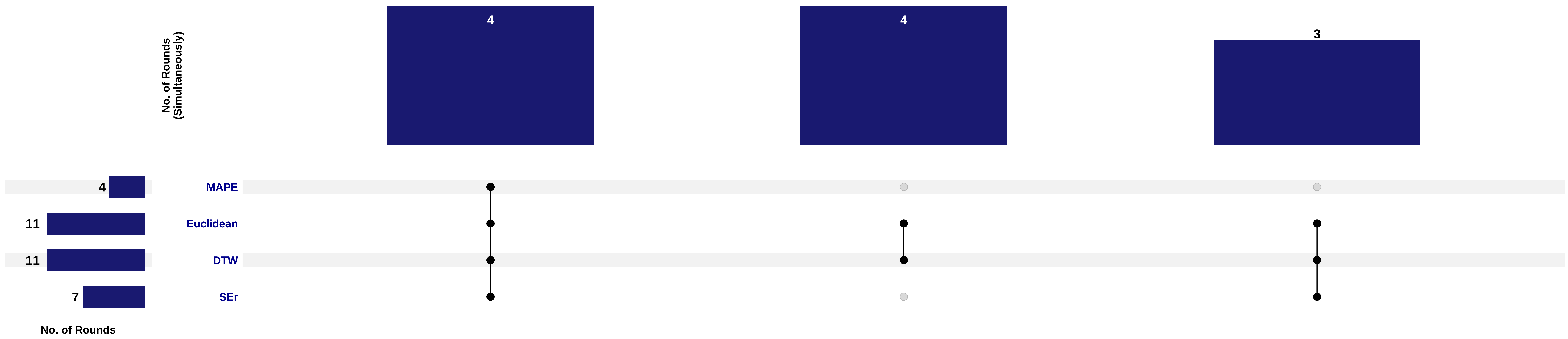

Region: Uppsala

a)

Similar	Not Similar

	Round 3			Round 4			Round 5			Round 6			Round 7			Round 8		Round 9	Round 10		Round 11		Round 12			Round 13	
	Scenario 0	Scenario 1	Scenario 2	Scenario 0	Scenario 1	Scenario 2	Scenario 0	Scenario 1	Scenario 2	Scenario 0	Scenario 1	Scenario 2	Scenario 0	Scenario 1	Scenario 2	Scenario 0	Scenario 1	Scenario 0	Scenario 0	Scenario 1	Scenario 0	Scenario 1	Scenario 0	Scenario 1	Scenario 2	Scenario 0	Scenario 1
MAPE	0.86	3.8	13.57	0.82	0.81	0.81	0.22	0.51	1.17	0.54	0.56	1.04	0.59	0.5	0.55	0.57	8.07	2.49	0.53	0.86	3.8	4.62	0.54	0.57	1.02	5.09	5.78
Euclidean	259.21	798.01	2184.34	184.65	183.81	182.61	74.58	136.94	300.91	2965.14	2709.25	2610.12	5608.87	5012.94	4620.12	2009.97	3472.4	1486.56	191.81	530.47	1130.13	1437.82	352.27	171.99	376.64	463.86	604.6
DTW	1456.46	1269.26	7875.25	2017.33	1974.56	1900.99	571.96	405.94	2049	15409.27	12508.84	11659.04	32487.93	26199.99	18765.57	4084.73	20873.66	6786.95	760.38	2167.64	9500.85	11722.4	2140.24	698	753.23	758.86	1308.17
SEr	0.15	0.83	0.95	0.91	0.9	0.9	0.3	0.53	0.97	0.86	0.65	0.43	0.79	0.67	0.56	0.17	0.49	0.09	0.45	0.44	1	1	0.7	0.31	0.53	0.84	0.93

b)

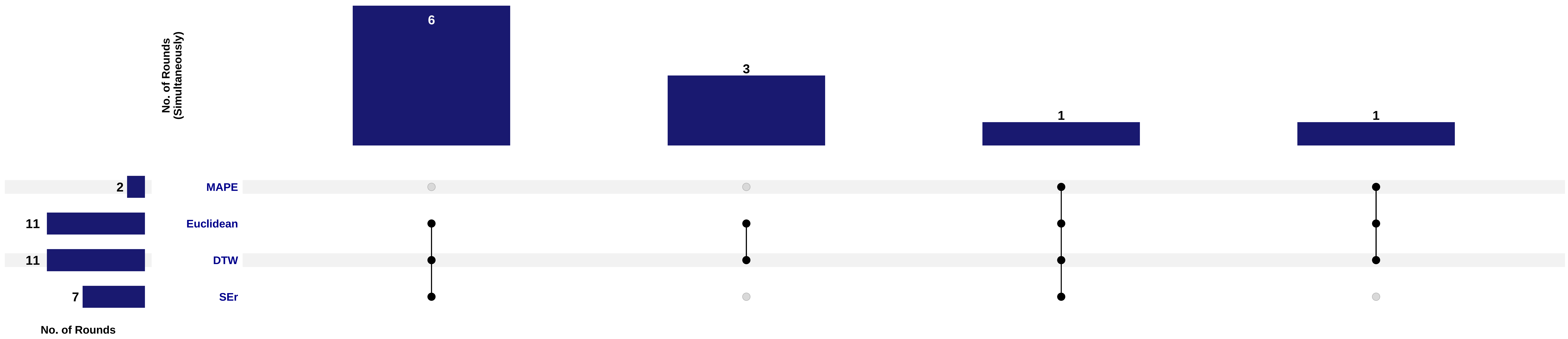

Region: Värmland

a)

Similar	Not Similar

	Round 3			Round 4			Round 5			Round 6			Round 7		
	Scenario 0	Scenario 1	Scenario 2	Scenario 0	Scenario 1	Scenario 2	Scenario 0	Scenario 1	Scenario 2	Scenario 0	Scenario 1	Scenario 2	Scenario 0	Scenario 1	Scenario 2
MAPE	0.68	1.01	4.15	0.75	0.72	0.68	2.32	4	6.39	0.67	0.83	1.5	0.74	0.65	0.59
Euclidean	256.27	356.89	1099.45	113.75	111.85	109.72	192.38	331.6	535.03	2334.04	2148.8	2075.42	5348.68	4930.42	4661.03
DTW	1662.01	1339.23	3702.52	540.88	514.17	481.5	1797.72	3082.03	5701.84	12061.34	10241.85	9953.09	32803.89	26449.08	20895.49
SEr	0.19	0.42	0.93	0.41	0.39	0.35	0.82	0.95	0.98	0.86	0.68	0.45	0.83	0.74	0.65

	Round 8		Round 9	Round 10		Round 11		Round 12			Round 13	
	Scenario 0	Scenario 1	Scenario 0	Scenario 0	Scenario 1	Scenario 0	Scenario 1	Scenario 0	Scenario 1	Scenario 2	Scenario 0	Scenario 1
MAPE	0.49	6.02	2.13	0.49	0.7	2.47	2.99	0.57	0.47	1.05	4.42	4.79
Euclidean	1878.98	2774.29	2070.98	161.26	362.97	754.46	978.2	372.16	169.39	318.53	301.14	393.38
DTW	5321.03	17292.97	7891.22	457.92	1492.95	6482.82	7755.13	2465.45	857.78	494.54	654.77	534.28
SEr	0.1	0.22	0.22	0.47	0.48	1	1	0.77	0.22	0.33	0.71	0.82

b)

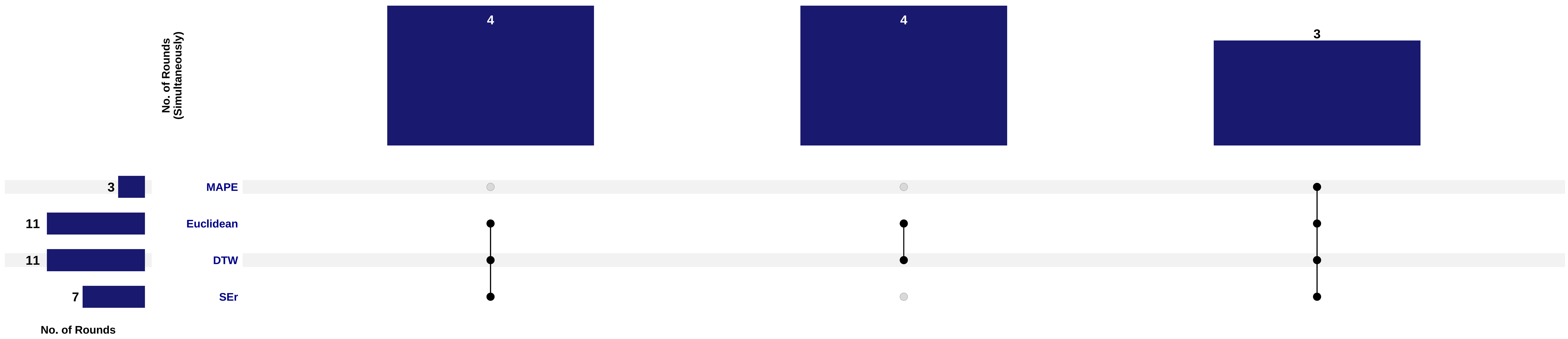

Region: Västerbotten

a)

Similar	Not Similar

	Round 3			Round 4			Round 5			Round 6			Round 7			Round 8		Round 9	Round 10		Round 11		Round 12			Round 13	
	Scenario 0	Scenario 1	Scenario 2	Scenario 0	Scenario 1	Scenario 2	Scenario 0	Scenario 1	Scenario 2	Scenario 0	Scenario 1	Scenario 2	Scenario 0	Scenario 1	Scenario 2	Scenario 0	Scenario 1	Scenario 0	Scenario 0	Scenario 1	Scenario 0	Scenario 1	Scenario 0	Scenario 1	Scenario 2	Scenario 0	Scenario 1
MAPE	0.47	1.75	6.6	0.77	0.76	0.76	1.32	2.39	3.88	0.41	0.62	1.51	0.73	0.73	0.82	0.78	8	2.74	0.47	0.71	2.34	2.88	0.56	0.61	0.76	6.44	7.34
Euclidean	193.24	274.08	790.46	242.42	241.66	240.52	324.86	602.98	998.44	1665.15	1515.14	1510.96	3209.85	2810.02	2598.19	1810.62	2692.13	877.47	165.87	362.07	727.63	944.95	168.96	142.05	242.9	360.74	460.98
DTW	1440.02	1301.41	2214.19	2052.2	2020.64	1966.58	3611.59	8063.95	13617.05	7670.71	6376.81	6672.24	20874.98	15283.47	10264.52	4749.66	16335.03	3940.96	592	1551.85	5841.59	7056.63	1115.63	455.79	885.03	1636.05	2223.28
SEr	0.29	0.18	1	0.91	0.91	0.91	0.73	0.87	0.87	0.83	0.58	0.34	0.78	0.65	0.5	0.29	0.61	0.08	0.47	0.44	1	1	0.66	0.34	0.71	0.8	0.76

b)

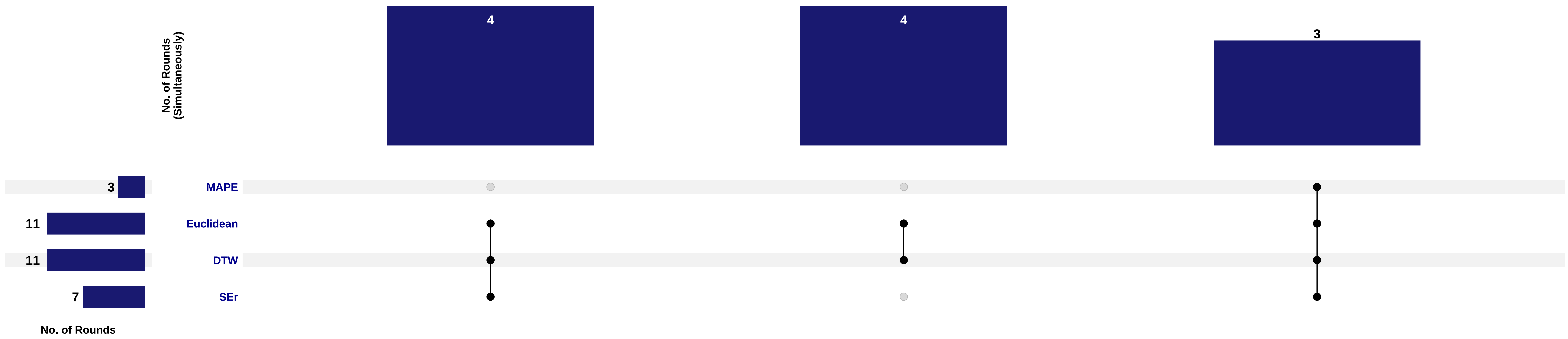

Region: Västernorrland

a)

Similar | Not Similar

	Round 3			Round 4			Round 5			Round 6			Round 7			Round 8		Round 9	Round 10		Round 11		Round 12			Round 13	
	Scenario 0	Scenario 1	Scenario 2	Scenario 0	Scenario 1	Scenario 2	Scenario 0	Scenario 1	Scenario 2	Scenario 0	Scenario 1	Scenario 2	Scenario 0	Scenario 1	Scenario 2	Scenario 0	Scenario 1	Scenario 0	Scenario 0	Scenario 1	Scenario 0	Scenario 1	Scenario 0	Scenario 1	Scenario 2	Scenario 0	Scenario 1
MAPE	1.59	8.12	29.96	0.79	0.78	0.77	0.69	1.42	2.54	0.73	0.95	1.79	0.62	0.58	0.69	0.7	7.32	2.95	0.62	1.03	3.56	4.34	0.61	0.34	0.72	5.42	5.92
Euclidean	289.5	814.48	2107.55	67.19	66.86	66.4	52.91	107.2	193.64	1390.82	1240.94	1216.72	2965.03	2618.39	2442.36	1635.03	2413.65	654.44	158.26	356.68	699.04	893.84	193.85	92.39	215.72	282.87	369.74
DTW	731.76	891.38	8474.29	547.58	544.18	539.22	376.02	665.41	1756.97	7384.36	5675.52	5707.58	16959.26	13545.04	9440.86	2803.48	13339.51	3294.53	996.71	1956.56	5505.46	6891.24	1401.43	399.59	841.62	997.57	1486.78
SEr	0.41	0.83	1	0.88	0.88	0.88	0.42	1	1	0.84	0.6	0.39	0.77	0.63	0.5	0.3	0.67	0.16	0.41	0.46	1	1	0.71	0.36	0.63	0.68	0.85

b)

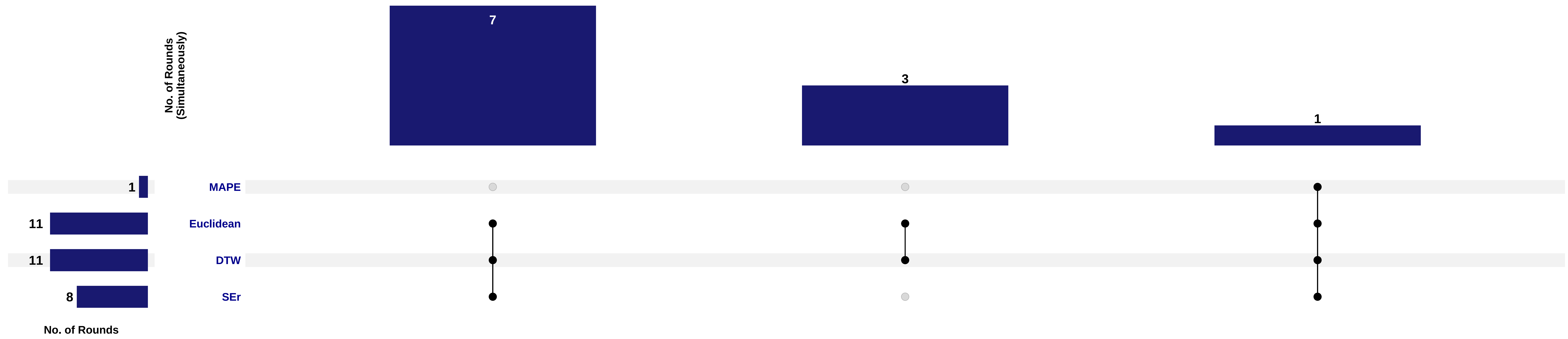

Region: Västmanland

a)

Similar	Not Similar

	Round 3			Round 4			Round 5			Round 6			Round 7		
	Scenario 0	Scenario 1	Scenario 2	Scenario 0	Scenario 1	Scenario 2	Scenario 0	Scenario 1	Scenario 2	Scenario 0	Scenario 1	Scenario 2	Scenario 0	Scenario 1	Scenario 2
MAPE	0.55	3.04	11.55	0.89	0.89	0.89	0.28	0.48	1	0.76	0.52	0.32	0.72	0.63	0.6
Euclidean	193.49	375.16	1078.15	218.96	218.08	216.79	177.73	179.77	363.16	2657.52	2451.09	2321.61	5400.06	4977.36	4688.66
DTW	1382.3	1309.77	1757.75	1864.52	1825.82	1759.47	1331.95	601.76	1311.46	16987.39	12600.33	11029.52	33325.25	27056.23	21735.66
SEr	0.18	0.42	0.94	0.89	0.89	0.89	0.35	0.29	0.79	0.9	0.75	0.58	0.83	0.74	0.65

	Round 8		Round 9	Round 10		Round 11		Round 12			Round 13	
	Scenario 0	Scenario 1	Scenario 0	Scenario 0	Scenario 1	Scenario 0	Scenario 1	Scenario 0	Scenario 1	Scenario 2	Scenario 0	Scenario 1
MAPE	0.47	11.12	3.88	0.52	0.99	4.35	5.36	0.51	0.64	1.15	5.93	6.7
Euclidean	1784.43	2705.12	2148.99	147.93	399.16	824.6	1046.11	237.04	143.74	296.38	328.15	427.26
DTW	6855.77	19165.07	9349.84	792.58	1832.93	7175.63	8722.85	1382.37	450.86	769.73	675.71	1133.55
SEr	0.11	0.21	0.21	0.43	0.51	1	1	0.69	0.31	0.68	0.7	0.8

b)

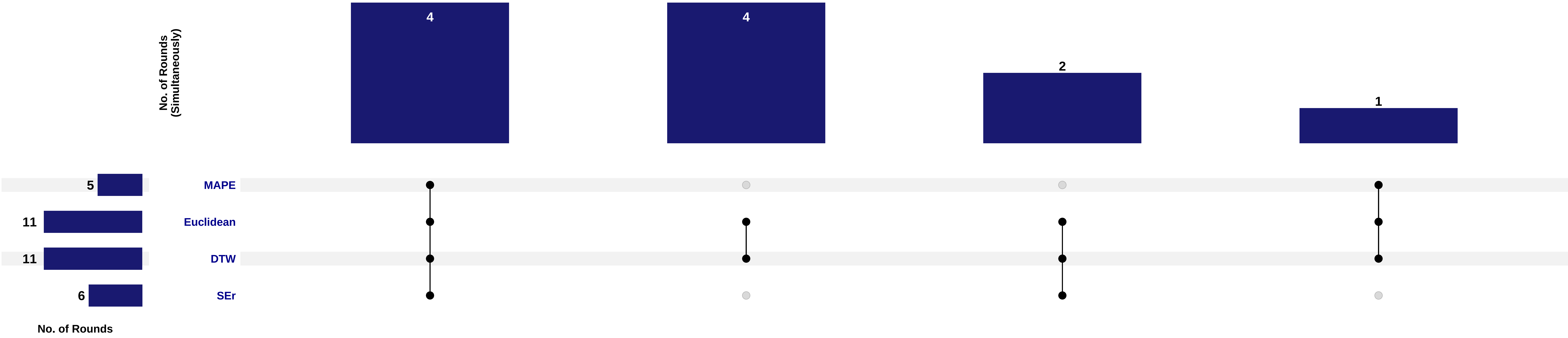

Region: Västra Götaland

a)

Similar	Not Similar

	Round 3			Round 4			Round 5			Round 6			Round 7			Round 8		Round 9	Round 10		Round 11		Round 12			Round 13	
	Scenario 0	Scenario 1	Scenario 2	Scenario 0	Scenario 1	Scenario 2	Scenario 0	Scenario 1	Scenario 2	Scenario 0	Scenario 1	Scenario 2	Scenario 0	Scenario 1	Scenario 2	Scenario 0	Scenario 1	Scenario 0	Scenario 0	Scenario 1	Scenario 0	Scenario 1	Scenario 0	Scenario 1	Scenario 2	Scenario 0	Scenario 1
MAPE	0.62	2.44	8.91	0.98	0.98	0.98	0.31	0.71	1.3	0.55	0.78	1.6	0.56	0.48	0.51	0.57	13.87	5.32	0.64	1.35	7.32	8.75	0.58	1.7	2.57	7.19	8.17
Euclidean	951.48	2607.78	7900.86	1323.93	1323.45	1322.78	508.45	812.14	1514.4	13968.39	12864.76	12484.41	29573.48	26945.13	25151.85	10335.91	16458.09	11021.45	794.4	2532.39	5481.69	6842.39	1021.19	1340.18	2213.18	2264.05	2901.76
DTW	8649.46	7617.83	29999.55	13873.72	13848.37	13804.77	3329.02	4022.99	8182.82	69923.4	59834.18	61706.89	176203.33	145913.91	113930.55	23950.43	102921.09	39750.47	5275.63	11564.61	53675.69	63631.64	5796.87	5040.08	9601.57	6928.36	10350.38
SEr	0.13	0.66	0.96	0.99	0.99	0.99	0.19	0.43	0.86	0.86	0.65	0.4	0.82	0.71	0.61	0.08	0.37	0.08	0.49	0.98	1	1	0.51	0.83	0.95	0.87	0.87

b)

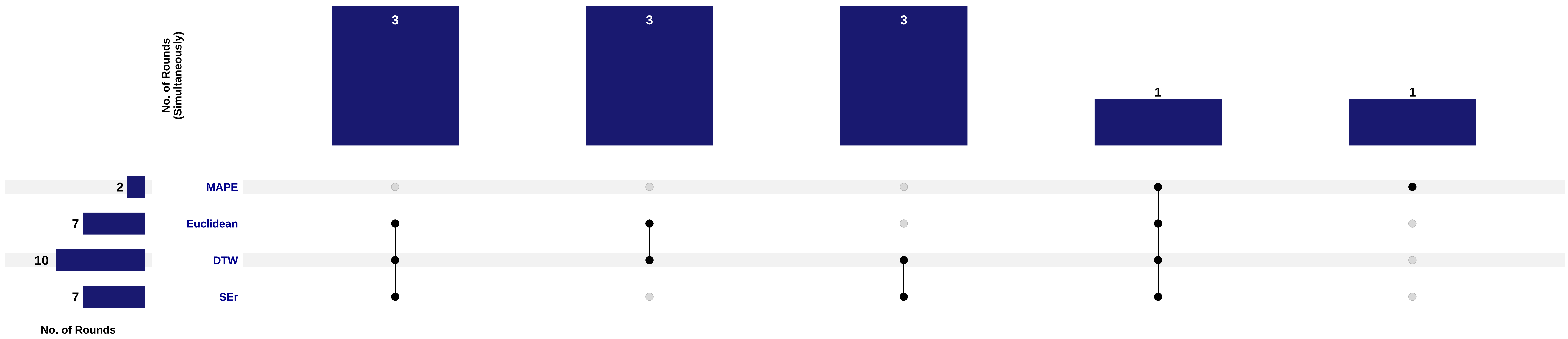

Region: Örebro

a)

Similar	Not Similar

	Round 3			Round 4			Round 5			Round 6			Round 7		
	Scenario 0	Scenario 1	Scenario 2	Scenario 0	Scenario 1	Scenario 2	Scenario 0	Scenario 1	Scenario 2	Scenario 0	Scenario 1	Scenario 2	Scenario 0	Scenario 1	Scenario 2
MAPE	0.74	3.83	14.22	0.76	0.76	0.75	0.22	0.39	0.85	0.61	0.44	0.74	0.74	0.67	0.64
Euclidean	166.35	591.72	1726.56	196.16	195.45	194.43	112.02	111.1	222.78	2986.68	2788.79	2694.08	7462.17	7018.25	6728.71
DTW	1016.48	884.56	5472.08	1673.03	1644.08	1594.56	775.91	556.53	764.56	15150.86	12558.21	11871.78	45341.99	38343.9	31779.36
SEr	0.05	0.81	1	0.92	0.92	0.92	0.39	0.26	0.75	0.88	0.71	0.53	0.85	0.78	0.7

	Round 8		Round 9	Round 10		Round 11		Round 12			Round 13	
	Scenario 0	Scenario 1	Scenario 0	Scenario 0	Scenario 1	Scenario 0	Scenario 1	Scenario 0	Scenario 1	Scenario 2	Scenario 0	Scenario 1
MAPE	0.54	4.74	1.5	0.61	0.75	1.33	1.69	0.73	0.35	0.58	3.62	3.86
Euclidean	3326.68	3979.75	3848.87	298.45	438.18	706.01	952.03	555.84	286.25	374.48	346.03	429.72
DTW	12244.36	24861.11	16774.74	922.1	1925.89	4856.58	6635.62	4293.98	1966.44	991.44	1207.84	1054
SEr	0.25	0.13	0.36	0.63	0.58	0.89	0.86	0.83	0.32	0.24	0.67	0.84

b)

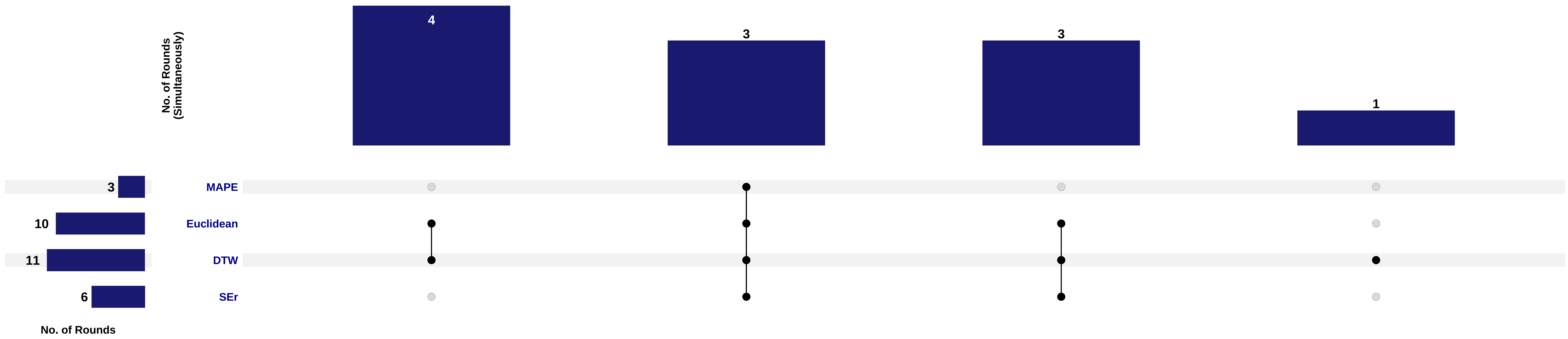

Region: Östergötland

a)

Similar | Not Similar

	Round 3			Round 4			Round 5			Round 6		
	Scenario 0	Scenario 1	Scenario 2	Scenario 0	Scenario 1	Scenario 2	Scenario 0	Scenario 1	Scenario 2	Scenario 0	Scenario 1	Scenario 2
MAPE	0.91	4.5	16.33	0.8	0.79	0.79	0.4	0.97	1.81	0.74	1.52	3.04
Euclidean	306.3	1046.19	2932.51	231.51	230.67	229.52	86.73	198.6	371.58	2655.35	2430.65	2476.03
DTW	1648.74	1318.59	7304.92	2246.01	2207.63	2141.96	492.2	1074.95	3227.54	12034.57	10400.96	11160.07
SEr	0.06	0.82	0.92	0.92	0.92	0.91	0.25	0.63	0.86	0.79	0.49	0.28

	Round 7			Round 8		Round 9	Round 10	
	Scenario 0	Scenario 1	Scenario 2	Scenario 0	Scenario 1	Scenario 0	Scenario 0	Scenario 1
MAPE	0.64	0.63	0.78	0.85	11.98	4.66	0.37	0.7
Euclidean	6542.44	5908.98	5597.33	3111.26	4636.6	1649.88	148.48	611.5
DTW	36735.82	29639.06	22224.03	4858.74	26037.39	8333.99	672.04	2365.31
SEr	0.78	0.65	0.52	0.28	0.62	0.15	0.37	0.68

	Round 11		Round 12			Round 13	
	Scenario 0	Scenario 1	Scenario 0	Scenario 1	Scenario 2	Scenario 0	Scenario 1
MAPE	4.27	5.04	0.48	1.45	2.09	5.87	6.89
Euclidean	1367.97	1733.72	246.52	348.15	564.95	626.48	799.17
DTW	13068.8	15058.27	1284.89	1246.42	2346.38	2367.31	3344.44
SEr	1	1	0.53	0.73	0.88	0.9	0.94

b)

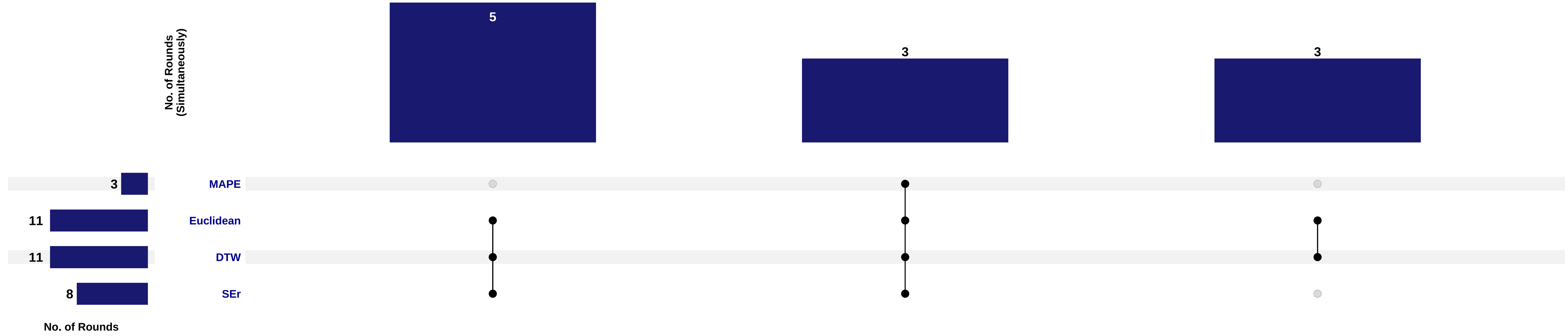

Supplement: Supplementary file 1 — Supplementary Material 1 [file 41598_2025_8682_MOESM1_ESM.pdf]
